# Synthesis of Versatile DNA‐Conjugated Aldehydes by Controlled Oxidation of Amines

**DOI:** 10.1002/anie.202507064

**Published:** 2025-06-16

**Authors:** Guixian Zhao, Mengping Zhu, Pengyang He, Qigui Nie, Yangfeng Li, Gong Zhang, Yizhou Li

**Affiliations:** ^1^ Chongqing University FuLing Hospital Chongqing University Chongqing P.R. China; ^2^ Chongqing Key Laboratory of Natural Product Synthesis and Drug Research Innovative Drug Research Center School of Pharmaceutical Sciences Chongqing University Chongqing 401331 P.R. China; ^3^ Chemical Biology Research Center School of Pharmaceutical Sciences Chongqing University Chongqing 401331 P.R. China

**Keywords:** Aldehyde‐functionalized DNA, Bioconjugation, Controlled oxidation, DNA modification, DNA‐encoded libraries

## Abstract

Aldehyde‐functionalized oligonucleotides have found diverse applications in chemical biology and material science. However, due to the electrophilic nature of aldehydes, incorporating aldehyde functionalities directly into DNA is challenging, particularly for highly reactive alkyl aldehydes. Inspired by natural oxidases, we herein developed a controlled oxidation strategy to generate aldehyde‐functionalized DNAs from synthetically accessible DNA‐conjugated amines in situ. A broad range of DNA‐conjugated alkyl and aryl aldehydes were efficiently produced from the corresponding amines using O_2_/laccase/TEMPO, with feasible micromole‐scale preparation. Moreover, combining oxidative cleavage of DNA‐conjugated secondary and tertiary amines with reductive amination enabled switchable amine–aldehyde transformation and reversible solid‐phase bioconjugation of DNA probes. Furthermore, the reactivity “umpolung” from nucleophilic amines to electrophilic aldehydes highlights its potential for synthesizing chemically diverse DNA‐encoded libraries (DELs). In summary, the presented controlled oxidation strategy expands the current toolbox to introduce aldehyde functionalities into DNAs within a chemical biological context.

## Graphical abstract

A controlled oxidation strategy enables efficient in situ generation of aldehyde‐functionalized DNAs from DNA‐conjugated amines using O_2_/laccase/TEMPO. This approach facilitates reversible amine–aldehyde transformations for DNA bioconjugation, and provides access to chemically diverse DNA‐encoded libraries, broadening the toolbox for chemical biology and material science.

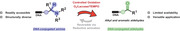

## Introduction

**Figure 1:**
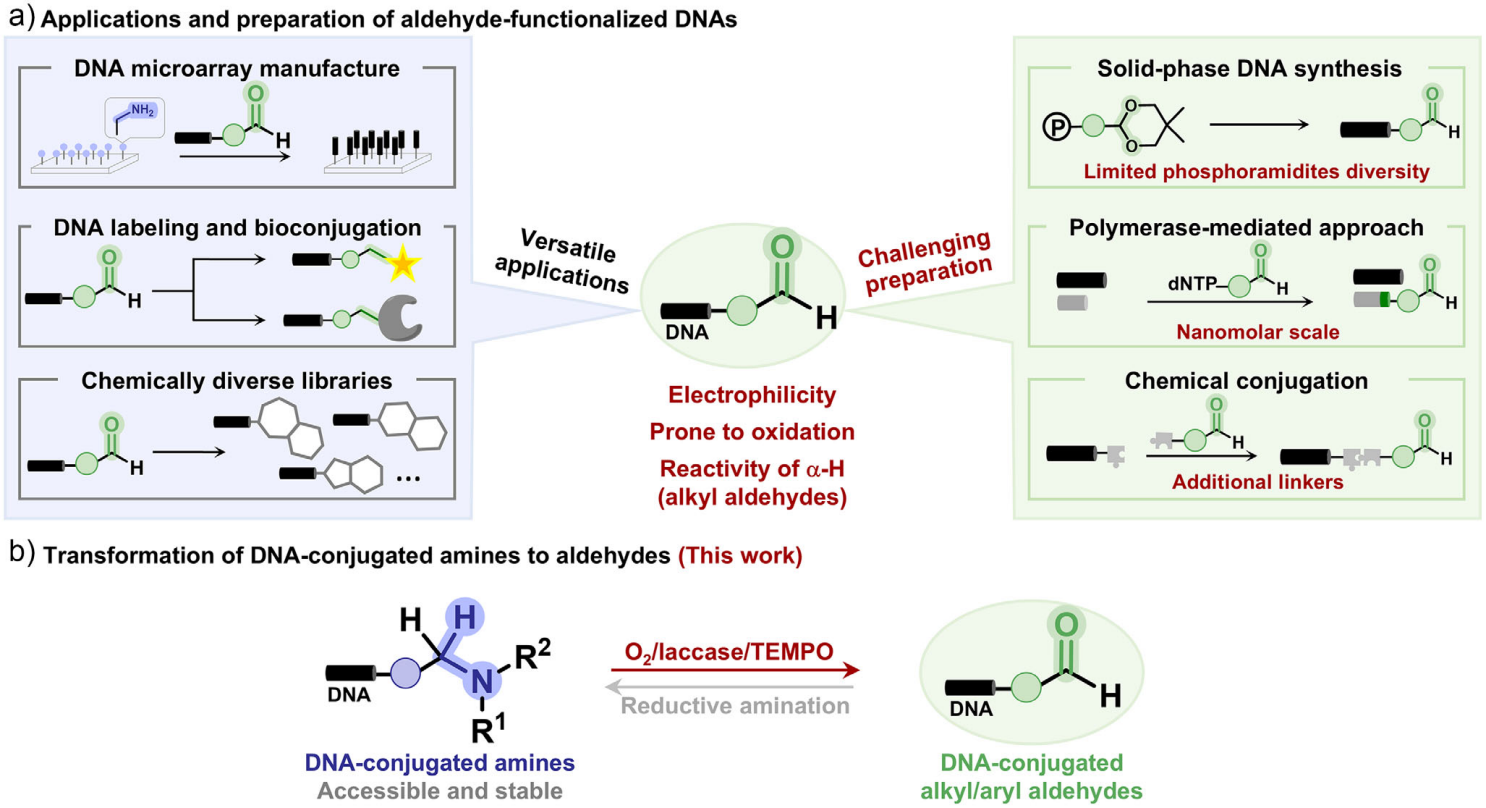
Applications and synthetic approaches for aldehyde‐functionalized DNAs. a), Chemical properties of DNA‐conjugated aldehydes, application of aldehyde‐functionalized DNAs, and previous strategies for incorporating aldehydes into DNAs. b), A controlled oxidation strategy to generate DNA‐conjugated aldehydes in situ from amines by O_2_/laccase/TEMPO.

Chemical modification of oligonucleotides attracts intensive attention in the fields of chemical biology and material science.^[^ ^1^, ^2^, ^3^ ^]^ Beyond natural epigenetic marks,^[^ ^4^, ^5^ ^]^ the introduction of artificial reactive groups into DNA enables advances in bioconjugation, diagnostics,^[^ ^6^ ^]^ nanotechnology,^[^ ^7^ ^]^ and drug discovery.^[^ ^8^ ^]^ In this context, aldehyde‐functionalized DNA is particularly attractive due to the electrophilic nature of the aldehyde group, which contrasts with the nucleophilic functionalities common in biological systems.^[^ ^9^ ^]^ Aldehyde groups enable selective conjugation with hydrazines, hydroxylamines, or amines,^[^ ^10^ ^]^ facilitating applications such as DNA microarrays,^[^ ^11^ ^]^ affinity labeling,^[^ ^12^ ^]^ and epigenetic modification detection (5‐hydroxymethylcytosine /5‐formylcytosine).^[^ ^13^, ^14^ ^]^ Moreover, their chemical versatility supports the construction of drug‐like compound libraries (Figure 1a).^[^ ^15^, ^16^ ^]^ However, general strategies for the direct and diverse incorporation of aldehyde groups into DNA remain underdeveloped, owing to their electrophilicity and relative instability.

Solid‐phase oligonucleotide synthesis remains the primary strategy for efficient and scalable production of custom DNA probes.^[^ ^11^, ^17^ ^]^ Alternatively, aldehyde groups can be introduced via enzymatic methods, such as DNA polymerase‐mediated incorporation of modified dNTPs^[^ ^18^ ^]^ or uracil‐DNA glycosylase (UDG)‐induced formation of abasic sites.^[^ ^19^ ^]^ Chemical conjugation or transformation offers additional routes.^[^ ^20^ ^]^ However, these approaches face limitations, including the complexity of phosphoramidite synthesis, low scalability, inefficient coupling of alkyl aldehyde‐containing units, and the need for additional linkers. Notably, none have enabled the efficient incorporation of highly reactive alkyl aldehydes into DNA, due to their susceptibility to oxidation and aldol condensation. (Figure 1a). Thus, there is an urgent demand for practical methods to synthesize alkyl aldehyde‐functionalized DNA at preparative scale. To address this, we proposed the in situ generation of DNA‐conjugated aldehydes via direct, on‐DNA controlled oxidation of DNA‐linked amines.

This rationale is supported by two considerations. First, nature's use of controlled enzymatic oxidation to convert amines to carbonyls inspires biomimetic synthetic strategies. Examples include PLP‐dependent transamination,^[^ ^21^ ^]^ quinone‐mediated amine‐to‐ketone conversion,^[^ ^22^ ^]^ and copper‐dependent oxidative deamination.^[^ ^23^ ^]^ These enzymes enable selective amine‐to‐aldehyde oxidation under mild, biomolecule‐compatible conditions and can avoid over‐oxidation to carboxylic acids, a limitation of some chemical oxidants. Second, amine‐functionalized DNAs are stable, commercially available, and readily synthesized via scalable solid‐phase oligonucleotide synthesis. Their structural diversity can be expanded through coupling with amine‐containing building blocks, making them ideal precursors for aldehyde‐functionalized DNAs.^[^ ^24^ ^]^ Based on these advantages, we sought to develop biocompatible oxidation systems for efficient conversion of DNA‐linked amines into aldehydes.^[^ ^25^, ^26^ ^]^

In this study, we demonstrate a facile and straightforward protocol for in situ generation of both alkyl and aryl aldehyde‐functionalized DNAs through mild and controlled oxidation of DNA‐conjugated amines by O_2_/laccase/2,2,6,6‐tetramethylpiperidine‐1‐oxyl radical (TEMPO) (Figure 1b). This DNA‐compatible aldehyde generation strategy demonstrated its utility in biomolecule labeling and reversible solid‐phase DNA conjugation. Furthermore, by achieving the reactivity “umpolung” from nucleophilic amines to electrophilic aldehydes, this approach unlocks the potential for synthesizing DNA‐encoded libraries (DELs) with abundant chemical diversity which were previously hard to access.^[^ ^27^, ^28^, ^29^ ^]^ Overall, this controlled oxidation strategy could expand the chemical biology toolbox to introduce versatile aldehyde functionalities into DNAs.

## Results and Discussion

### Oxidative Transformation of DNA‐Conjugated Alkyl Amines into Alkyl Aldehydes

**Figure 2:**
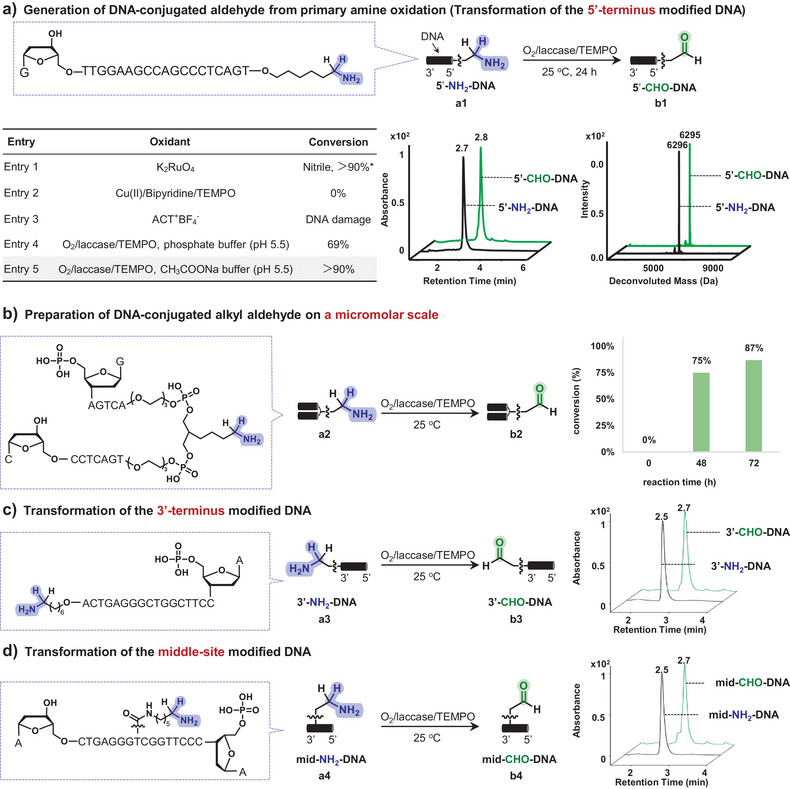
Oxidative transformation of DNA‐conjugated primary amines into aldehydes. a), O_2_/laccase/TEMPO enables mild, DNA‐compatible amine‐to‐aldehyde oxidation.b), Micromole‐scale preparation of DNA‐conjugated alkyl aldehydes using O_2_/laccase/TEMPO.c) and d), O_2_/laccase/TEMPO‐mediated oxidation of diverse formats of DNA‐conjugated primary amines. Standard conditions: DNA conjugate (0.2 nmol, 1 equiv.), CH_3_COONa buffer (16 µL, 200 mM, pH 5.5), laccase (2 µL, 0.1 U µL^−1^ in H_2_O), and TEMPO (2 µL, 400 mM in 1, 4‐dioxane, 800 nmol, 4000 equiv.). The reaction mixture was mixed, centrifuged, and incubated at 25 °C for 24 h.

Given the higher reactivity of alkyl aldehydes on DNA, we prioritized the development of a mild approach for the in situ generation of DNA‐conjugated alkyl aldehydes from a DNA‐conjugated aliphatic primary amine (5′‐NH_2_‐DNA, **a1**). Although our previous work showed that K_2_RuO_4_ oxidized benzyl alcohols in a DNA‐compatible manner,^[^ ^30^ ^]^ its application to amines resulted in nitrile formation rather than aldehydes, indicating over‐oxidation. (Figure 2a, entry 1). Other reported DNA‐compatible oxidation methods similarly failed,^[^ ^27^, ^31^ ^]^ resulting in either low conversion or DNA damage (Figure 2a, entries 2–3). To our delight, when we attempted the O_2_/laccase/TEMPO system (O_2_ from air or buffer), the desired DNA‐aldehyde conjugate was obtained successfully with 69% conversion (Figure 2a, entry 4). Subsequent optimization (Table S2, entries 1–10) revealed that buffer composition and temperature significantly influenced laccase activity.^[^ ^32^, ^33^ ^]^ Under the optimized condition of the slightly acidic CH_3_COONa buffer (pH = 5.5), the DNA‐conjugated aldehyde (5′‐CHO‐DNA, **b1**) was efficiently generated with over 90% conversion, without detectable DNA degradation or over‐oxidation (Figure 2a, entry 5). Notably, the aliphatic hydroxyl group on deoxyribose at the 3′‐terminus remained intact, confirming the high chemical selectivity of the established condition, which selectively oxidized amines while preserving alcohol groups. This was highly desired for selective modifications on biomacromolecules. All reactions were monitored by UPLC‐MS and product identity was further confirmed via aldehyde‐specific derivatizations (Figure S5).

To enable practical oligonucleotide synthesis, reactions must be scalable to the micromole level, consistent with solid‐phase DNA synthesis. We therefore performed the O₂/laccase/TEMPO‐mediated oxidation on 1 µmol of amine‐functionalized hairpin DNA (**a2**, commercially available as Headpiece). With 1 U laccase, 87% conversion to the aldehyde‐functionalized product was achieved after 72 h (Figures 2b, S7), demonstrating its applicability for preparative‐scale synthesis of aldehyde‐modified DNAs. Furthermore, bioconjugation of oligonucleotides at internal sites offers advantages such as improved structural stability and topological control. Beyond 5′‐amine modification, we evaluated O₂/laccase/TEMPO‐mediated oxidation of DNA bearing primary amines at the 3′‐terminus (**a3**, Figure 2c) and at an internal unnatural base (**a4**, Figure 2d). Both formats yielded efficient aldehyde formation (>90% conversion) while preserving native 3′‐ and 5′‐hydroxyl groups. These results underscore the versatility of this strategy, enabling facile access to aldehyde‐functionalized DNA probes using commercially available amine‐modified oligonucleotides.

### Substrate Investigation of Structurally Diverse DNA‐Conjugated Primary, Secondary, and Tertiary Amines

**Figure 3:**
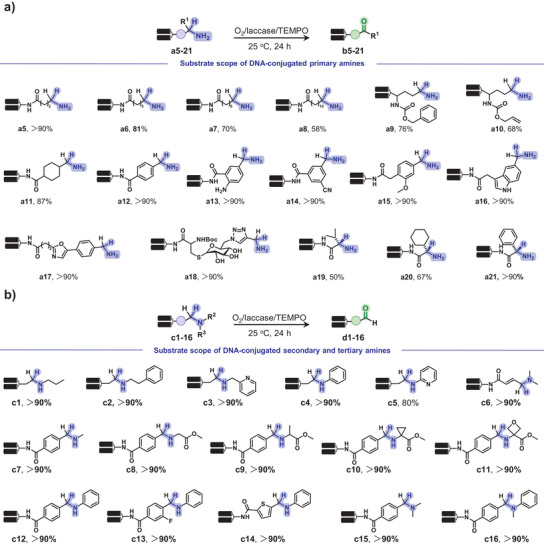
Substrate scope of DNA‐conjugated amines for O_2_/laccase/TEMPO‐mediated oxidation.a), Scope of primary amines.b), Scope of secondary and tertiary amines. Standard conditions: DNA conjugate (0.2 nmol, 1 equiv.), CH_3_COONa buffer (16 µL, 200 mM, pH 5.5), laccase (2 µL, 0.1 U µL^−1^ in H_2_O), and TEMPO (2 µL, 400 mM in 1, 4‐dioxane, 800 nmol, 4000 equiv.). The reaction system was mixed, centrifuged, and incubated at 25 °C for 24 h.

With the optimized oxidation conditions, we extensively investigated the substrate scope of DNA‐conjugated primary amines. In the O_2_/laccase/TEMPO system, TEMPO acts as the redox mediator, while laccase does not directly engage with substrates, suggesting broad substrate tolerance. We first assessed a series of aliphatic primary amines with diverse alkyl structures, all of which showed good to excellent oxidation efficiency (Figure 3a, **a5**–**a11**, 58% to >90%). Benzylamine derivatives were also efficiently converted to aryl aldehydes (**a12**–**a15**, >90%), including heterocyclic substrates such as indole (**a16**) and oxazole (**a17**). Notably, a glycoconjugated oligonucleotide containing multiple hydroxyl groups and a monothioacetal linker (**a18**) was also well tolerated (>90% conversion), indicating high chemoselectivity. We further explored α‐amino acid‐derived amines, hypothesizing ketone formation due to α‐substitution. As expected, both alkyl‐ and aryl‐substituted α‐amino acid derivatives (**a19**–**a21**) were oxidized to ketones (50% to >90%), confirmed by follow‐up reactivity assays (Figure S11). These results demonstrated that the O_2_/laccase/TEMPO system enables efficient synthesis of both aldehydes and α‐keto acids, offering a versatile platform for late‐stage peptide modification and α‐ketoacid–hydroxylamine ligation.^[^ ^34^ ^]^ Besides, we also systematically investigated and proved the stability of the generated DNA‐conjugated alkyl and aryl aldehydes (Supporting Information section 8).

We then examined the reactivity of DNA‐conjugated secondary and tertiary amines under O_2_/laccase/TEMPO‐mediated oxidation. DNA‐linked secondary amines (Figure 3b, **c1**–**c5**) and a tertiary amine (**c6**) derived from alkyl amines were efficiently converted to aldehydes via C─N bond cleavage, while the benzyl amine derivatives (**c7–c16**) also showed excellent conversions (>90%). This transformation is consistent with the proposed oxidation mechanism: for primary amines, removal of the α‐hydrogen leads to imine formation, followed by hydrolysis to yield aldehydes (or ketones in the case of α‐substituted amines). Secondary and tertiary amines undergo sequential C─N bond oxidation when α‐hydrogens are present, ultimately forming aldehydes. These results suggested that a broad range of amines could serve as precursors of diverse DNA‐conjugated aldehydes.

**Figure 4:**
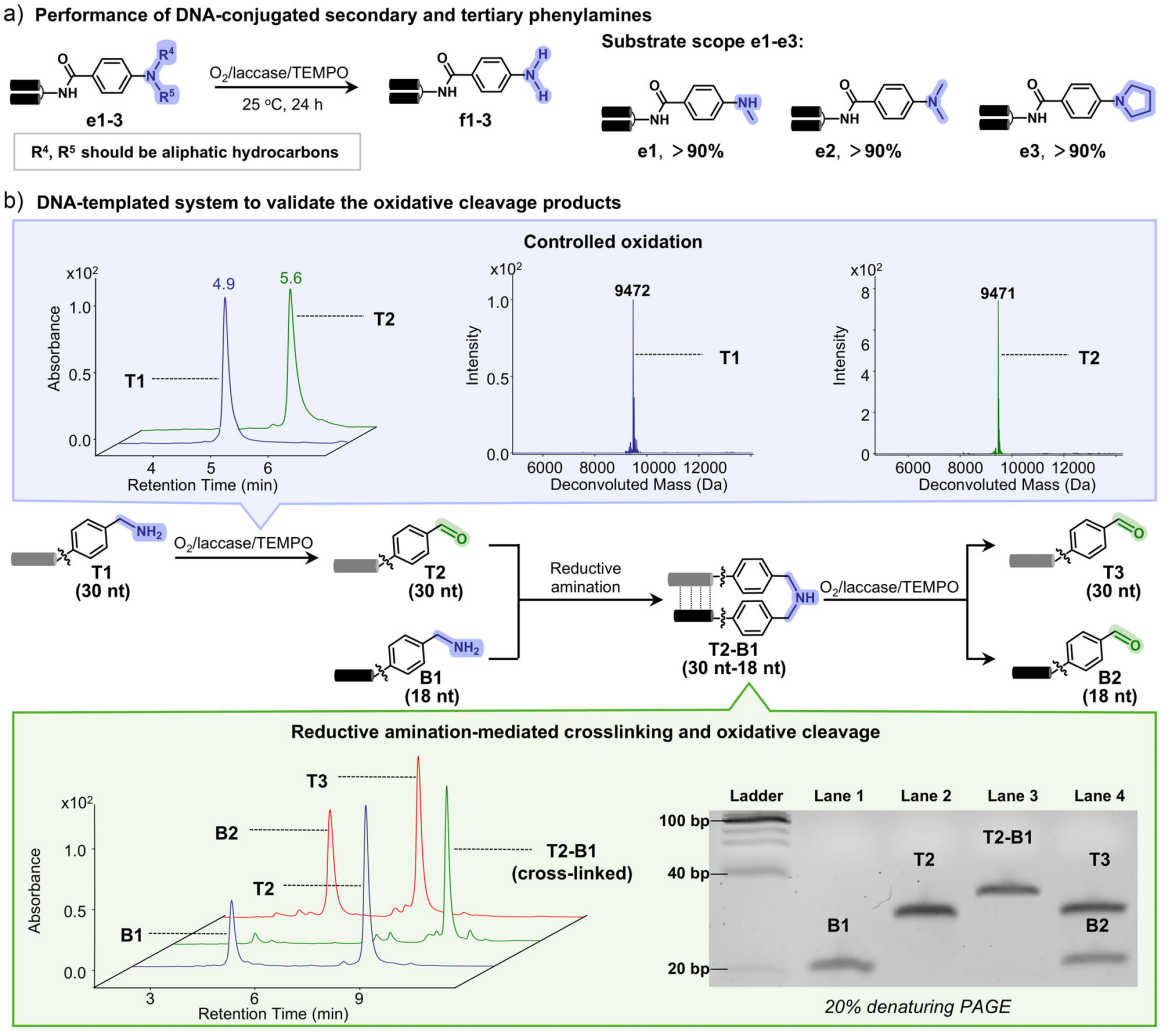
On‐DNA bond cleavage enabled by O_2_/laccase/TEMPO‐mediated oxidation. a), Oxidative deprotection of DNA‐conjugated secondary and tertiary phenylamines. b), Detection of oxidative cleavage products using a DNA‐templated system with amine‐conjugated DNA probes.

Following this vein, we next investigated the oxidation of DNA‐conjugated phenylamines, in which the nitrogen is directly attached to an aromatic ring and lacks an α‐hydrogen. For substituted secondary and tertiary phenylamines, the aliphatic N‐substituents were selectively cleaved, while the aryl C─N bond remained intact, preserving the DNA‐linked phenylamine core (Figure 4a, **e1**–**e3**; Figure S12). These results enabled a protection‐deprotection strategy for aryl amines on DNA through reductive amination–oxidative cleavage cycles.

These findings led us to hypothesize that the presence of an α‐hydrogen on DNA‐conjugated amines is a key determinant in laccase/TEMPO‐mediated oxidation. The resistance of aryl amines to oxidative cleavage, due to the absence of this hydrogen, supported this hypothesis. To further explore cleavage outcomes on both sides of the nitrogen atom, we designed a DNA‐templated system leveraging hybridization and proximity effects (Figure 4b). Complementary DNA probes bearing 5′‐ (**T1**) and 3′‐terminal (**B1**) benzylamine modifications were synthesized. Oxidation of **T1** yielded an aldehyde‐functionalized strand (**T2**), which was crosslinked with **B1** via DNA‐templated reductive amination (**T2‐B1**). Subsequent oxidation of the crosslinked product efficiently cleaved the C–N bond, producing **T3** and **B2**, as confirmed by gel electrophoresis. UPLC‐MS further verified aldehyde formation on both sides of the original secondary amine. Our systematic study here revealed promising potential for the switchable transformation between DNA‐linked amines and aldehydes.

### Bioconjugation of In Situ Generated Alkyl Aldehyde‐Functionalized DNA

**Figure 5:**
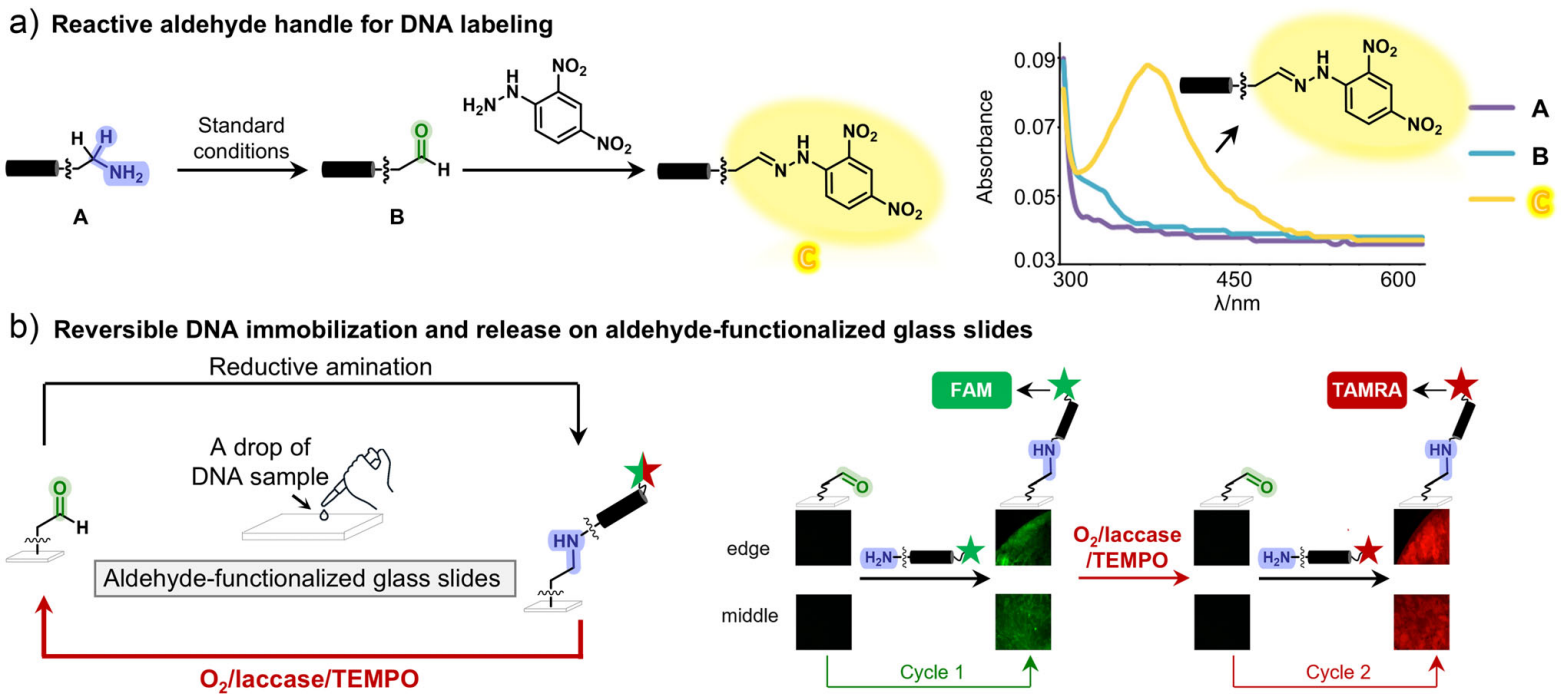
Bioconjugation applications of alkyl aldehyde‐functionalized DNAs. a), DNA labeling with DNP dyes enabled by the aldehyde handle. Standard conditions: DNA conjugate (0.2 nmol, 1 equiv.), CH_3_COONa buffer (16 µL, 200 mM, pH 5.5), laccase (2 µL, 0.1 U µL^−1^ in H_2_O), and TEMPO (2 µL, 400 mM in 1, 4‐dioxane, 800 nmol, 4000 equiv.). The reaction mixture was mixed, centrifuged, and incubated at 25 °C for 24 h. b), Reversible solid‐phase DNA conjugation and removal on glass slides achieved through cycles of reductive amination and O_2_/laccase/TEMPO‐mediated oxidative cleavage.

Building on the ability of O_2_/laccase/TEMPO to convert structurally diverse DNA‐conjugated amines into aldehydes, we explored its utility for bioconjugation applications.^[^ ^35^ ^]^ First, an 18‐nt amine‐terminated DNA strand was oxidized to generate an aldehyde‐functionalized probe, which was subsequently labeled with a hydrazine‐modified 2,4‐dinitrophenyl (DNP) dye. Successful conjugation was confirmed by a visible color change and a characteristic absorbance peak at 350–450 nm (Figure 5a). Moreover, DNA‐conjugated aldehydes were shown to undergo efficient oxime ligation with a model RGD‐like peptide and thiazolidine formation with an *N*‐terminal cysteine‐bearing peptide, demonstrating its utility in bioconjugation such as oxime and hydrazone ligation (Supporting Information 11.2).

DNA microarrays, a fundamental technique for detecting, analyzing, and quantifying specific nucleic acid sequences, rely on the conjugation of chemically modified oligonucleotides to solid supports like chips and glass slides.^[^ ^36^ ^]^ Most solid‐phase immobilization chemistry for preparation of microarrays involves reaction between electrophilic handles (e.g., aldehydes) on the glass surface with nucleophilic handles (e.g., amines) on oligonucleotides. In this way, an amine‐functionalized fluorescein amidite‐DNA (FAM‐DNA) probe was immobilized on aldehyde‐coated glass slides by reductive amination, producing strong green fluorescence. We then hypothesized that the resulting secondary amine linkage could be selectively cleaved by O_2_/laccase/TEMPO. Upon treatment, fluorescence was lost, indicating efficient bond cleavage. To confirm aldehyde regeneration, we performed a second labeling with an amine‐functionalized Tetramethylrhodamine‐DNA (TAMRA‐DNA) probe. The observed red fluorescence verified successful reconjugation (Figure 5b). The reversible DNA immobilization strategy conceptually parallels surface regeneration techniques in SPR (surface plasmon resonance) biosensors. In SPR, reversible probe detachment allows for multi‐cycle use of the same chip, reducing cost, avoiding residual signal interference, and enabling repeated kinetic measurements under different conditions.^[^ ^37^, ^38^ ^]^ Our system achieves similar functionality through redox‐controlled cleavage of DNA conjugates under mild conditions, providing a nucleic acid‐compatible route to dynamic surface reuse. In addition to SPR applications, reversible solid‐phase DNA immobilization has been widely employed in microfluidic platforms for the purification of dye‐labeled sequencing fragments.^[^ ^39^ ^]^ This underscores the broader relevance of reversible DNA attachment in analytical workflows such as microarrays, surface‐encoded screening, and reusable biochips.

### Synthesis of Chemically Diverse DELs Through Reactivity ‘Umpolung’ to Introduce DNA‐Conjugated Aldehydes

**Figure 6:**
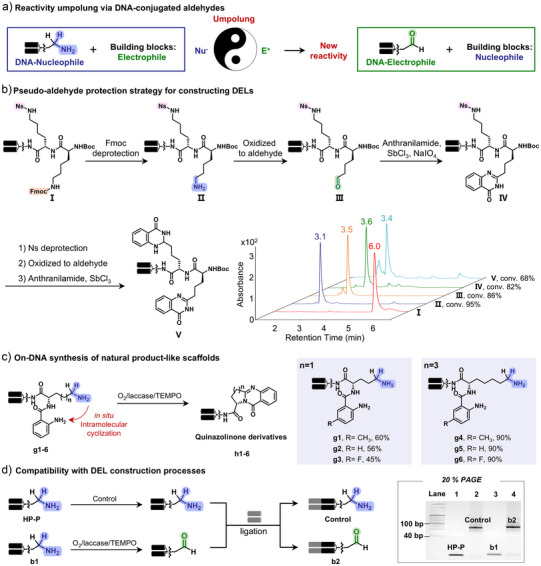
Synthesis of chemically diverse DELs through reactivity umpolung to introduce DNA‐conjugated electrophilic aldehydes.a), Reactivity umpolung enables electrophilic aldehyde incorporation on DNA. b), Aldehyde generation from protected amines allows multi‐display in DELs. c), In situ aldehyde formation enables DNA‐based quinazolinone synthesis. d), O_2_/laccase/TEMPO‐mediated oxidation demonstrated good compatibility with the barcoding process of DELs.

The oxidation of DNA‐conjugated amines to aldehydes represents a chemical reactivity umpolung, transforming nucleophilic amines into electrophilic aldehydes (Figure 6a). This reactivity switch broadens the synthetic utility of DNA‐conjugates. Given the power of DEL in hit discovery which combines chemical diversity with genetic barcoding,^[^ ^40^ ^]^ the O_2_/laccase/TEMPO‐mediated oxidation offered a DNA‐compatible transformation with strong potential to expand the structural and chemical space accessible in DEL synthesis.^[^ ^41^ ^]^

DEL construction typically involves multiple rounds of split‐and‐pool synthesis to append diverse building blocks (BBs) onto a DNA scaffold. The most widely used DNA starting material is the Headpiece (HP), a hairpin DNA bearing a flexible linker and terminal primary amine. The functional group on the DNA scaffold critically influences the chemical space accessible in the library. The O_2_/laccase/TEMPO‐mediated oxidation of amine‐containing HP to aldehyde‐functionalized DNA offers a powerful strategy for expanding DEL diversity. Aldehydes serve as versatile electrophilic handles, compatible with DNA‐friendly reactions such as reductive amination and heterocycle formation.^[^ ^15^, ^20^ ^]^ As shown in Figure 3, this system efficiently oxidizes a broad range of amines, suggesting that established amine‐based DELs could be transformed into aldehyde‐functionalized libraries. This umpolung reactivity shift—from nucleophilic amines to electrophilic aldehydes—enables subsequent derivatization with diverse nucleophiles (e.g., amines, phosphonium salts^[^ ^42^ ^]^), facilitating the generation of drug‐like scaffolds in DELs (Figures 6a, S13 and 14). We applied this workflow in a split‐pool setting, successfully synthesizing a 4 × 3 mock library to demonstrate the feasibility of aldehyde‐based diversification in DEL‐compatible conditions (Figure S21).

We demonstrated that amine‐to‐aldehyde oxidation enabled new DEL designs. Previous efforts have employed peptides with orthogonally protected amines to display carboxylic acid building blocks.^[^ ^43^ ^]^ However, despite large library sizes, chemical diversity remained limited to acid‐derived modifications. Aldehydes, in contrast, are highly reactive and versatile intermediates, yet their use in DELs has been hindered by the lack of orthogonal, DNA‐compatible protecting groups. To overcome this, we hypothesized that orthogonally protected amines on peptides could serve as precursors to selectively introduced aldehyde functionalities (Figure 6b). After Fmoc deprotection of a linear peptide (**I**), free amines were selectively oxidized to aldehydes using O_2_/laccase/TEMPO (**II** to **III**), as confirmed by UPLC‐MS. The resulting aldehydes were then derivatized into drug‐like heterocycles (**IV**). Subsequent removal of the Nosyl (Ns) protecting group enabled a second oxidation and derivatization step (**V**) (Figures S18–S20), further enhancing scaffold diversity. This approach, termed a “pseudo‐aldehyde protection strategy,” offered an efficient and modular method for constructing DELs centered on aldehyde‐derived core scaffolds.

In addition, the aldehyde functionality is central to the construction of pharmacologically relevant scaffolds. In nature, aldehyde‐ or imine‐mediated cyclization is a key step in the biosynthesis of pyrrolizidine alkaloids. Inspired by this, we designed an oxidative cyclization cascade wherein in situ oxidation of an amine yields an aldehyde that undergoes intramolecular cyclization to form natural product‐like structures (Figure 6c). Using a branched lysine‐type linker, we prepared a DNA‐conjugated molecule bearing an anthranilic acid and an amine. Upon O_2_/laccase/TEMPO oxidation, the amine was converted to an aldehyde, which spontaneously cyclized to form a tricyclic quinazolinone alkaloid.

Furthermore, the compatibility of O_2_/laccase/TEMPO‐mediated oxidation with DEL encoding was thoroughly evaluated. To assess DNA integrity, enzymatic ligation was performed on oxidized DNA‐conjugated amines. To our delight, all ligation reactions proceeded efficiently, as confirmed by PAGE analysis (Figure 6d). These results confirmed that aldehyde‐functionalized DNA headpieces could be prepared without compromising barcode fidelity, and that amine‐to‐aldehyde transformations are applicable to existing DELs.

## Conclusion

In conclusion, although aldehyde‐functionalized oligonucleotides offer broad synthetic utility, their incorporation into DNA remains challenging and underexplored. To address this, we developed a mild and efficient strategy for the in situ generation of aldehyde‐functionalized DNAs by O_2_/laccase/TEMPO mediated oxidation of DNA‐conjugated amines. A comprehensive study of primary, secondary, and tertiary amines demonstrated the method's broad substrate scope and chemoselectivity. This approach enabled micromole‐scale synthesis of alkyl aldehyde‐DNAs and was compatible with diverse amine positions on the oligonucleotide. The resulting aldehyde handle facilitated bioconjugation and reversible solid‐phase DNA conjugation. Furthermore, the strategy enabled a reactivity umpolung from nucleophilic amines to electrophilic aldehydes, expanding the chemical space in DEL synthesis. We demonstrated its application in DEL diversification and bioactive scaffold formation. Overall, this platform broadens the utility of aldehyde‐functionalized DNAs and offers new opportunities across chemical biology, materials science, and medicinal chemistry.

## Conflict of Interests

The authors declare no conflict of interest.

## Supporting information



Supporting Information

## Data Availability

The data that support the findings of this study are available in the supplementary material of this article.
