# Supplementary material for: Synthesis of Versatile DNA‐Conjugated Aldehydes by Controlled Oxidation of Amines
Source: Angew Chem Int Ed Engl. 2025 Jun 16;64(32):e202507064. doi: 10.1002/anie.202507064 (PMC12322624; doi:10.1002/anie.202507064)
Supplement: Supplementary file 1 — Supporting Information [file ANIE-64-e202507064-s001.pdf]

Supporting Information
©Wiley-VCH 2022
69451 Weinheim, Germany

Synthesis of Versatile DNA-Conjugated Aldehydes by Controlled Oxidation of Amines

Guixian Zhao,[a, b] + Mengping Zhu,[b] + Pengyang He,[b] + Qigui Nie,[a, b] Yangfeng Li,[b, c] Gong Zhang,* [b,c] and Yizhou Li *[b, c]

[a] G. Zhao, Q. Nie
Chongqing University FuLing Hospital, Chongqing University, Chongqing, China

[b] G. Zhao, M. Zhu, P. He, Q. Nie, Y. Li, G. Zhang, Y. Li
Chongqing Key Laboratory of Natural Product Synthesis and Drug Research, Innovative Drug Research Center, School of Pharmaceutical Sciences, Chongqing University, Chongqing 401331, P. R. China
E-mail: yizhouli@cqu.edu.cn (Yizhou Li), gongzhang@cqu.edu.cn (Gong Zhang)

[c] Y. Li, G. Zhang, Y. Li
Chemical Biology Research Center, School of Pharmaceutical Sciences, Chongqing University, Chongqing 401331, P. R. China.

[+] These authors contribute equally to this work.

Abstract: Aldehyde-functionalized oligonucleotides have found diverse applications in chemical biology and material science. However, due to the electrophilic nature of aldehydes, incorporating aldehyde functionalities directly into DNA is challenging, particularly for highly reactive alkyl aldehydes. Inspired by natural oxidases, we herein developed a controlled oxidation strategy to generate aldehyde-functionalized DNAs from synthetically accessible DNA-conjugated amines *in situ*. A broad range of DNA-conjugated alkyl and aryl aldehydes were efficiently produced from the corresponding amines using O_2/laccase/TEMPO, with feasible micromole-scale preparation. Moreover, combining oxidative cleavage of DNA-conjugated secondary and tertiary amines with reductive amination enabled switchable amine–aldehyde transformation and reversible solid-phase bioconjugation of DNA probes. Furthermore, the reactivity 'umpolung' from nucleophilic amines to electrophilic aldehydes highlights its potential for synthesizing chemically diverse DNA-encoded libraries (DELs). In summary, the presented controlled oxidation strategy expands the current toolbox to introduce aldehyde functionalities into DNAs within a chemical biological context.

Table of Contents

1. Abbreviations 4
2. Materials and General Methods 5
3. Preparation of DNA-conjugated substrates 8
 3.1 Preparation of DNA-conjugated substrates by amide condensation. 8
 3.2 Preparation of DNA-conjugated substrates by amination reaction. 8
 3.3 Preparation of DNA-conjugated amine by reductive amination 8
4. Experimental conditions for on-DNA oxidation to aldehyde 9
5. Structure validation 10
 5.1 Co-injection experiment 10
 5.2 On-DNA reductive amination 10
6. Scale-up of the reaction 11
7. Oxidation of three different DNA formats 13
 7.1 Primary amine group at the 5'-terminus 13
 7.2 Primary amine group at the 3'-terminus 13
 7.3 Primary amine group at the internal site 13
8. Stability test of DNA-conjugated aldehydes 15
 8.1 Chemical stability of DNA-conjugated aldehydes under storage conditions 15
 8.2 Chemical stability of DNA-conjugated aldehydes under physiological conditions 16
 8.3 Chemical stability of DNA-conjugated aldehydes in different buffers 17
 8.4 Chemical stability of DNA-conjugated aldehydes at different temperature 18
9. UPLC-MS Spectrum of different substrates 19
 9.1 Substrate scope of on-DNA primary amine oxidation 19
 9.2 Substrate scope of cleavage and oxidation of DNA-conjugated secondary or tertiary amines 36
 9.3 Reactivity validation of α-Carbon substituted substrates oxidized to ketones 52
 9.4 Substrate scope of cleavage of DNA-conjugated secondary or tertiary phenylamine 53
 9.5 Reactivity validation of the cleavage of DNA-conjugated secondary or tertiary phenylamines into phenylamines 56
10. DNA-templated system to validate the oxidative cleavage products 57
11. Bioconjugation of *in situ* generated aldehyde-functionalized oligonucleotides 58
 11.1 Reactive aldehyde handle for DNA labeling 58
 11.2 Conjugation of DNA-conjugated aldehyde with peptide 58
12. Aldehyde-based chemical derivatization reactions 60
 12.1 On-DNA benzimidazole formation 60
 12.2 On-DNA dihydroquinazolinone derivative formation 60
13. Reversible conjugation of DNA probes 61
14. Streamlined one-pot oxidation and diversification 62
15. Split-and-pool synthesis of a 4×3 mock library 64

16. Transformation of amines to aldehydes enabled aldehyde-based multiple display of DELs 65
17. Synthesis of quinazolinone alkaloid 71
17.1 On-DNA anthranilamide synthesis 71
17.2 Substrate expansion of quinazolinone alkaloid 71
17.3 UPLC-MS Spectrum of DNA-conjugated quinazolinone alkaloid 72
18. Compatibility of the oxidation reaction with DEL encoding 78
19. Substrate scope of DNA-conjugated alcohols 79
19.1 UPLC-MS Spectrum of DNA-conjugated alcohols 80
20. References 92

1. Abbreviations

BME: 2-mercaptoethanol

DBU: 1, 8-diazabicyclo [5.4.0] undec-7-ene

DIPEA: *N, N*-diisopropylethylamine

DMA: *N, N*-dimethylacetamide

DMSO: dimethyl sulfoxide

DMT-MM:4-(4, 6-dimethoxy-1, 3, 5-triazin-2-yl)-4-methylmorpholinium chloride

HATU: O-(7-aza-1-benzotriazolyl)-*N, N, N', N'* tetramethyluroniumhexafluorophosphate

EtOH: ethanol

FAM: 5(6)-Carboxyfluorescein

Fmoc: 9-fluorenylmethyloxycarbonyl

HFIP: 1, 1, 1, 3, 3, 3-hexafluoro-2-propanol

HP: headpiece

HP-P: headpiece primer

HPLC: high performance liquid chromatography

MeOH: methanol

MW: molecular weight

Ns: o-nitrobenzenesulfonyl

Nvoc: 6-nitroveratryloxycarbonyl

PAGE: polyacrylamide gel electrophoresis

PB: phosphate buffer

Ss HP-P: ss headpiece primer

TBE: tris-borate-EDTA

TCEP-HCl: tris(2-carboxyethyl) phosphine hydrochloride

TEAA: triethylammonium acetate

TEA: trimethylamine

TEMRA: 5-(and-6)-Carboxytetramethylrhodamine

TEMPO: 2, 2, 6, 6-tetramethylpiperidinooxy

Tfa: trifluoroacetyl

TFA: triethylamine

Tris-HCl: tris(hydroxylmethyl)aminomethane hydrochloride

UPLC-MS: Ultra-high performance liquid chromatography-mass spectrum

UV: ultraviolet

2. Materials and General Methods

2.1 Chemical materials

The Laccase enzyme (0.5 U/mg) from *Rhus vernificera* was purchased from Yingxin Laboratory Equipment Co., Ltd. Chemical building blocks and reagents were obtained from various commercial suppliers. (1 U corresponds to the amount of enzyme which converts 1 μmol catechol per minute at pH 6.0 and 25 °C)

2.2 DNA materials

This work utilized three types of DNA, the structures of which are depicted below: headpiece primer (HP-P, 5′/5Phos/ACCTTCGGGAGTCA/iSp9/iUniAmM/iSp9/TGACTCCCGACCGAAGGTTG-3′), ss headpiece primer (ss-HP-P, iUniAmM/iSp9/TGACTCCCGACCGAAGGTTG-3′) and headpiece (HP, 5′/5Phos/GAGTCA/iSp9/iUniAmM/iSp9/TGACTCCC-3′) and code sequence were received from HitGen Ltd. Headpiece, headpiece primer, ss headpiece primer DNA was stored in an aqueous solution (1 mM).

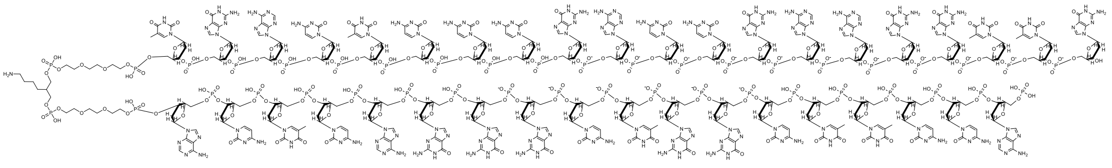

Figure S1. Structure of **HP-P**.

(5′/5Phos/ACCTTCGGGAGTCA/iSp9/iUniAmM/iSp9/TGACTCCCGACCGAAGGTTG -3′), MW=12409.

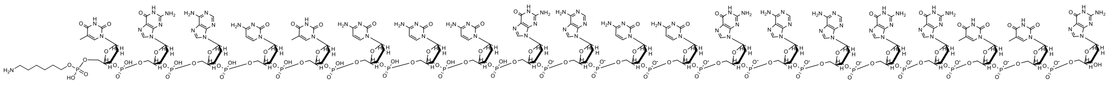

Figure S2. Structure of **ss-HP-P**.

(iUniAmM/iSp9/TGACTCCCGACCGAAGGTTG -3′), MW=6297.

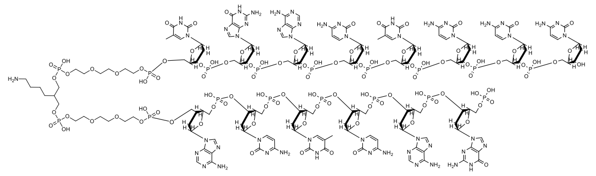

Figure S3. Structure of **HP**.

(5'/5Phos/GAGTCA/iSp9/iUniAmM/iSp9/TGACTCCC-3'), MW = 4937.

2.3 General methods for DNA conjugate purification

General method for DNA precipitation using ethanol: To the DNA reaction mixture, 10% (v/v) NaCl solution (5 M) is added, followed by three times the volume of cold ethanol (-20 °C). The solution is then placed at -80 °C for over 2 hours or in liquid nitrogen for ten minutes before being centrifuged at 13,500 rpm for 30 minutes at 4 °C. After discarding the supernatant, the pellet is washed once with cold 70% ethanol. Following another centrifugation at 13,500 rpm for 10 minutes at 4 °C, the supernatant is discarded and the pellet is dried using a SpeedVac. The collected sample is then dissolved in an appropriate buffer for further analysis or experiments. The Eppendorf 5424R centrifuge is utilized for the ethanol precipitation process of all DNA samples.

General method for HPLC purification: Preparative reversed-phase high-pressure liquid chromatography (RP-HPLC) for the DNA conjugate was performed on a Waters 1575EF Series with a reversed-phase HPLC column (Eclipse-XDB C18, 5 μM, 9.4×250 mm) using eluent A (100 mM TEAA in H_2O) and eluent B (100 mM TEAA in 80% MeCN) with gradient: 10% B (0 to 1 min), 10% to 30% B (1 to 11min), 30% to 100% B (11 to 11.1 min), 100% B (11.1 to 12 min), 100% to 10% B (12 to 12.1 min), 10% B (12.1 to 16 min). The fractions containing the product were combined and lyophilized overnight.

2.4 General procedure for DNA ligation

This reaction contained variably-derivatized HP-P starting material (10 nmol in H_2O, 1 equiv), code (12 nmol in H_2O, 1.2 equiv), 10× ligation buffer (4 μL), T4 DNA ligase (1 μL, 1000 units/μL) and nuclease-free water (to the total volume of 40 μL). The reaction was incubated at 20 °C overnight before performing gel analysis. The crude product was purified by ethanol precipitation and used for the

next step.

2.5 General procedure for polyacrylamide gel

The ligation reaction was monitored by gel electrophoresis with 20% urea polyacrylamide gel in 1× TBE buffer (89 mM Tris-Borate, 2 mM EDTA, pH 8.3) system referenced by a 20 bp DNA ladder (Takara, Japan). First, the DNA samples were denatured at 95 °C in a dry bath for 10 min and mixed with loading buffer. Then, 10 pmol of treated DNA samples was loaded on the gel, and the gel was run at 200 V for 50 - 60 min. DNA fragments were visualized and analyzed by Bio-Rad ChemidocTM Image System.

2.6 Reaction conditions of DNA-templated reactions

Reductive amination: 400 pmol of the substrate DNA (DPAL-BP-CHO and DPAL-CP-NH_2) were mixed with 400 µL of buffer (100 mM MES, 1.0 M NaCl, pH 6.0) in a 1.5 mL Eppendorf tube. The mixture was heated at 95 °C for 5 minutes and then slowly cooled to 25 °C over the course of 1 hour. Next, 2 µL of a 300 mM solution of $NaBH_3CN$ in 1 M NaOH was added to the solution, which was then briefly vortexed. The resulting solution was left at 25 °C for 14 hours, after which the DNA was recovered by ethanol precipitation.[1]

2.7 General methods for DNA analysis

On-DNA reaction analysis (UPLC-MS method). The detection was performed by a high-resolution mass spectrometry-Agilent 6230 Time-of-Flight (TOF) mass spectrometer connected to an Agilent 1290 UPLC. After the reaction, an aliquot of the reaction mixture was diluted with water to make the sample approximately 1 µM. Then, 10~20 µL of the sample was injected into a reversed-phase UPLC column (Agilent, AdvanceBio Oligonucleotide, C18, 2.1×50 mm, 2.7 µm, maintained at 60 °C) at a flow rate of 0.3 mL/min. The effluent was detected by UV absorbance (260 nm) and analyzed on Agilent 6230 TOF in negative ion mode.

UPLC-MS method of on-DNA synthesis of DNA-Conjugated Aldehyde:

Time (min)	Flow (mL/min)	%B
0	0.3	5
1	0.3	15
6	0.3	25
6.5	0.3	90
7	0.3	90
8	0.3	5

Solvent A: 200 mM HFIP and 8 mM TEA in H_2O; Solvent B: MeOH

UPLC-MS method of co-injection analysis:

Time (min)	Flow (mL/min)	%B
0	0.3	5
1	0.3	15
12	0.3	30
12.1	0.3	90
13	0.3	90
13.1	0.3	5
14	0.3	5

Solvent A: 200 mM HFIP and 8 mM TEA in H_2O; Solvent B: MeOH

UPLC-MS method of DNA ligation analysis:

Time (min)	Flow (mL/min)	%B
0	0.3	3
1	0.3	12
2.5	0.3	18
4	0.3	20
6	0.3	22
9	0.3	30
10	0.3	85
11	0.3	85
12	0.3	3

Solvent A: 200 mM HFIP and 8 mM TEA in H_2O; Solvent B: MeOH

Conversion calculation: the conversion of on-DNA product was determined from UV absorbance trace (260 nm) peak area by using the equation: conversion% = UV (product)/UV (total DNA recovered), ignoring the UV coefficient difference among all the DNA products and assuming 100% DNA recovery. Any non-oligo material that had an absorbance at UV 260 nm was subtracted from the conversion calculation.[2]

Analysis of molecular mass: observed m/z could be calculated as m/z = (m - z)/z. BioConfirm software (Agilent, v10.0) was used to deconvolute the multiple charge states.

3. Preparation of DNA-conjugated substrates

3.1 Preparation of DNA-conjugated substrates by amide condensation.

HP was dissolved in sodium borate buffer (250 mM, pH 9.4) to prepare a 1 mM solution. Carboxylic acid compound (17 µL, 200 mM in DMA, 170 equiv.), HATU (17 µL, 200 mM in DMA, 170 equiv.), and DIPEA (17 µL, 200 mM in DMA, 170 equiv.) were mixed by vortex and allowed to pre-activate for 10 minutes at 25 ºC, and then the mixture was transferred to **HP** solution (20 µL, 20 nmol). The reaction mixture was vortexed, centrifuged, and allowed to proceed at 25 ºC for 2 h. After ethanol precipitation, the reaction was analyzed by UPLC-MS.

The lyophilized pellet of DNA (20 nmol) was then deprotected using 100 mL of 10% v/v piperidine in water. The reaction mixture was vortexed, centrifuged, and allowed to proceed at 25 ºC for 20 min. After ethanol precipitation, the reaction was analyzed by UPLC-MS. The separated and collected conjugates were purified by preparative HPLC and vacuum-dried overnight, redissolved in H_2O for subsequent experiments.

Unless otherwise noted, amide bond formation on DNA and de-Fmoc described in the supporting information were performed under this standard condition.

3.2 Preparation of DNA-conjugated substrates by amination reaction.

To a solution of DNA-conjugates (5.0 µL, 1.0 mM in ddH_2O) were added KF_3BCH_2NHBoc (3.75 µL, 200 mM in DMA, 150 eq.) and K_2CO_3 (7.5 µL, 200 mM in ddH_2O, 300 eq.). After addition, the plates were centrifuged, eddied and centrifuged. And the solution was protected under N_2. Then the freshly prepared Pd$(OAc)_2$/rac-BIDME solution was added to the reaction mixture. (Note: Pd$(OAc)_2$ (5.0 µL, 10 mM in DMAc, 5 eq.), rac-BIDME (5.0 µL, 40 mM in DMAc, 20 eq.) The reaction was carried out under an N_2 atmosphere. The reaction mixture was heated at 95 °C for 2 h in PCR thermocycler. After reaction, 30 equivalents of sodium diethyldithiocarbamate were added to the mixture to scavenge Pd, relative to Pd$(OAc)_2$, and the reaction mixture was kept at 25 °C for 30 minutes. The mixture was centrifuged at 25 °C for 10 min at 13,500 rpm, and the resultant supernatant was collected. The product was purified by ethanol precipitation and analyzed using UPLC-MS.[3]

The lyophilized pellet of DNA (20 nmol) was reconstituted in sodium borate buffer (20 µL, 250 mM, pH 9.4, 250 eq.) and incubated at 90 °C for 16 hours. After ethanol precipitation, the reaction was analyzed by UPLC-MS. The purified conjugates were obtained by preparative HPLC, vacuum-dried overnight, and redissolved in H_2O for subsequent experiments.

3.3 Preparation of DNA-conjugated amine by reductive amination

A solution of HP-CHO (Oxidation HP by Laccase/TEMPO) (0.2 nmol, 4 µL) was diluted with phosphate buffer (200 mM, pH 5.5, 12 µL). To this reaction solution, amine (200 mM in DMA, 2 µL) was first added, followed by $NaBH_3CN$ (400 mM in water, 2 µL). The reaction mixture was incubated in the PCR at 60 °C for 8 hours. The product was obtained by ethanol precipitation and analyzed by UPLC-MS, with a conversion rate exceeding 90%.[4]

4. Experimental conditions for on-DNA oxidation to aldehyde

$ACT^+BF_4^-$ oxidation: 200 pmol DNA was incubated in 25 µL solution with 50 mM sodium phosphate buffer (pH 7.5) and 50 mM $ACT^+BF_4^-$(TCI) at 37 °C for 4 hours. The oxidized DNA was purified by ethanol precipitation and analyzed by UPLC-MS.[5]

Cu(II)/Bipyridine/TEMPO oxidation: To 1 nmol of DNA in 102 µL of NFW, 32 µL of 250 mM borate buffer (pH 9.5) and 100 equivalents of $Cu(ClO_4)_2$, bipyridine, and TEMPO (2,2,6,6-tetramethylpiperidine 1-oxyl) were added. Each reagent was used as a 5 mM stock solution (20 µL in MeCN), resulting in a total reaction volume of 194 µL. The reaction was vortexed and incubated for 12 hours at 25 °C. The oxidized DNA was purified by ethanol precipitation and analyzed by UPLC-MS.[6]

K_2RuO_4 oxidation: A solution of DNA (0.2 nmol, 2 µL, 100 µM in H_2O, 1 equiv.) was mixed with K_2RuO_4 (15 nmol, 1 µL, 15 mM in 500 mM NaOH, 75 equiv.) and H_2O (17 µL). The reaction mixture was vortexed, centrifuged, and incubated at 0 °C for 1 hour. The oxidized DNA was purified by ethanol precipitation and analyzed by UPLC-MS.[7]

Table S1. Synthesis of 5'-CHO-DNA.

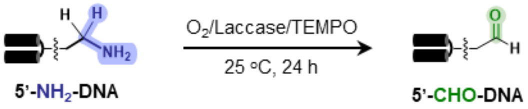

Entry	laccase	Solvent of TEMPO	buffer	Conversion (%)
1	laccase	1, 4-dioxane	CH_3COONa (pH 5.5)	>90
2	----	1, 4-dioxane	CH_3COONa (pH 5.5)	0
3	laccase	----	CH_3COONa (pH 5.5)	0
4[b]	laccase	1, 4-dioxane	CH_3COONa (pH 5.5)	83
5[c]	laccase	1, 4-dioxane	CH_3COONa (pH 5.5)	64
6	laccase	1, 4-dioxane	H_2O	0 (carboxylic acid)
7	laccase	1, 4-dioxane	PB (pH 5.5)	69
8	laccase	1, 4-dioxane	PB (pH 7.4)	0
9	laccase	1, 4-dioxane	PB (pH 8.0)	0
10	laccase	1, 4-dioxane	CH_3COONa (pH 4.0)	40
11	laccase	DMSO	CH_3COONa (pH 5.5)	66
12	laccase	DMA	CH_3COONa (pH 5.5)	80

b: 4 °C; c: 12 h

[a]**Standard condition:** DNA **a1** (0.2 nmol, 1 equiv.) was mixed with CH_3COONa buffer (16 µL, 200 mM, pH 5.5), Laccase (2 µL, 0.1 U/µL in H_2O), and TEMPO (2 µL, 400 mM in 1, 4-dioxane, 800 nmol, 4000 equiv.). The reaction mixture was vortexed, centrifuged, and incubated at 25 °C for 24 hours. The product was purified by ethanol precipitation and analyzed using UPLC-MS (conversion: greater than 90%). Deconvoluted molecular mass: calculated: 6295 Da; observed: 6295 Da. Unless otherwise noted, on-DNA amines described in the supporting information were synthesized under this standard condition.

5. Structure validation

5.1 Co-injection experiment

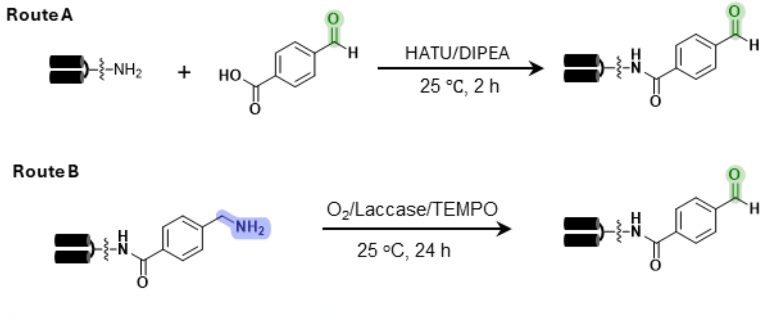

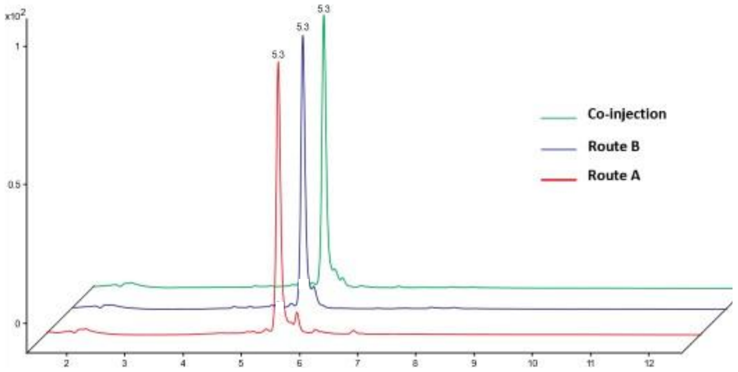

Figure S4. Co-injection experiment using two independent synthetic routes. UPLC analysis showed that the peak from the co-injection (green curve) had the same retention time as peaks from route A (red curve) and route B (blue curve).

5.2 On-DNA reductive amination

A solution of DNA-CHO (oxidized by Laccase and TEMPO, 0.2 nmol, 4 µL) was mixed with pH 5.5 phosphate buffer (200 mM in water, 12 µL). To this reaction solution, amine (200 mM in DMA, 2 µL) was first added, followed by $NaBH_3CN$ (2 µL, 400 mM in water). The reaction mixture was incubated in a PCR thermocycler at 60 °C for 8 hours. The product was purified by ethanol precipitation and analyzed using UPLC-MS, with a conversion rate exceeding 90%.[4]

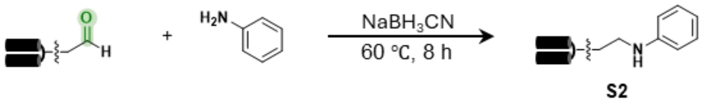

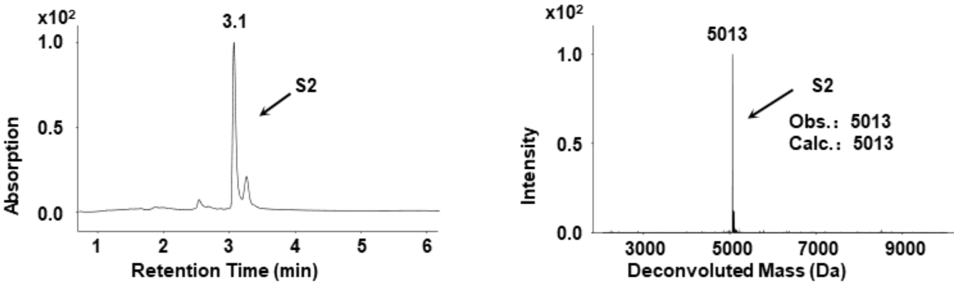

Figure S5. On-DNA reductive amination

6. Scale-up of the reaction

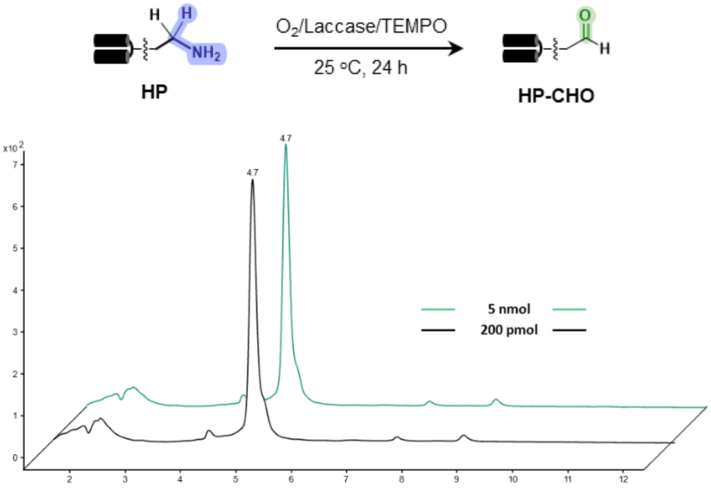

Figure S6. UPLC chromatogram of **HP** at 200 pmol and 5 nmol scales.

DNA HP (5 nmol, 1 equiv.) was mixed with CH_3COONa buffer (16 µL, 200 mM, pH 5.5), Laccase (2 µL, 0.1 U/µL in H_2O), and TEMPO (2 µL, 400 mM in 1,4-dioxane, 800 nmol, 4000 equiv.). The reaction mixture was vortexed, centrifuged, and incubated at 25 °C for 24 hours. The product was purified by ethanol precipitation and immediately analyzed using UPLC-MS, with a conversion rate exceeding 90%. Deconvoluted molecular mass: calculated 4936 Da, observed 4936 Da.

We further scaled up the HP reaction to 1 µmol, and the reaction volume was increased to 1 mL to facilitate the generation of the DNA aldehyde coupler in a single reaction. The by-products increased further with increasing reaction time. The changes in reaction time and yield are shown in Table S2 and Figure S7.

Table S2. Scale-up reaction of **HP**

Times	conversion
48 h	75.7%
52 h	77.7%
64 h	87.0%
68 h	87.0%
72 h	87.0%

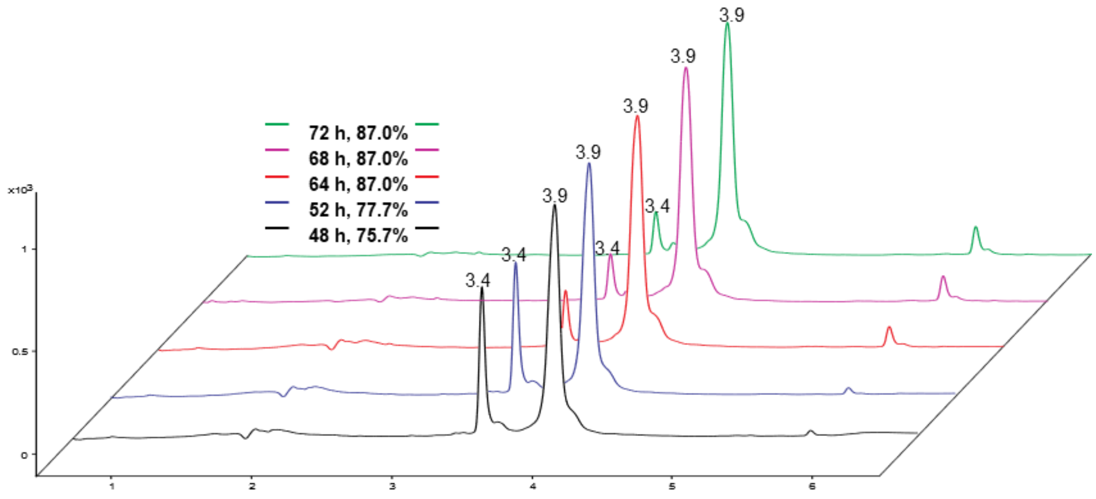

Figure S7. UPLC chromatogram of **HP** at 1 µmol scales.

7. Oxidation of three different DNA formats

7.1 Primary amine group at the 5'-terminus

5'-NH_2-DNA: 5'-NH_2 -TGACTCCCGACCGAAGGTTG-3'

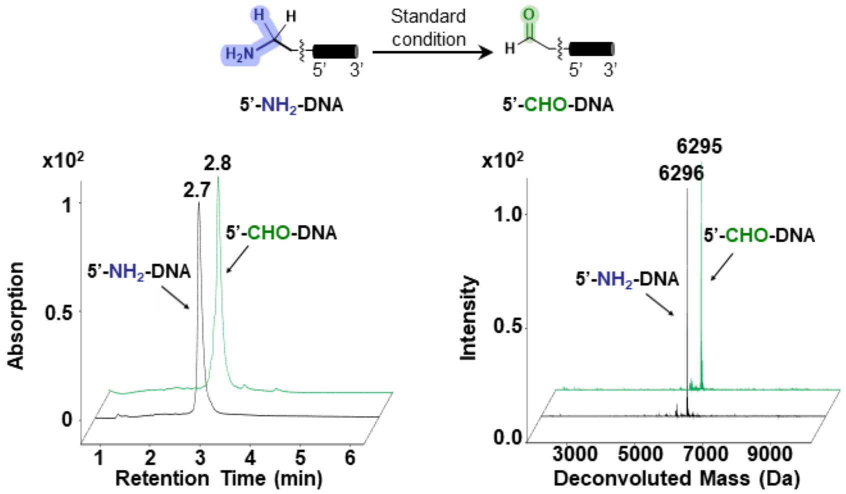

Figure S8. Amino oxidation to aldehydes at 5'-terminus

7.2 Primary amine group at the 3'-terminus

3'-NH_2-DNA: 5'-ACCTTCGGTCGGGAGTCA-NH_2-3'

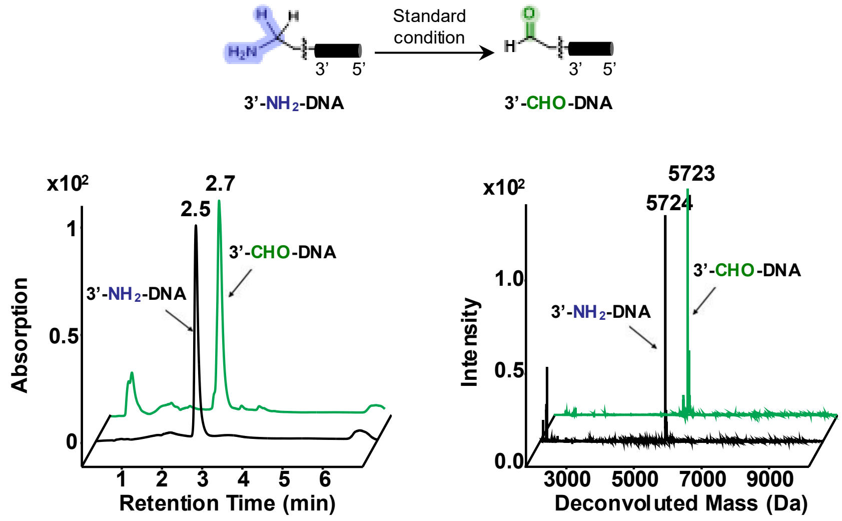

Figure S9. Amino oxidation to aldehydes at the 3'-terminus

7.3 Primary amine group at the internal site

Mid-NH_2-DNA: 5'-ACCTTCGG/iNH_2C6dT/CGGGAGTCA-3'

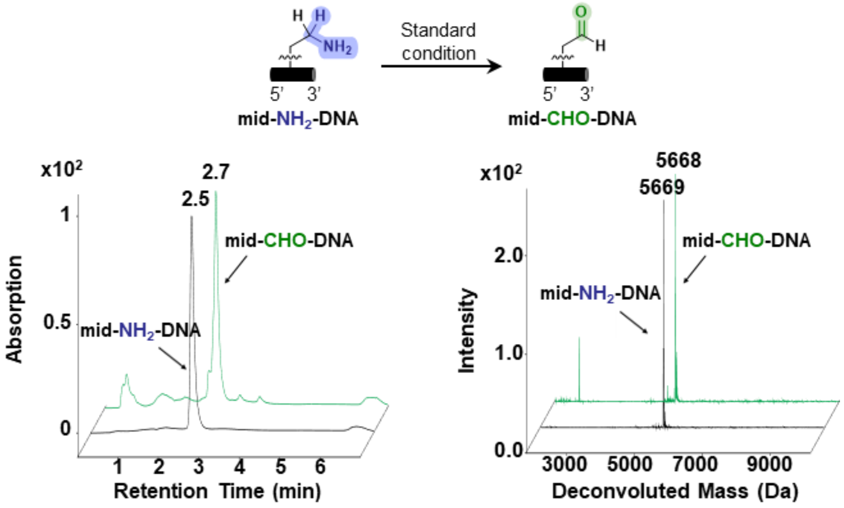

Figure S10. Amino oxidation to aldehydes at the internal site

8. Stability test of DNA-conjugated aldehydes

8.1 Chemical stability of DNA-conjugated aldehydes under storage conditions

To the DNA sample solution (1 nmol), 50 µL of H_2O was added. The mixture was subjected to freeze-thaw cycles between -80 °C and 25 °C, and aliquots were collected after 3, 5, 10, and 20 cycles for analysis. A 15 µL portion of the reaction mixture was analyzed by UPLC-MS.

DNA chemical stability during storage: The DNA sample was subjected to multiple freeze-thaw cycles (between -80 ℃ and 25 ℃).

	3 cycles	5 cycles	10 cycles	20 cycles
**DNA-conjugated amine**	√	√	√	√
**DNA-conjugated aryl aldehyde synthesized via amidation**	√	√	√	√
**DNA-conjugated aryl aldehyde synthesized via oxidation**	√	√	√	√
**DNA-conjugated alkyl aldehyde synthesized via oxidation**	√	√	√	√

√ : No degradation was detected.

Figure S11. Chemical stability of DNA-conjugated aldehydes under storage conditions

8.2 Chemical stability of DNA-conjugated aldehydes under physiological conditions

To the solution of DNA sample (1 nmol) was added 100 µL buffer. The reaction mixture was vortexed, centrifuged, and incubated at 37 °CA. At designated time points (30 min, 2 h, 4 h, 12 h, 24 h, and 48 h), 15 µL reaction mixture was obtained by ethanol precipitation and analyzed by UPLC-MS.

Buffer preparation: DMEM+10% FBS was prepared by commercial 45 mL DMEM (BasalMedia, product number: L110KJ) and 5 mL Fetal Bovine Serum (ExCell, product number: FSP500). 0.1 M pH 7.4 PBS was prepared by commercial buffer (Gibco, product number: C10010500BT). pH 7.4 Tris-HCl buffer. 0.1 M pH 7.4 Tris-HCl buffer solution was prepared by dissolving 12.114 g of Tris(hydroxymethyl)aminomethane, in approximately 800 mL of H_2O. The pH was precisely adjusted to 7. through controlled addition of concentrated hydrochloric acid. The solution was then brought to a final volume of 1 L with ultrapure water and mixed thoroughly.

(a) DNA-conjugated amine

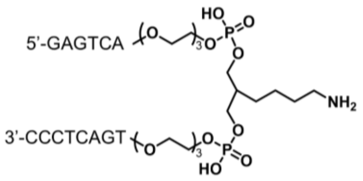

Buffer	30 min	2 h	4 h	12 h	24 h	48 h
DMEM+10% FBS	64%	■	/	/	/	/
pH 7.4 PBS	√	√	√	√	√	√
pH 7.4 Tris-HCl buffer	√	√	√	√	√	√

(b) DNA-conjugated aryl aldehyde synthesized via amidation

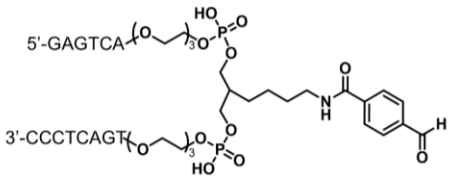

Buffer	30 min	2 h	4 h	12 h	24 h	48 h
DMEM+10% FBS	60%	■	/	/	/	/
pH 7.4 PBS	√	√	√	√	√	√
pH 7.4 Tris-HCl buffer	√	√	√	√	√	√

(c) DNA-conjugated aryl aldehyde synthesized via oxidation

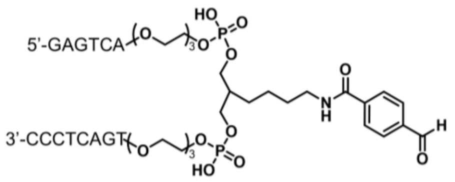

Buffer	30 min	2 h	4 h	12 h	24 h	48 h
DMEM+10% FBS	59%	■	/	/	/	/
pH 7.4 PBS	√	√	√	√	√	√
pH 7.4 Tris-HCl buffer	√	√	√	√	√	√

(d) DNA-conjugated alkyl aldehyde synthesized via oxidation

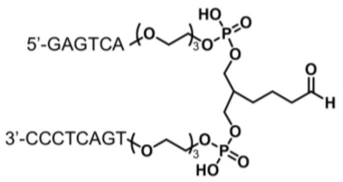

Buffer	30 min	2 h	4 h	12 h	24 h	48 h
DMEM+10% FBS	64%	■	/	/	/	/
pH 7.4 PBS	√	√	√	√	√	√
pH 7.4 Tris-HCl buffer	√	√	√	√	√	√

√ : No degradation was detected.
: A byproduct with a molecular weight 291 Da lower than the starting material was observed, corresponding to the loss of deoxythymidine monophosphate.
■: No starting material was detected.
/ : Data collection was discontinued upon observing complete damage.

Figure S12. Chemical stability of DNA-conjugated aldehydes under physiological conditions

8.3 Chemical stability of DNA-conjugated aldehydes in different buffers

To the solution of DNA sample (1 nmol) was added 100 µL buffer. The reaction mixture was vortexed, centrifuged, and incubated at 25 °C . At designated time points (30 min, 2 h, 4 h, 12 h, 24 h, and 48 h), 15 µL reaction mixture was obtained by ethanol precipitation and analyzed by UPLC-MS.

Buffer preparation: Phosphate buffers (PB, 0.2 M) at various pH values were prepared from stock solutions of 0.2 M NaH_2PO_4 and 0.2 M Na_2HPO_4 as follows: pH 5.5 PB was prepared by mixing 93.5 mL of 0.2 M NaH_2PO_4 with 6.5 mL of 0.2 M Na_2HPO_4. pH 7.4 PB was prepared by mixing 81 mL of 0.2 M NaH_2PO_4 with 81 mL of 0.2 M Na_2HPO_4. pH 9.0 PB prepared by mixing 5.3 mL of 0.2 M NaH_2PO_4 with 94.7 mL of 0.2 M Na_2HPO_4. For 0.25 M pH 9.4 borate buffered saline buffer, 23.8 g of sodium borate decahydrate ($Na_2B_4O_7 \cdot 10\ H_2O$) was dissolved in 200 mL of H_2O. The pH was adjusted to 9.4 through 5 M NaOH solution, followed by dilution to a final volume of 250 mL with H_2O. T4 DNA ligation buffer was prepared by diluting the commercial 10× buffer for T4 DNA ligase with 10 mM ATP (New England Biolabs, product number: B0202S) ten-fold with H_2O to achieve the desired 1× concentration

(a) DNA-conjugated amine

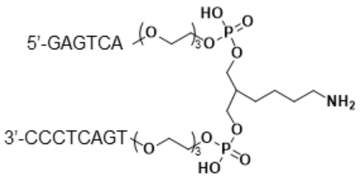

Buffer	30 min	2 h	4 h	12 h	24 h	48 h
pH 5.5 PB	√	√	√	√	√	√
pH 7.4 PB	√	√	√	√	√	√
pH 9.0 PB	√	√	√	√	√	√
pH 9.4 BBS	√	√	√	√	√	√
T4 DNA ligation buffer	√	√	√	√	√	√

(b) DNA-conjugated aryl aldehyde synthesized via amidation

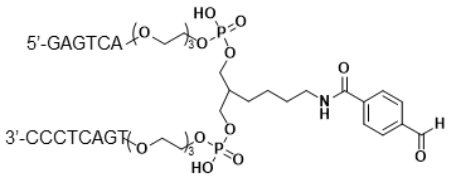

Buffer	30 min	2 h	4 h	12 h	24 h	48 h
pH 5.5 PB	√	√	√	√	√	√
pH 7.4 PB	√	√	√	√	√	√
pH 9.0 PB	√	√	√	√	√	√
pH 9.4 BBS	√	√	√	√	√	√
T4 DNA ligation buffer	√	√	√	√	√	√

(c) DNA-conjugated aryl aldehyde synthesized via oxidation

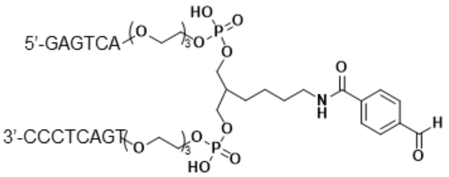

buffer	30 min	2 h	4 h	12 h	24 h	48 h
pH 5.5 PB	√	√	√	√	√	√
pH 7.4 PB	√	√	√	√	√	√
pH 9.0 PB	√	√	√	√	√	√
pH 9.4 BBS	√	√	√	√	√	√
T4 DNA ligation buffer	√	√	√	√	√	√

(d) DNA-conjugated alkyl aldehyde synthesized via oxidation

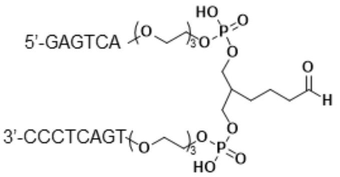

Buffer	30 min	2 h	4 h	12 h	24 h	48 h
pH 5.5 PB	√	√	√	√	√	√
pH 7.4 PB	√	√	√	√	√	√
pH 9.0 PB	√	√	√	√	√	√
pH 9.4 BBS	56%	67%	70%	73%		/
T4 DNA ligation buffer	√	√	√	√	√	√

√ : No degradation was detected.
: A +43 Da byproduct was observed, presumably arising from boronic acid addition followed by dehydration.
: No starting material was detected.
/ : Data collection was discontinued upon observing complete damage.

Figure S13. Stability of DNA-conjugated amine and aldehyde products under physiological and buffer conditions. (a-d) Four types of DNA conjugates were incubated at 25 °C in five different buffers: 0.2 M PB (pH 5.5), 0.2 M PB (pH 7.4), 0.2 M PB (pH 9.0), 0.25 M BBS (pH 9.4) and 1× T4 DNA ligation buffer.

8.4 Chemical stability of DNA-conjugated aldehydes at different temperature

To the solution of DNA sample (1 nmol) was added 100 µL H_2O. The reaction mixture was vortexed, centrifuged, and incubated at 25 °C, 37 °C, 60 °C,and 80 °C. At designated time points (30 min, 1 h, 2 h, 4 h, 6 h, 12 h, 24 h, and 48 h), a 15 µL portion of the reaction mixture was analyzed by UPLC-MS.

(a) DNA-conjugated amine

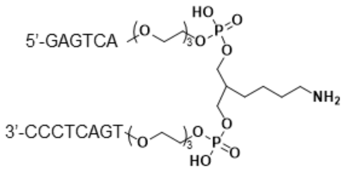

Temperature	30 min	2 h	4 h	12 h	24 h	48 h
25 °C	√	√	√	√	√	√
37 °C	√	√	√	√	√	√
60 °C	√	√	√	√	√	17%
80 °C	√	√	7%	19%<br>6%	■	/

(b) DNA-conjugated aryl aldehyde synthesized via amidation

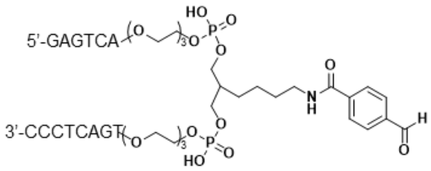

Temperature	30 min	2 h	4 h	12 h	24 h	48 h
25 °C	√	√	√	√	√	√
37 °C	√	√	√	√	√	√
60 °C	√	√	√	√	√	√
80 °C	√	√	√	7%	7%<br>13%	■

(c) DNA-conjugated aryl aldehyde synthesized via oxidation

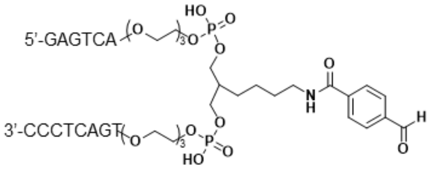

Temperature	30 min	2 h	4 h	12 h	24 h	48 h
25 °C	√	√	√	√	√	√
37 °C	√	√	√	√	√	√
60 °C	√	√	√	√	√	7%
80 °C	√	√	7%<br>7%	41%<br>16%<br>11%	■	/

(d) DNA-conjugated alkyl aldehyde synthesized via oxidation

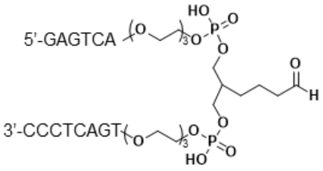

Temperature	30 min	2 h	4 h	12 h	24 h	48 h
25 °C	√	√	√	√	√	√
37 °C	√	√	√	√	√	√
60 °C	√	√	√	√	15%<br>5%	79%
80 °C	11%	23%<br>16%	42%<br>15%<br>13%	/	/	/

√ : No degradation was detected.
: A byproduct with a molecular weight 117 Da lower than the starting material was observed.
: A byproduct with a molecular weight 346 Da lower than the starting material was observed.
: A byproduct with a molecular weight 80 Da lower than the starting material was observed.
: A byproduct with a molecular weight 1879 Da lower than the starting material was observed.
: No starting material was detected.
/ : Data collection was discontinued upon observing complete damage.

Figure S14. Chemical stability of DNA-conjugated aldehydes under different temperatures

9. UPLC-MS Spectrum of different substrates

9.1 Substrate scope of on-DNA primary amine oxidation

UPLC chromatogram and deconvoluted MS of **a5**

Conversion: ＞90%

Calculated Mass: 5021 Da; Observed Mass: 5021 Da

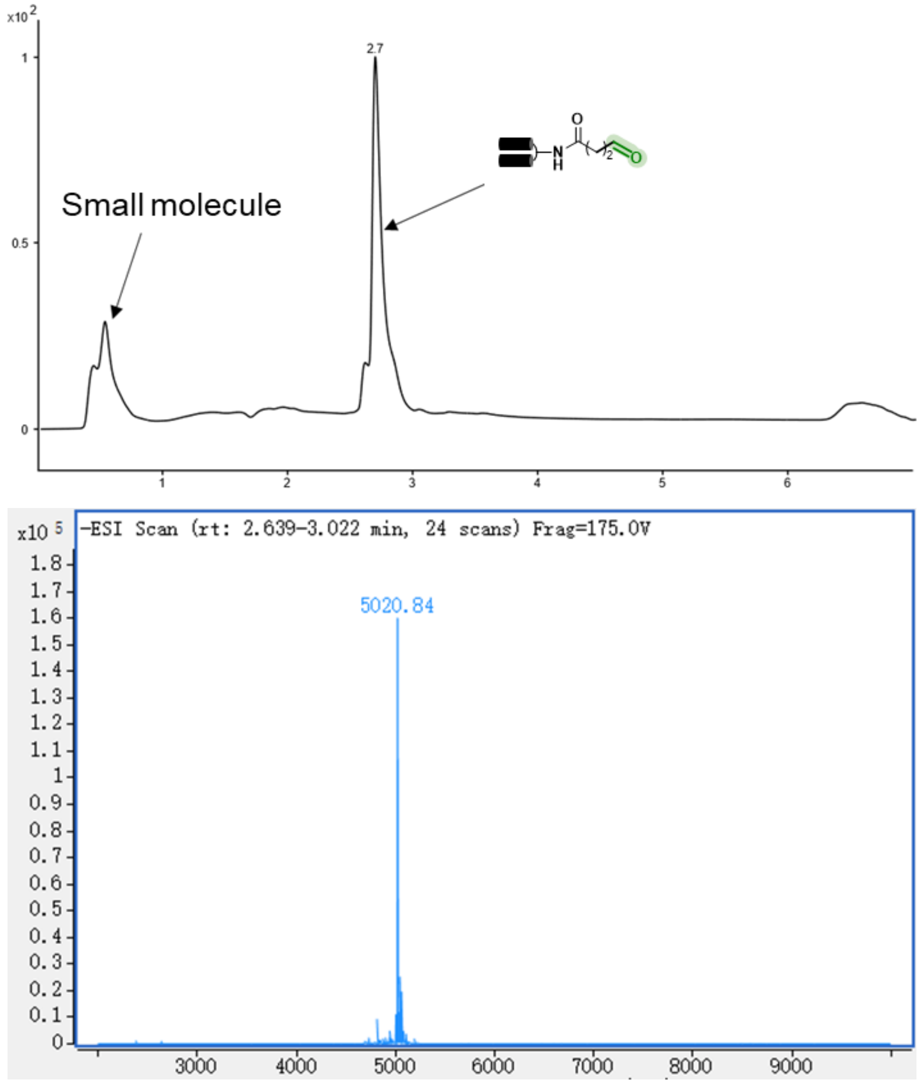

UPLC chromatogram and deconvoluted MS of **a6**

Conversion: 81%

Calculated Mass: 5035 Da; Observed Mass: 5035 Da

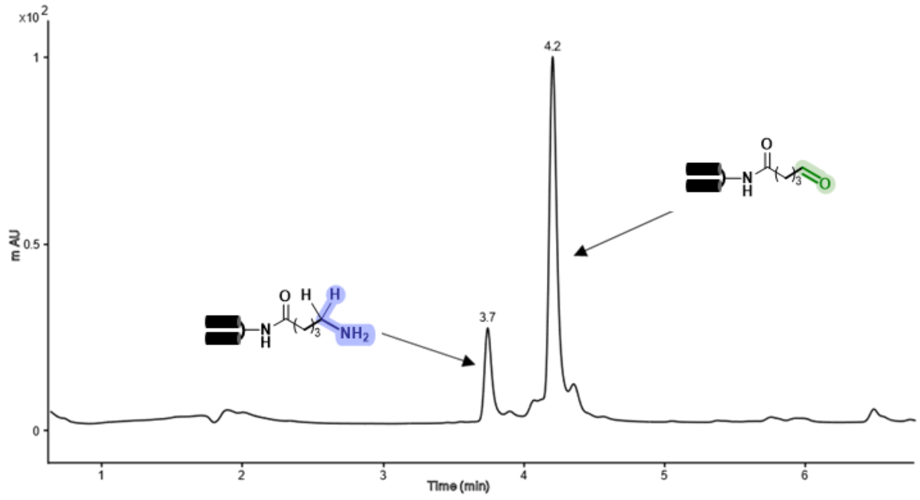

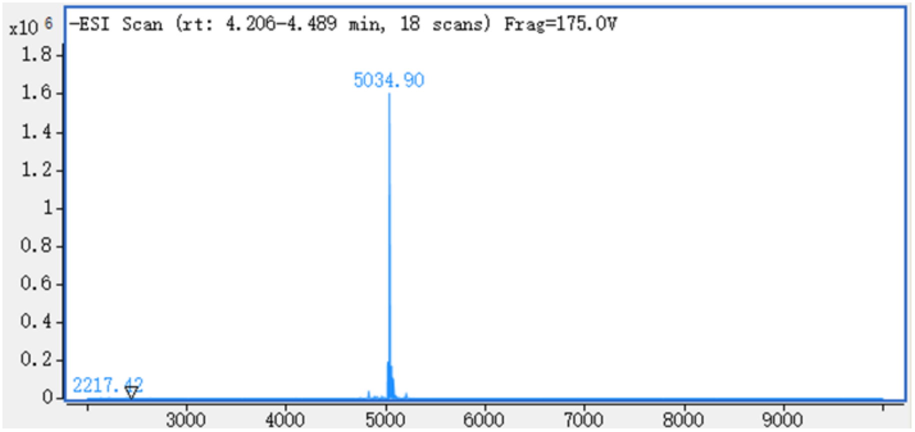

UPLC chromatogram and deconvoluted MS of **a7**

Conversion: 70%

Calculated Mass: 5077 Da; Observed Mass: 5077 Da

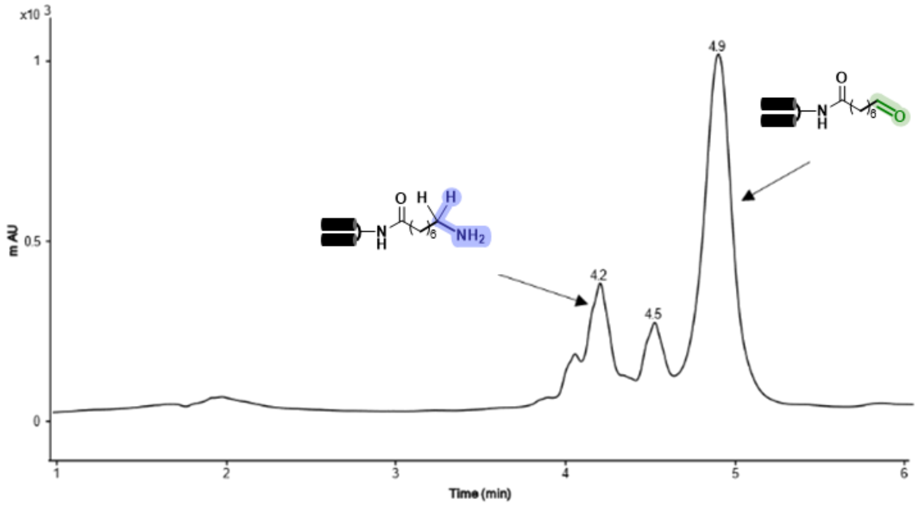

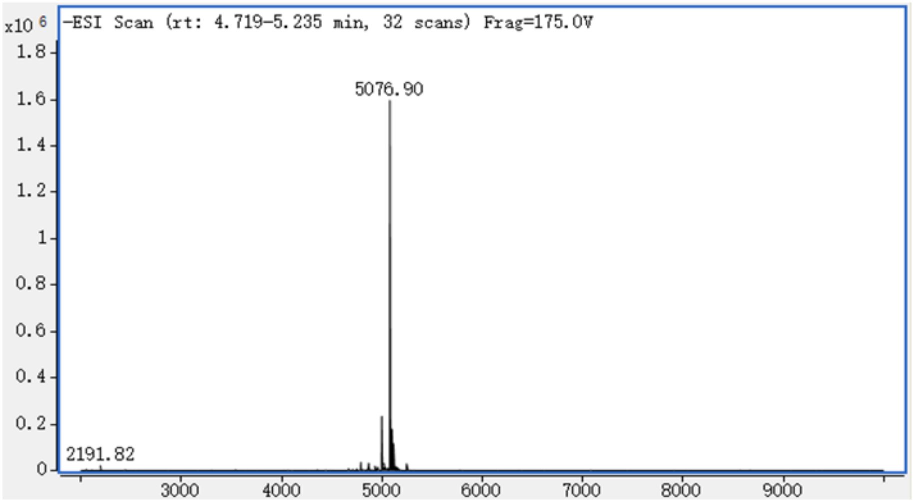

UPLC chromatogram and deconvoluted MS of **a8**

Conversion: 58%

Calculated Mass: 5133 Da; Observed Mass: 5133 Da

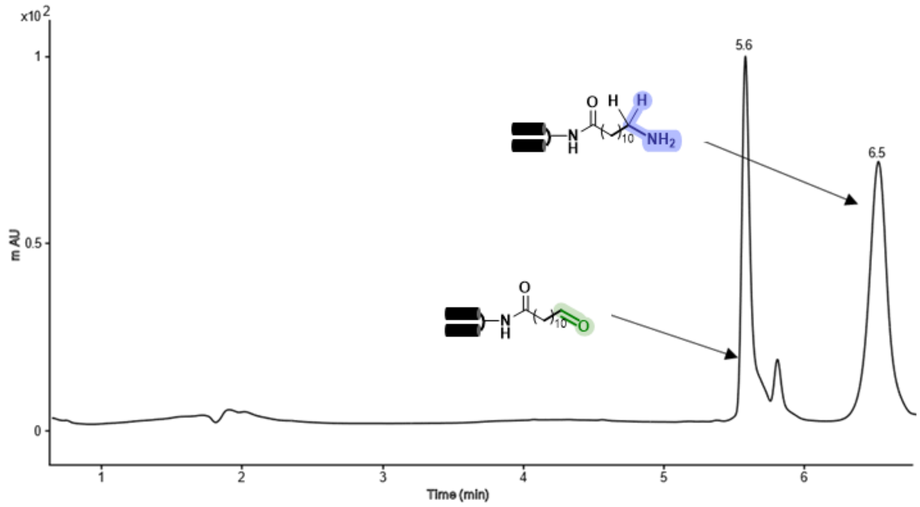

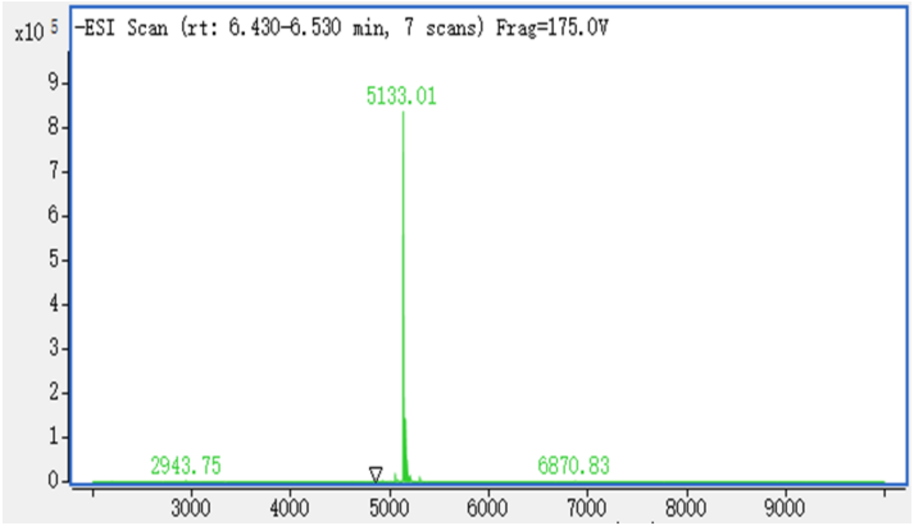

UPLC chromatogram and deconvoluted MS of **a9**

Conversion: 76%

Calculated Mass: 5198 Da; Observed Mass: 5198 Da

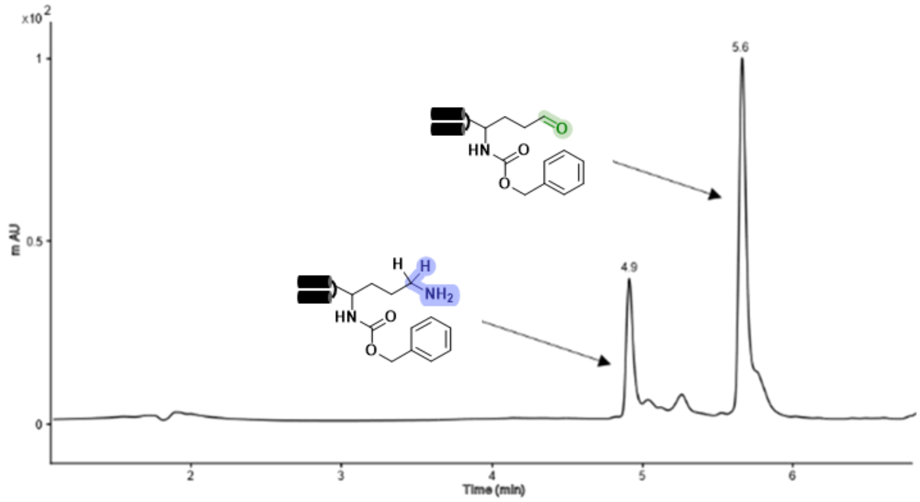

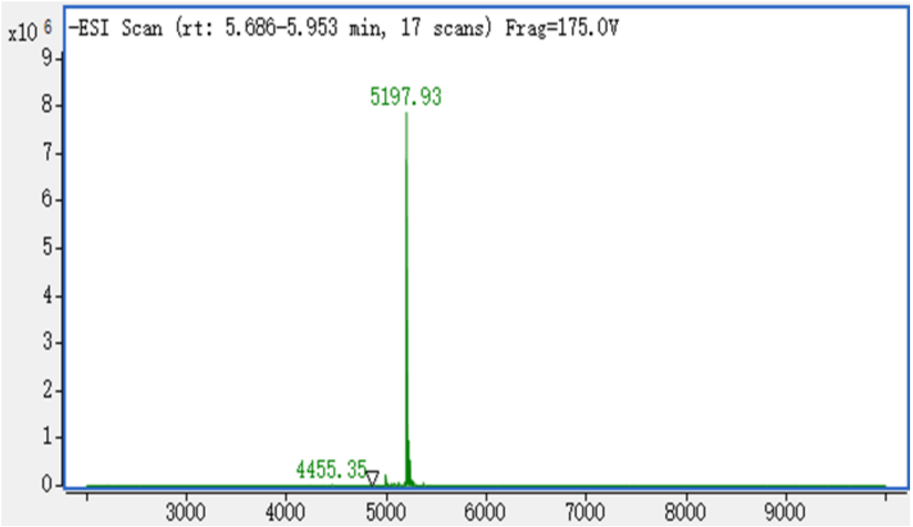

UPLC chromatogram and deconvoluted MS of **a10**

Conversion: 68%

Calculated Mass: 5148 Da; Observed Mass: 5148 Da

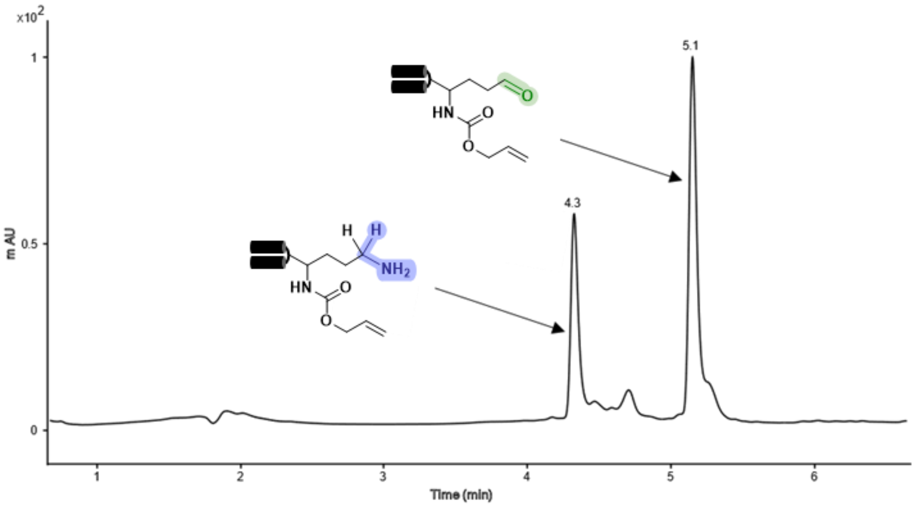

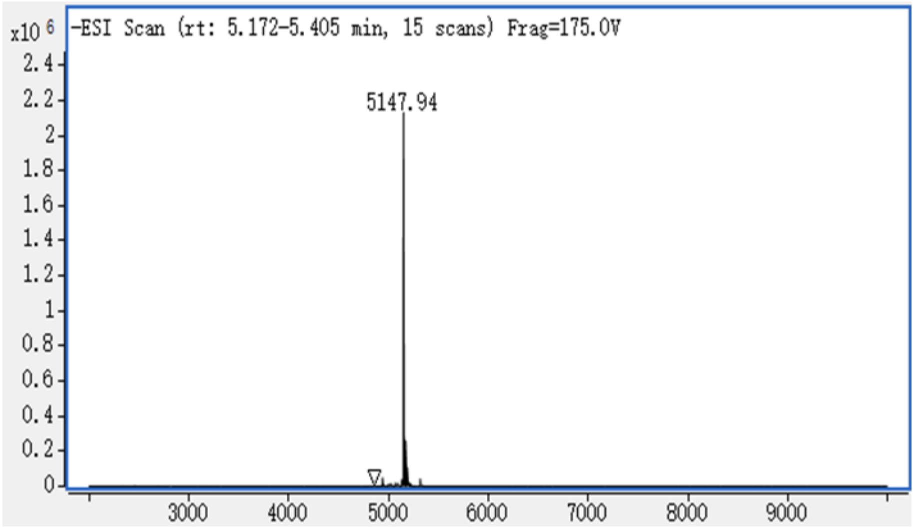

UPLC chromatogram and deconvoluted MS of **a11**

Conversion: 87%

Calculated Mass: 5075 Da; Observed Mass: 5075 Da

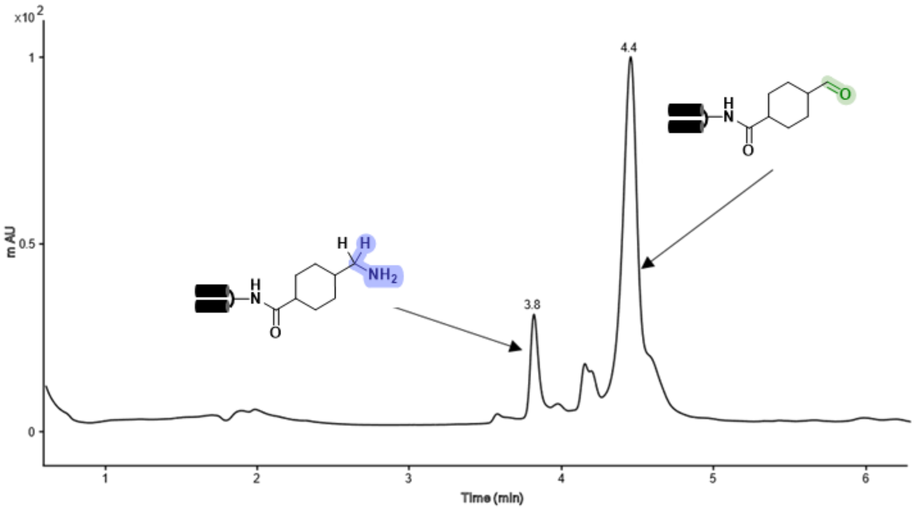

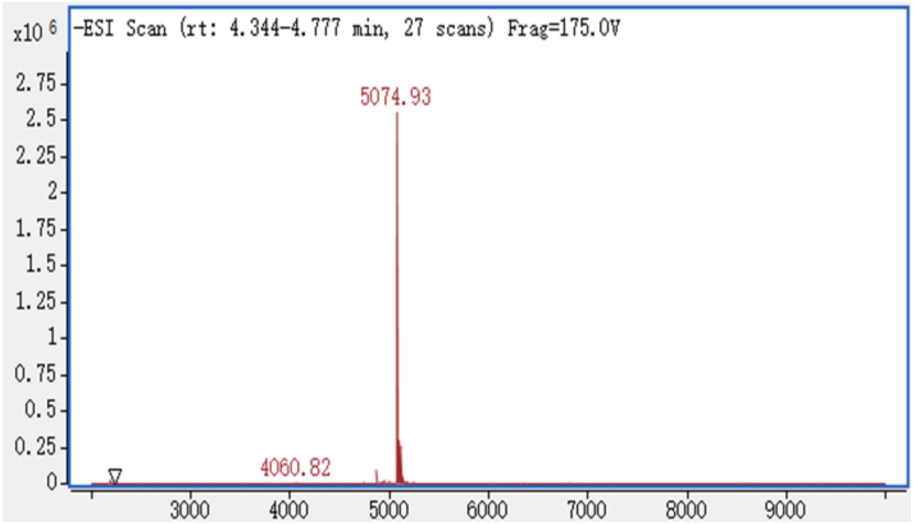

UPLC chromatogram and deconvoluted MS of **a12**

Conversion: ＞90%

Calculated Mass: 5069; served Mass: 5069 Da

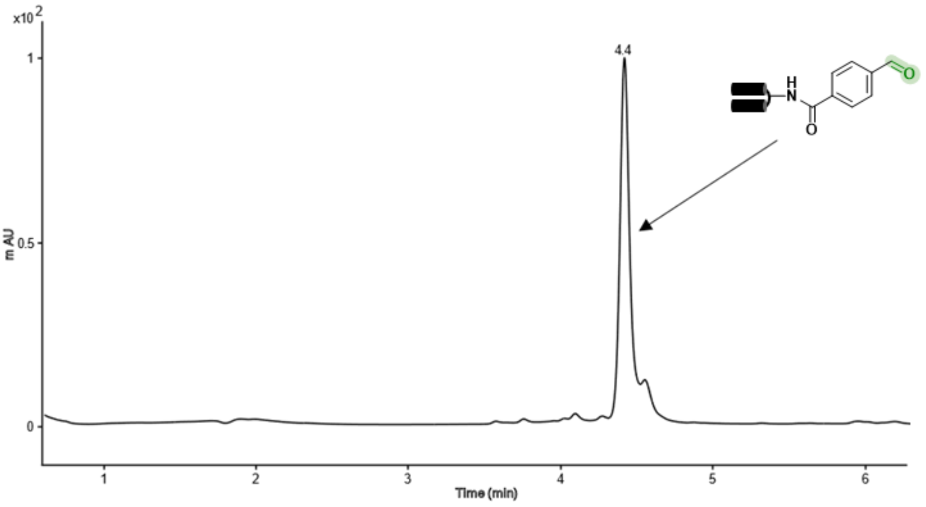

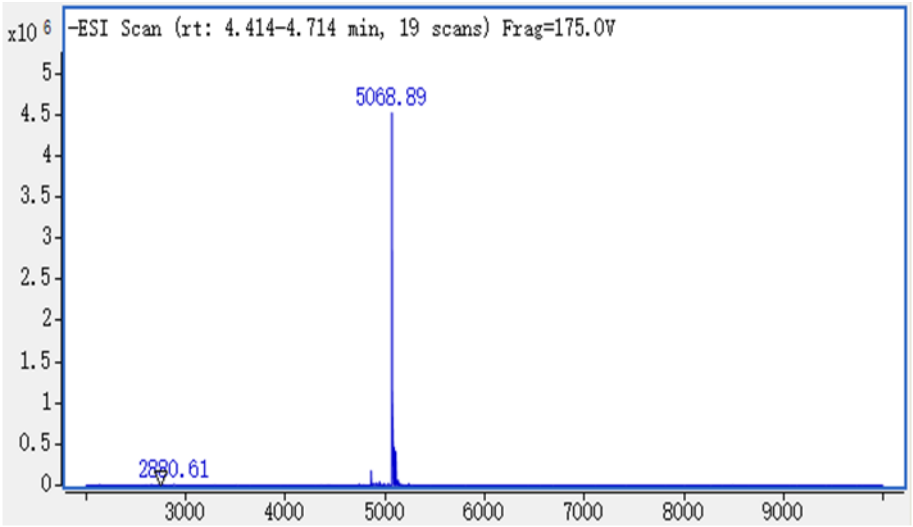

UPLC chromatogram and deconvoluted MS of **a13**

Conversion: ＞90%

Calculated Mass: 5084 Da; Observed Mass: 5084 Da

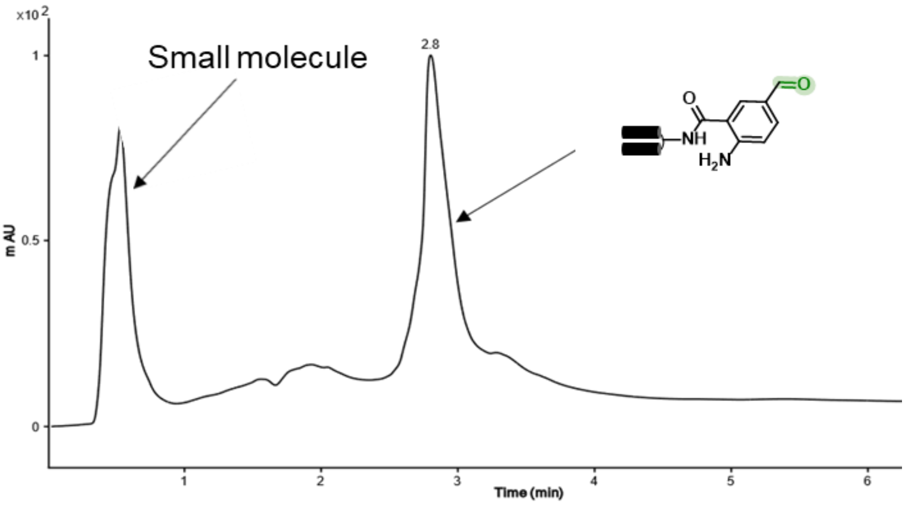

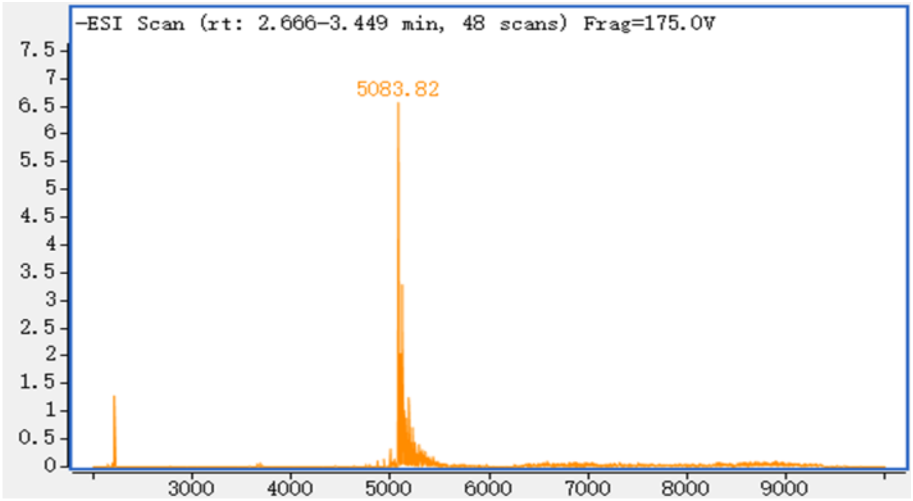

UPLC chromatogram and deconvoluted MS of **a14**

Conversion: ＞90%

Calculated Mass: 5094 Da; Observed Mass: 5094 Da

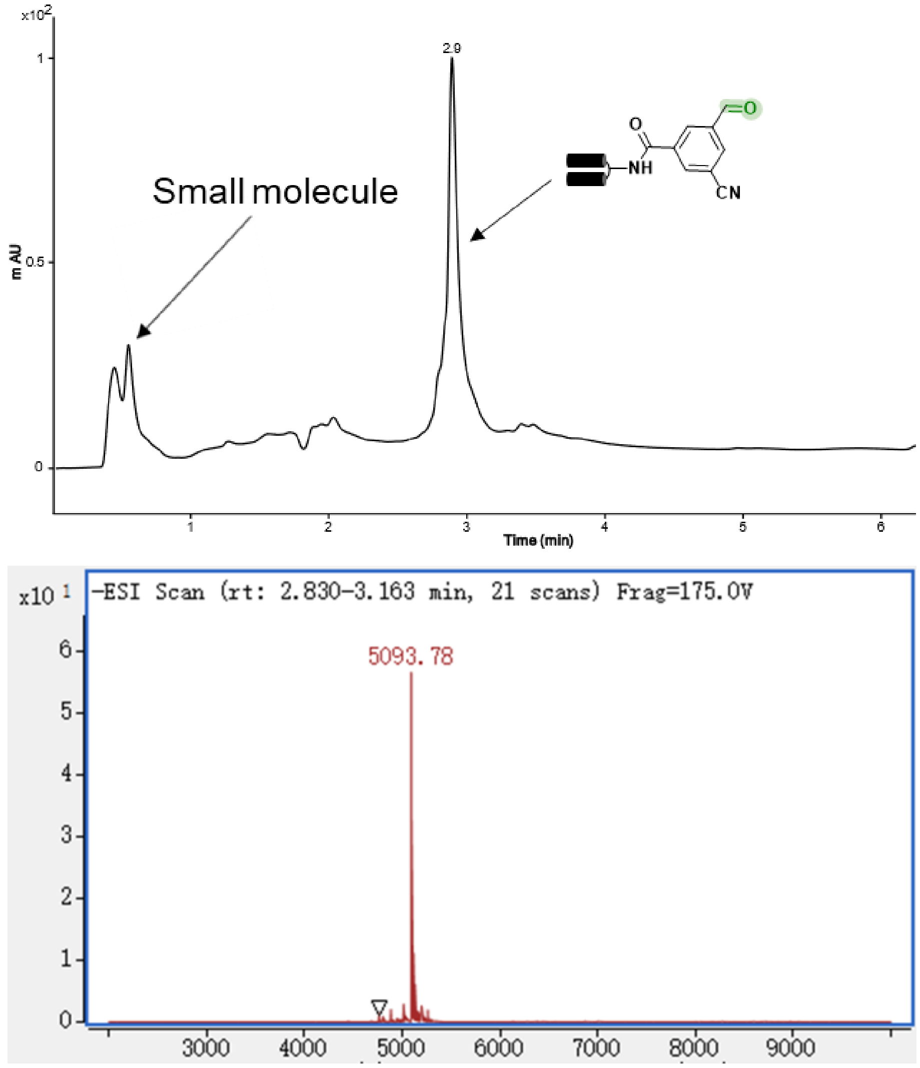

UPLC chromatogram and deconvoluted MS of **a15**

Conversion: ＞90%

Calculated Mass: 5113 Da; Observed Mass: 5113 Da

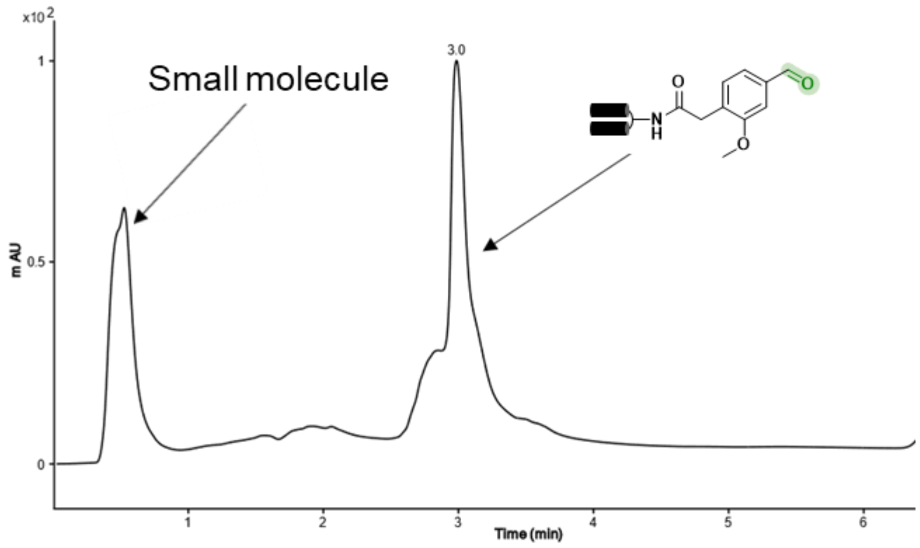

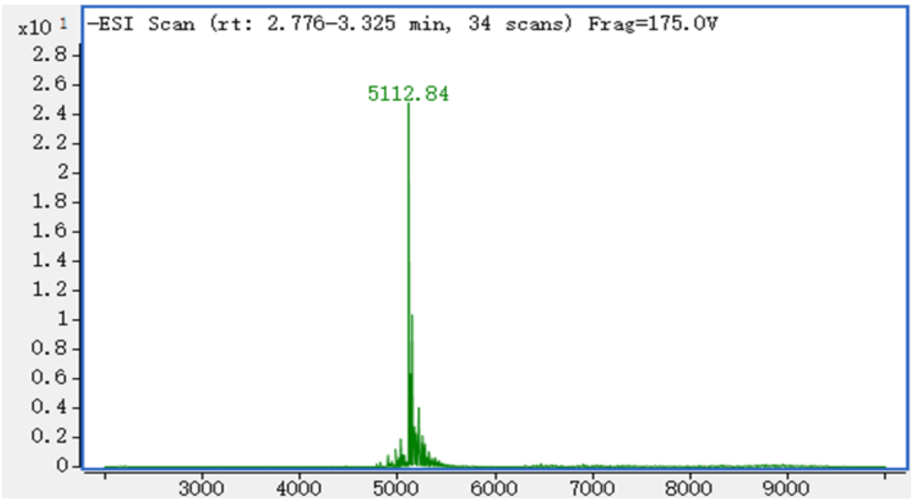

UPLC chromatogram and deconvoluted MS of **a16**

Conversion: ＞90%

Calculated Mass: 5122 Da; Observed Mass: 5122 Da

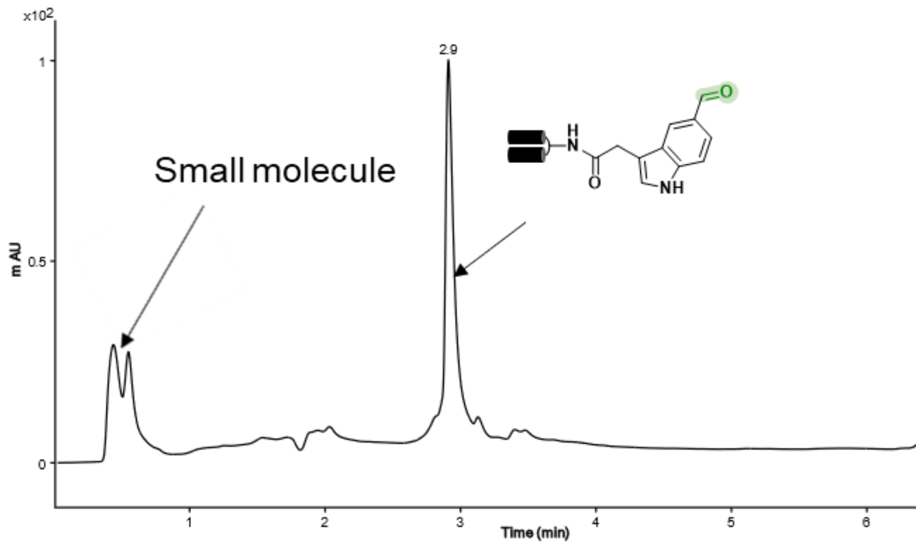

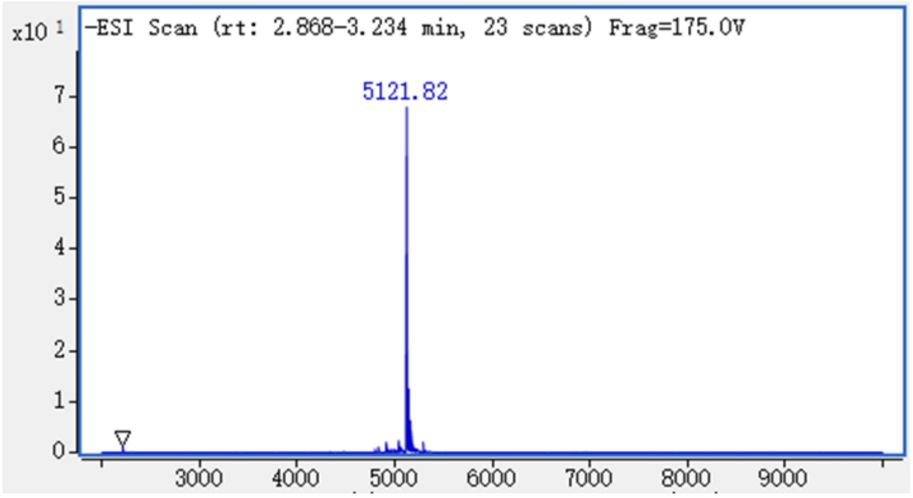

UPLC chromatogram and deconvoluted MS of **a17**

Conversion: ＞90%

Calculated Mass: 5164 Da; Observed Mass: 5164 Da

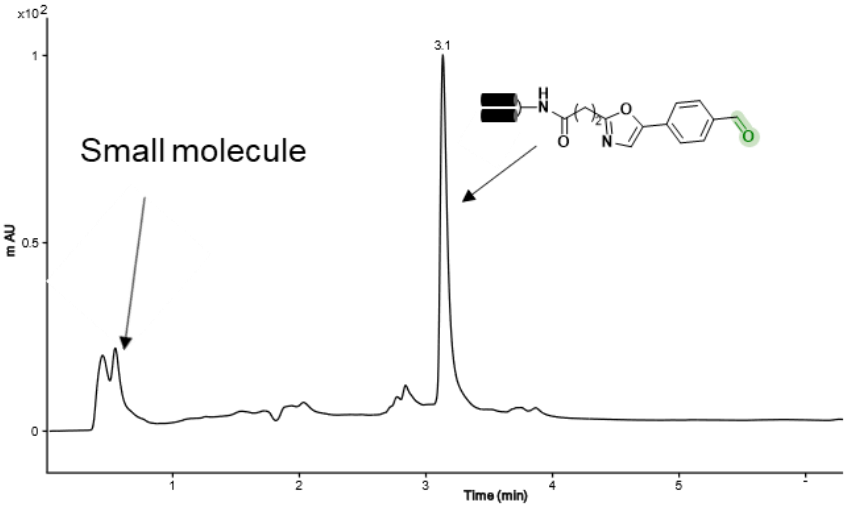

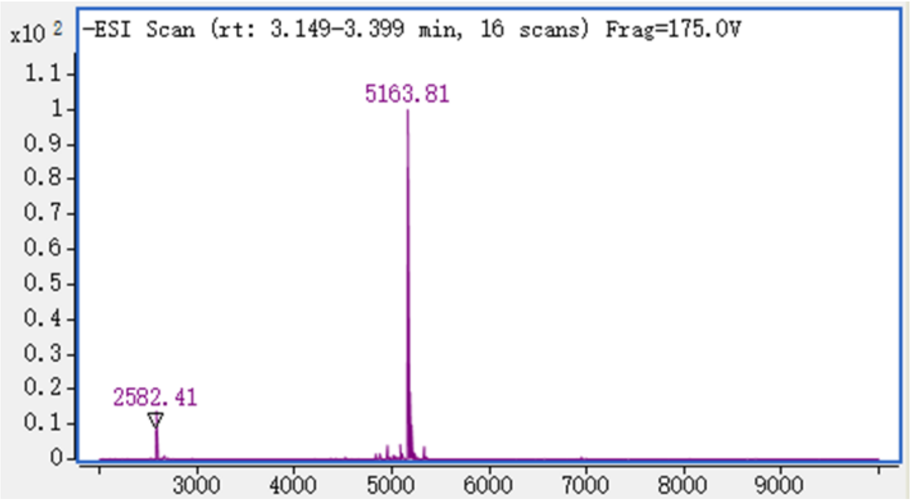

UPLC chromatogram and deconvoluted MS of **a18**

Conversion: ＞90%

Calculated Mass: 5381 Da; Observed Mass: 5381 Da

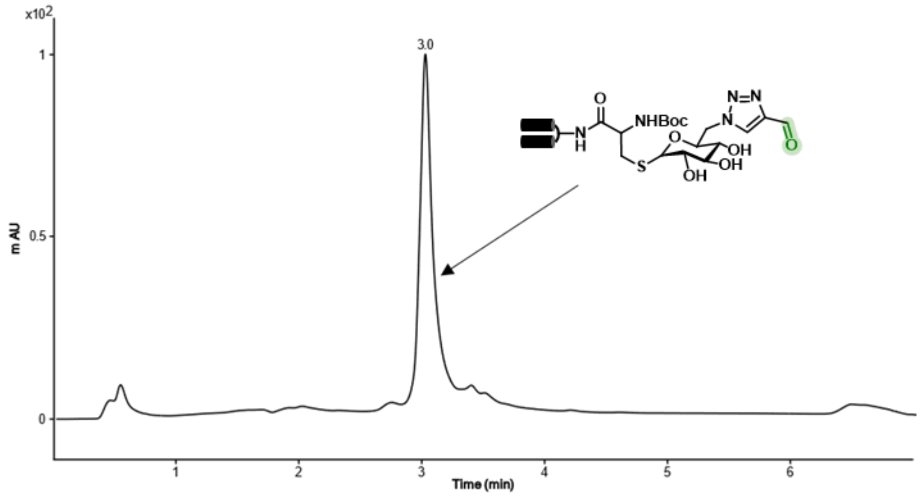

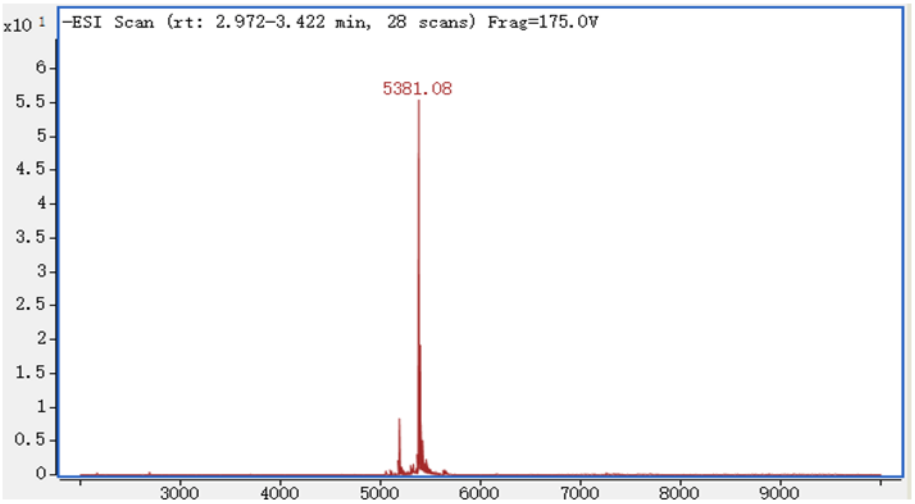

UPLC chromatogram and deconvoluted MS of **a19**

Conversion: 50%

Calculated Mass: 5035 Da; Observed Mass: 5035 Da

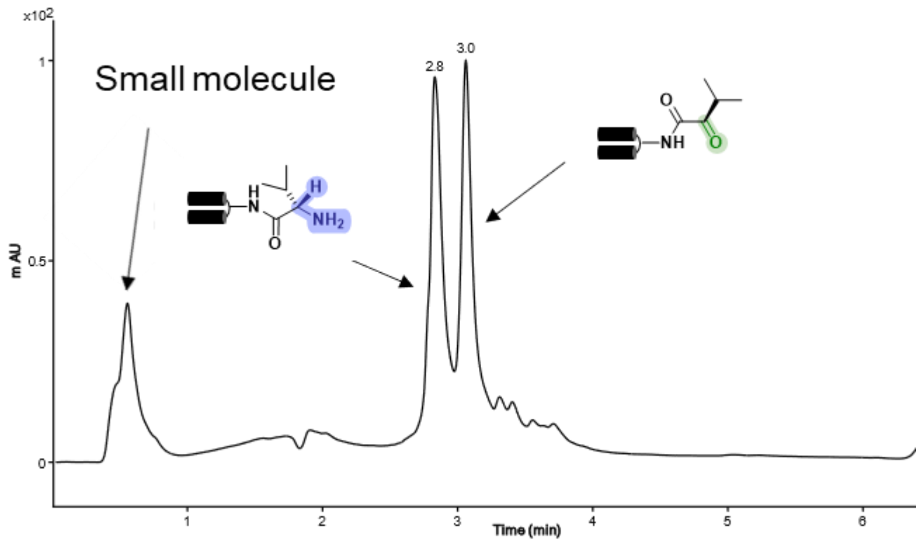

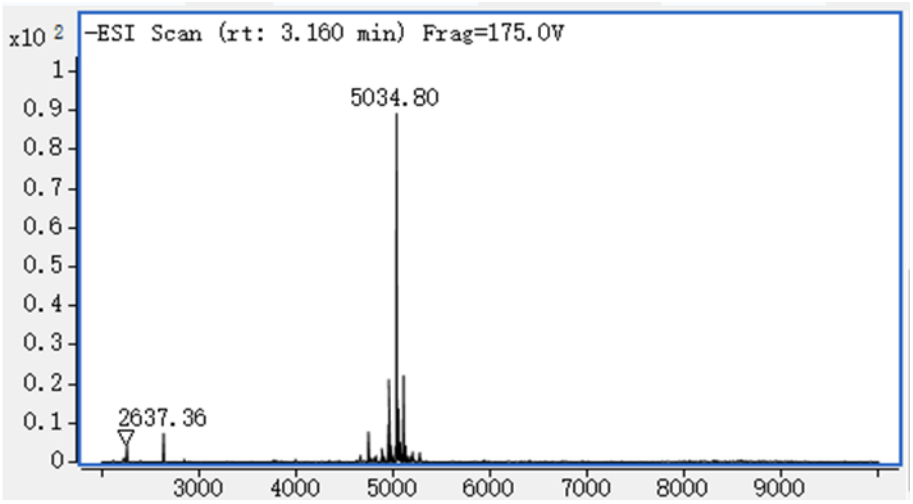

UPLC chromatogram and deconvoluted MS of **a20**

Conversion: 67%

Calculated Mass: 5075 Da; Observed Mass: 5075 Da

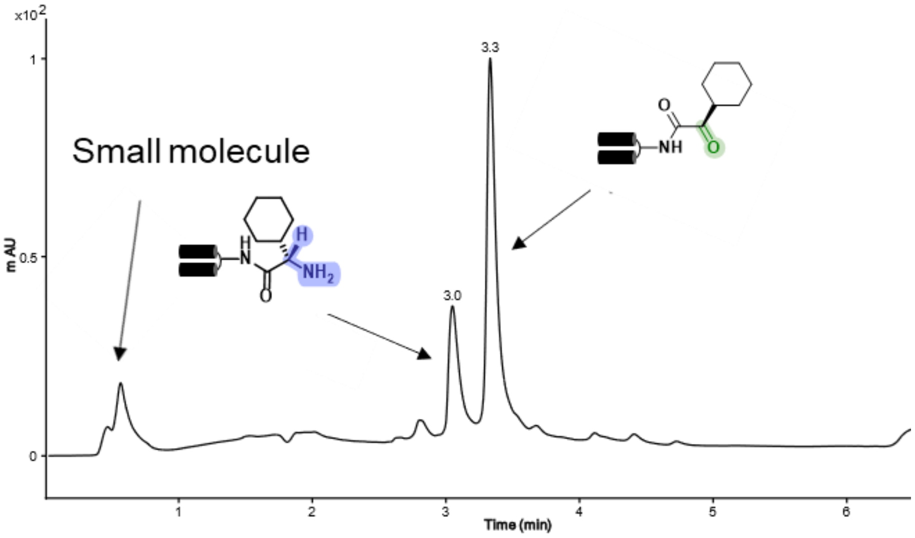

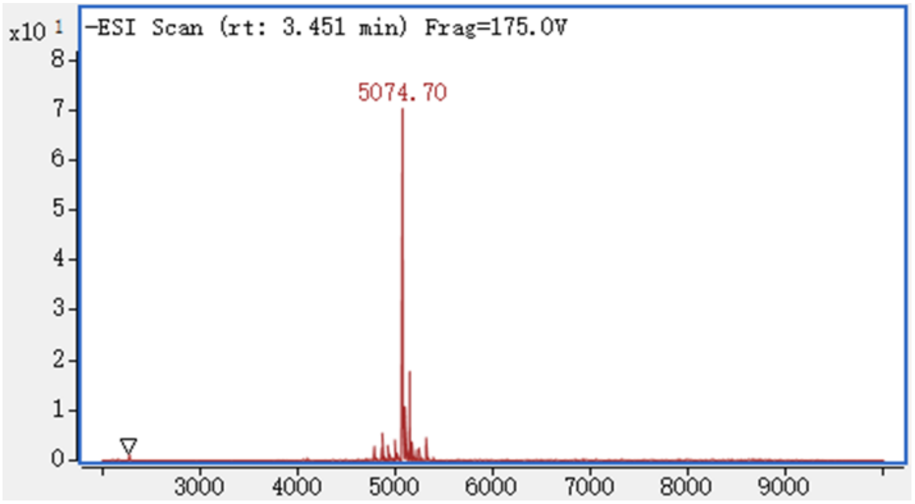

UPLC chromatogram and deconvoluted MS of **a21**

Conversion: ＞90%

Calculated Mass: 5069 Da; Observed Mass: 5069 Da

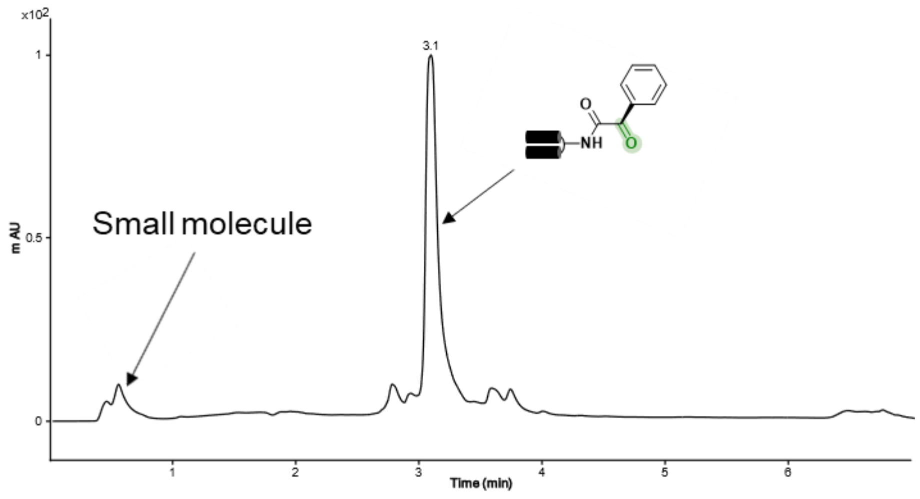

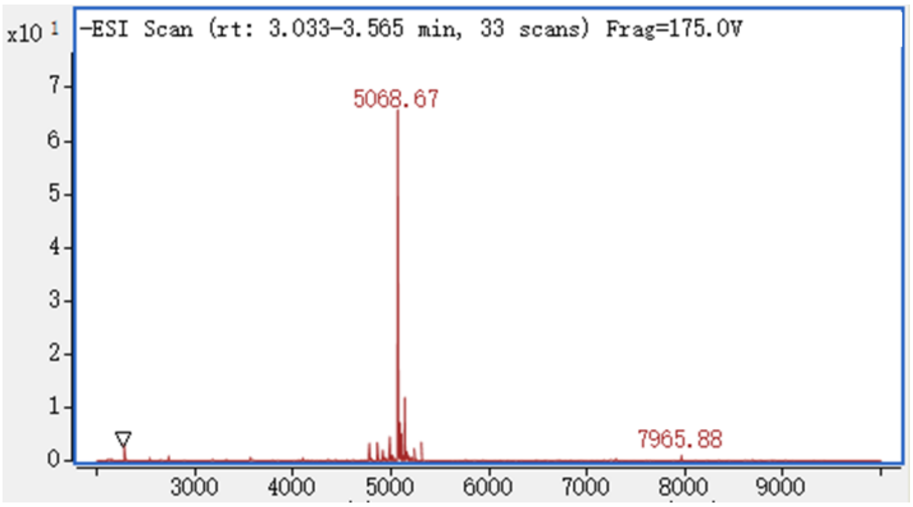

9.2 Substrate scope of cleavage and oxidation of DNA-conjugated secondary or tertiary amines

UPLC chromatogram and deconvoluted MS of **c1**

Conversion: ＞90%

Calculated Mass: 4936 Da; Observed Mass: 4936 Da

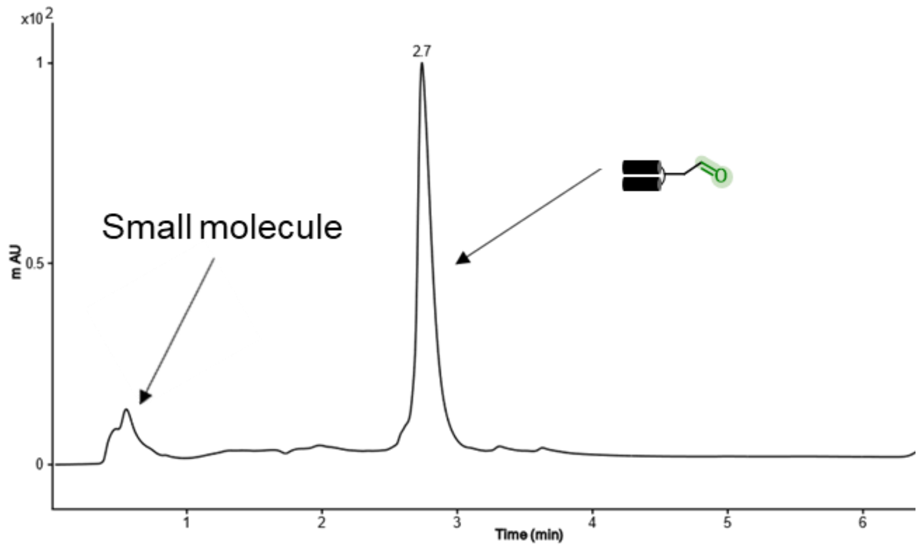

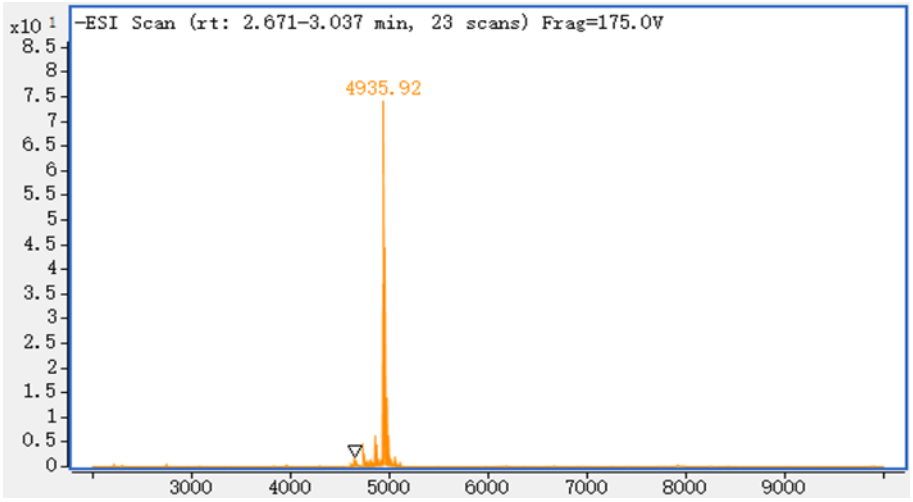

UPLC chromatogram and deconvoluted MS of **c2**

Conversion: ＞90%

Calculated Mass: 4936 Da; Observed Mass: 4936 Da

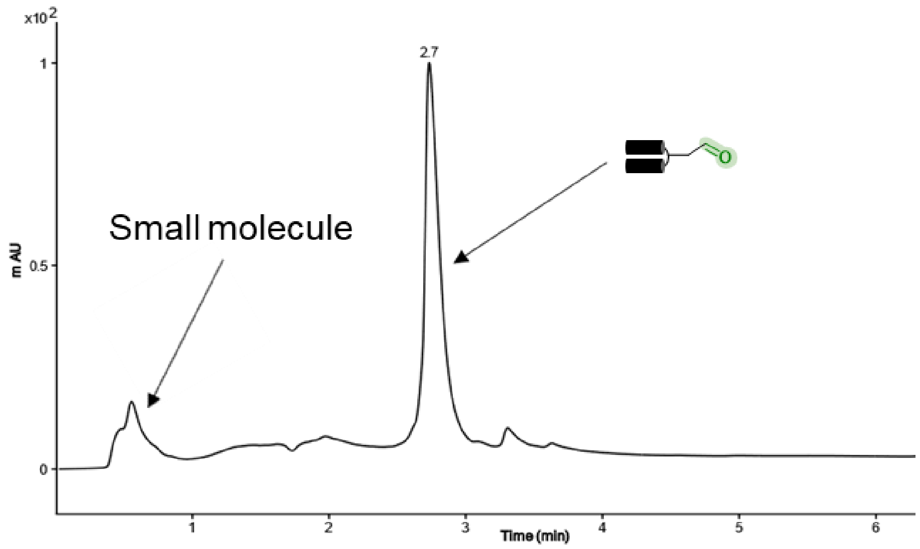

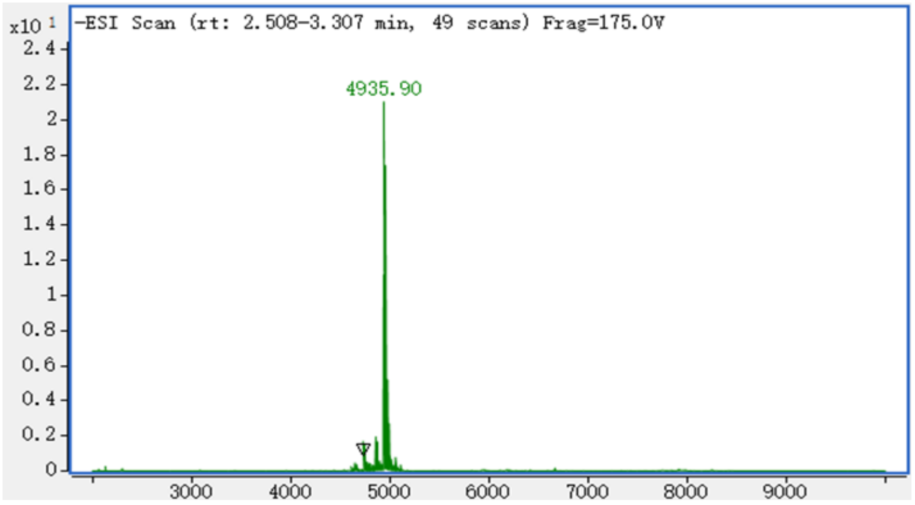

UPLC chromatogram and deconvoluted MS of **c3**

Conversion: ＞90%

Calculated Mass: 4936 Da; Observed Mass: 4936 Da

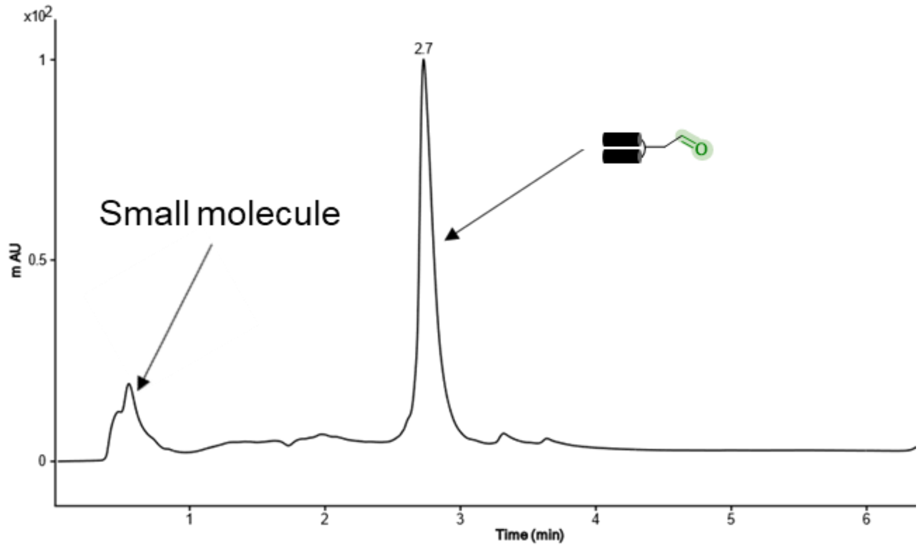

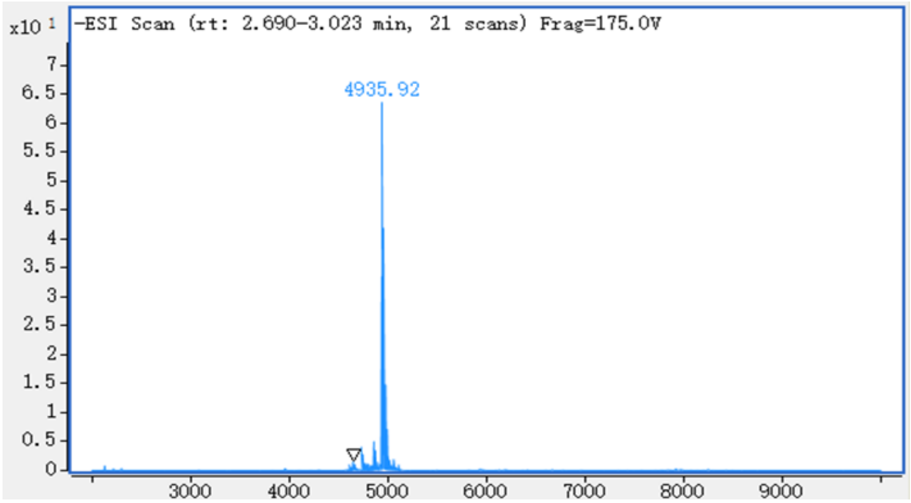

UPLC chromatogram and deconvoluted MS of **c4**

Conversion: ＞90%

Calculated Mass: 4936 Da; Observed Mass: 4936 Da

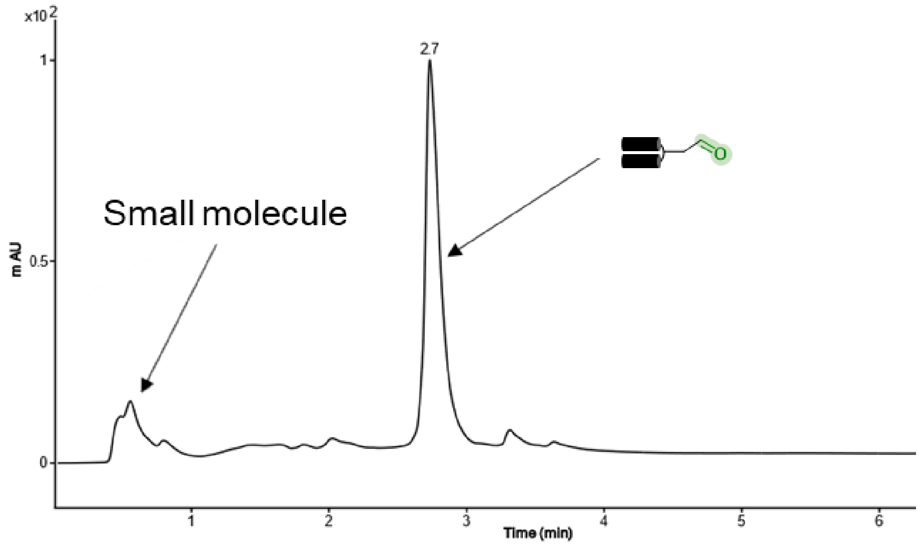

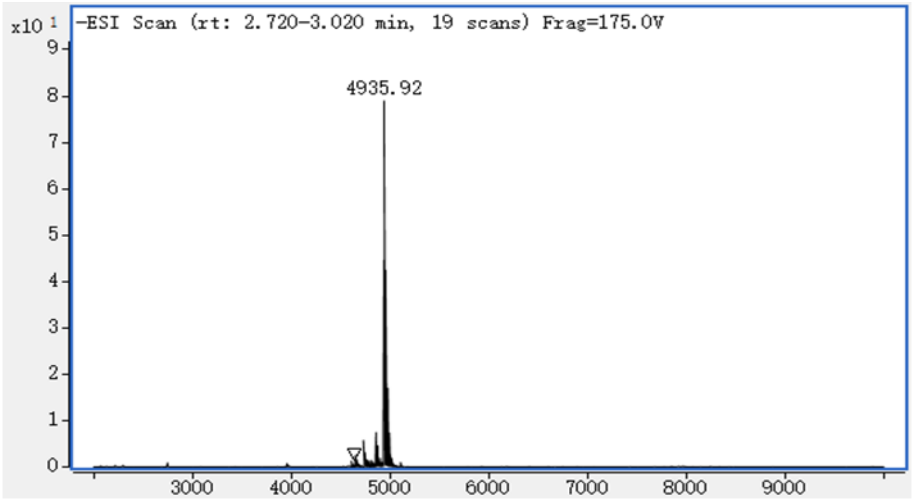

UPLC chromatogram and deconvoluted MS of c5

Conversion: 80%

Calculated Mass: 4936 Da; Observed Mass: 4936 Da

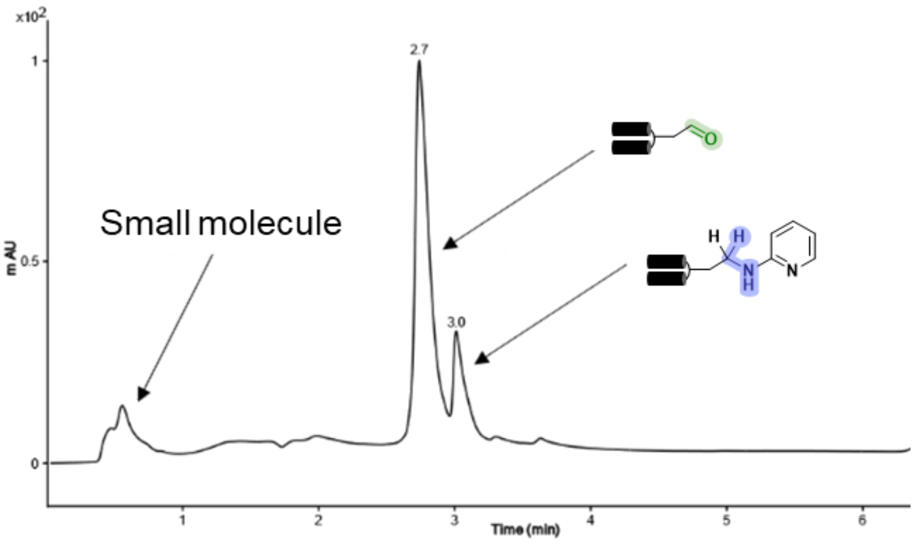

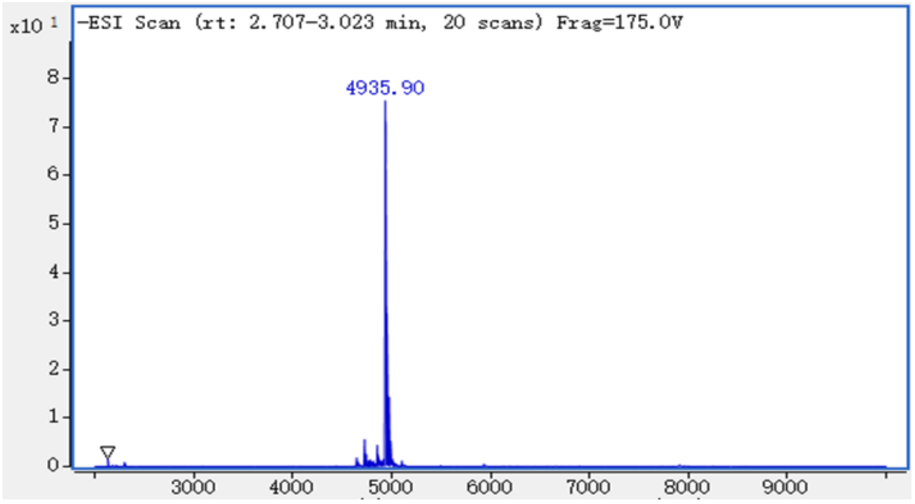

UPLC chromatogram and deconvoluted MS of **c6**

Conversion: ＞90%

Calculated Mass: 5019 Da; Observed Mass: 5019 Da

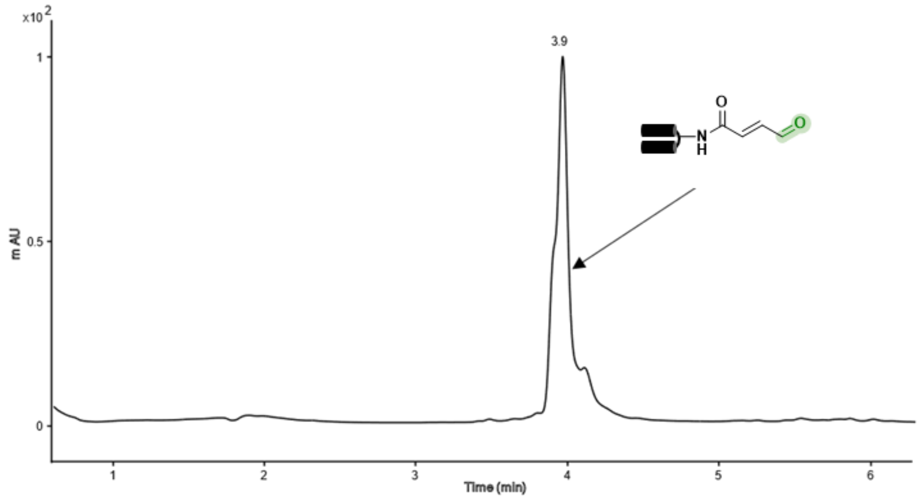

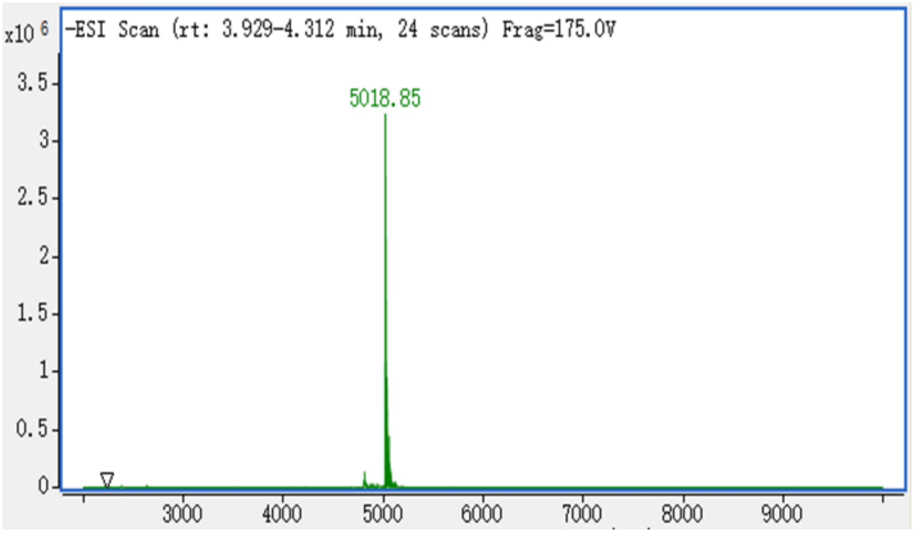

UPLC chromatogram and deconvoluted MS of **c7**

Conversion: ＞90%

Calculated Mass: 5069 Da; Observed Mass: 5069 Da

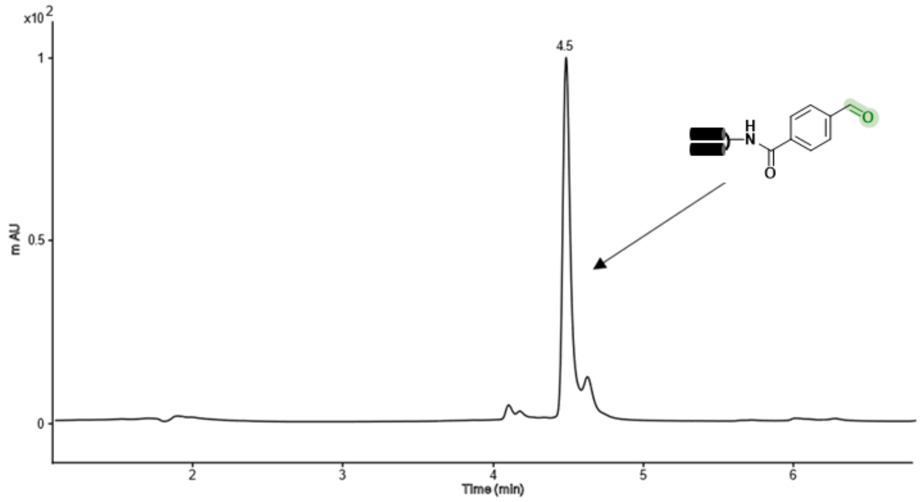

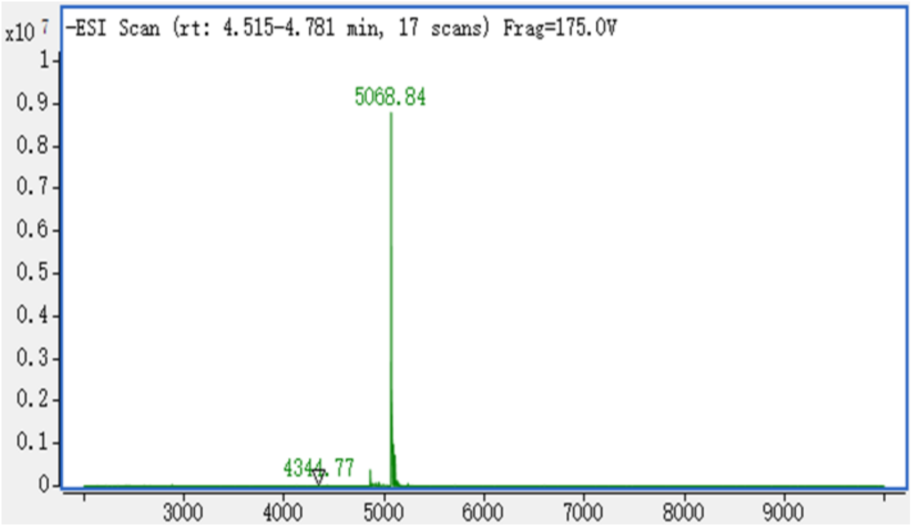

UPLC chromatogram and deconvoluted MS of **c8**

Conversion: ＞90%

Calculated Mass: 5069 Da; Observed Mass: 5069 Da

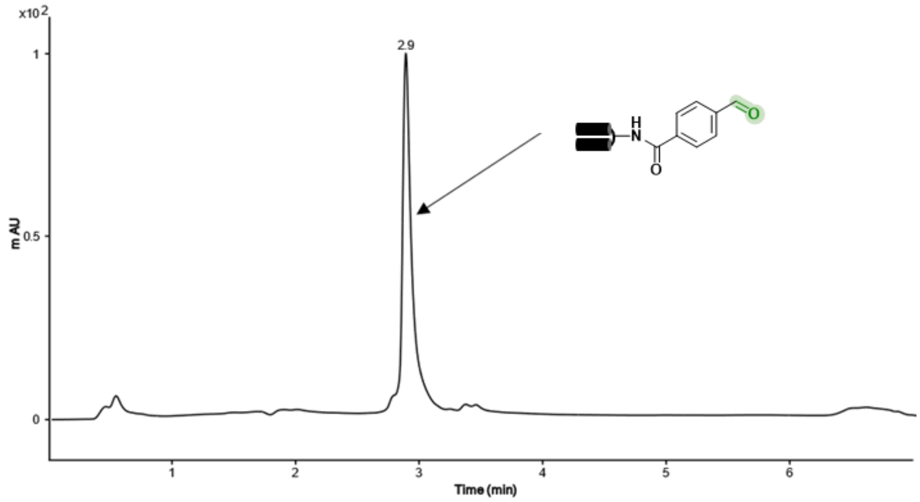

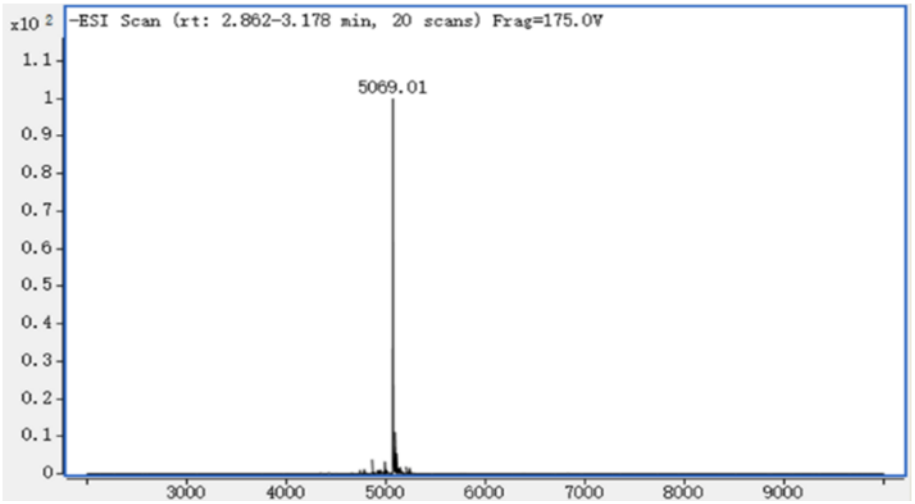

UPLC chromatogram and deconvoluted MS of **c9**

Conversion: ＞90%

Calculated Mass: 5069 Da; Observed Mass: 5069 Da

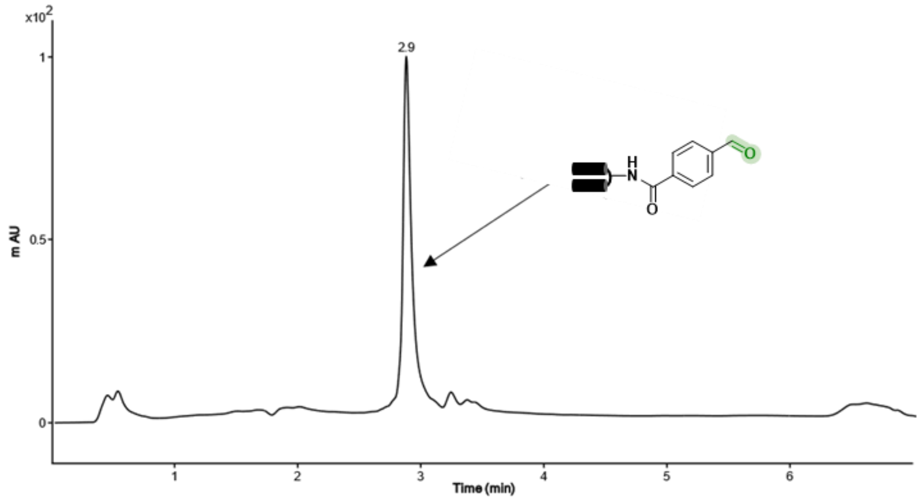

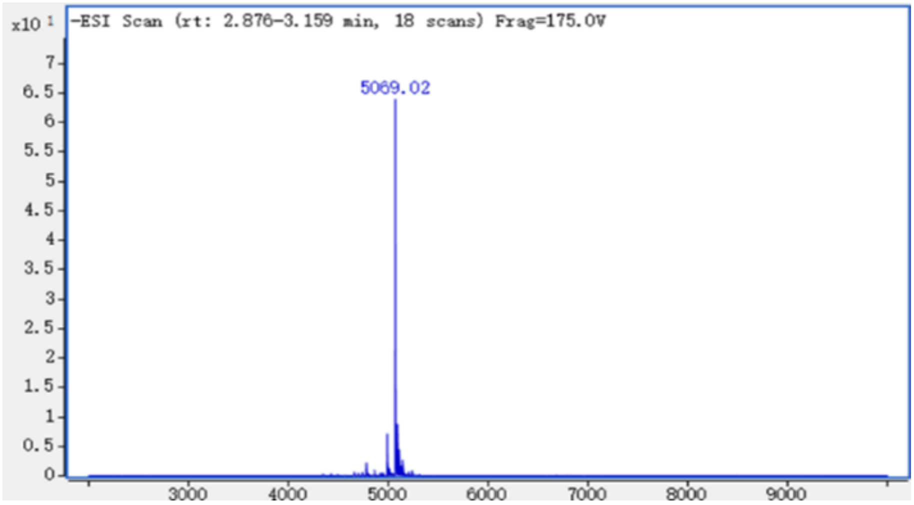

UPLC chromatogram and deconvoluted MS of **c10**

Conversion: ＞90%

Calculated Mass: 5069 Da; Observed Mass: 5069 Da

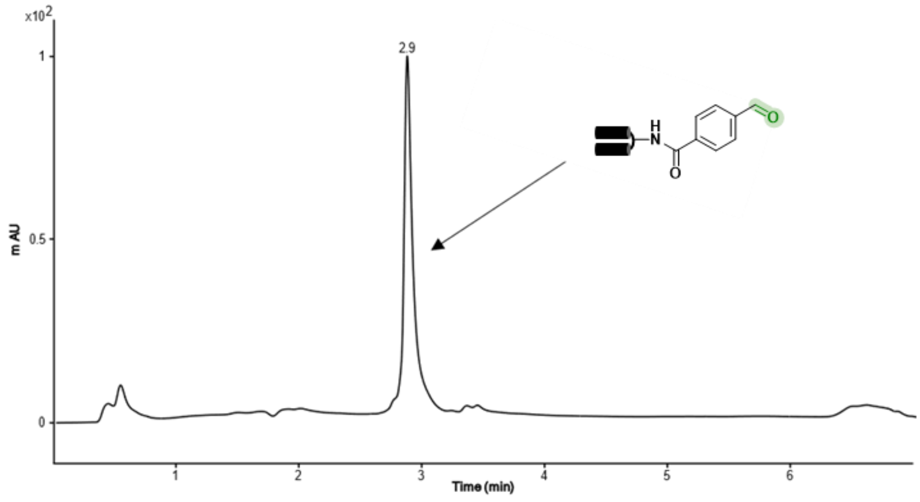

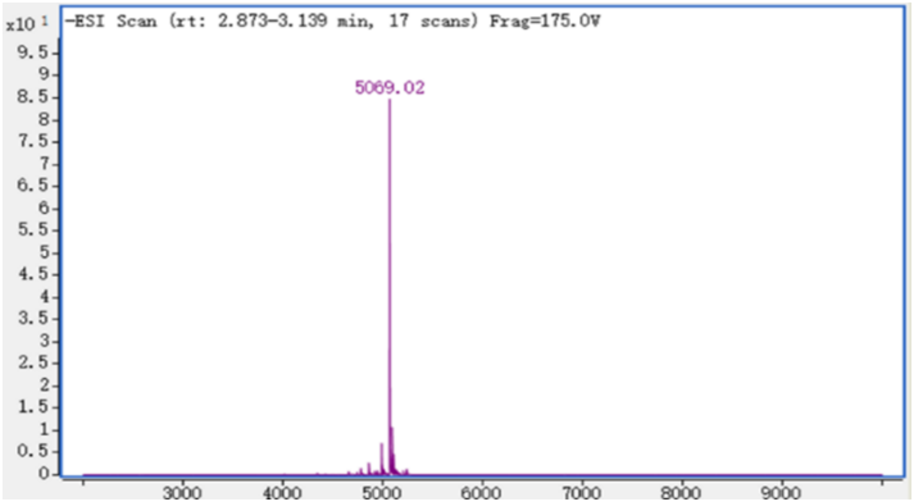

UPLC chromatogram and deconvoluted MS of **c11**

Conversion: ＞90%

Calculated Mass: 5069 Da; Observed Mass: 5069 Da

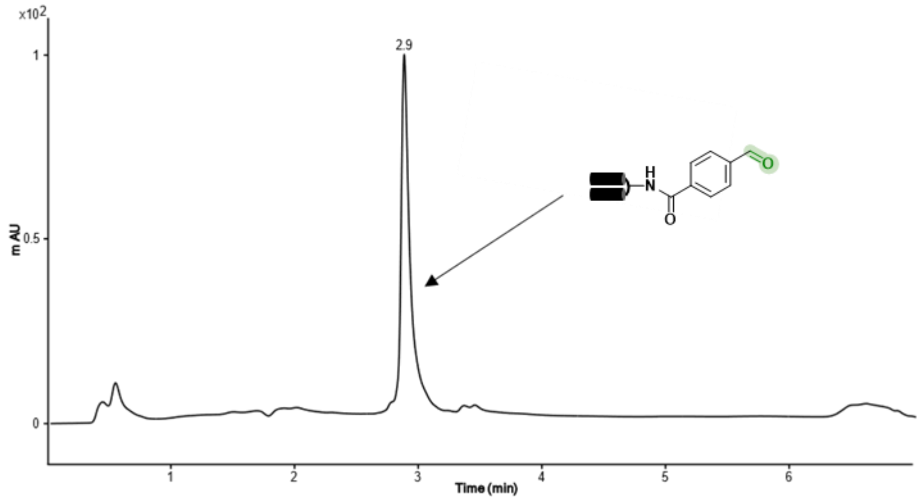

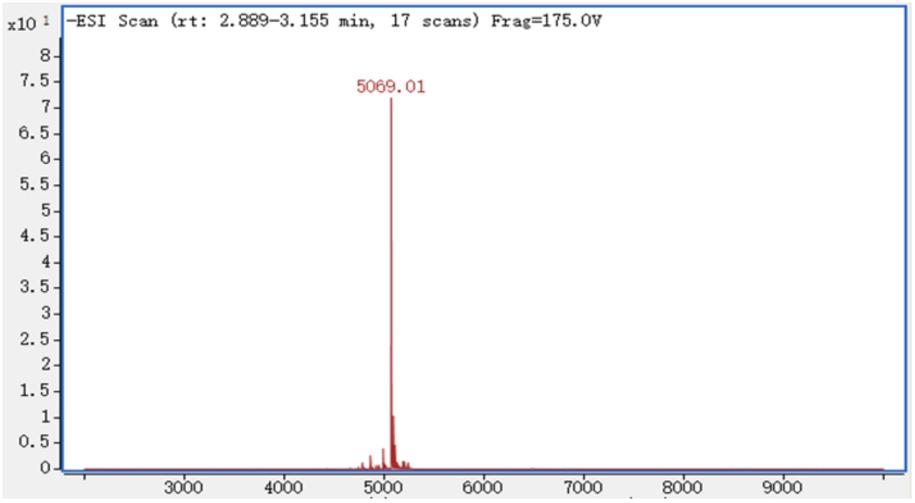

UPLC chromatogram and deconvoluted MS of **c12**

Conversion: ＞90%

Calculated Mass: 5069 Da; Observed Mass: 5069 Da

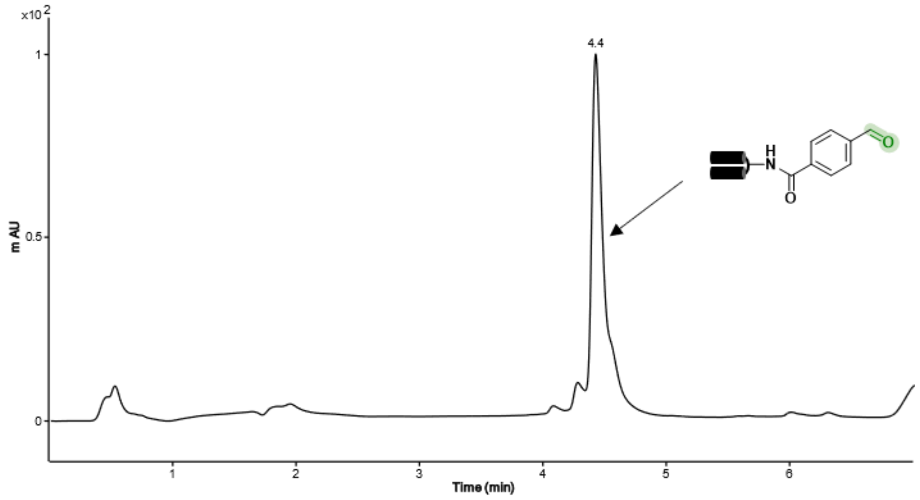

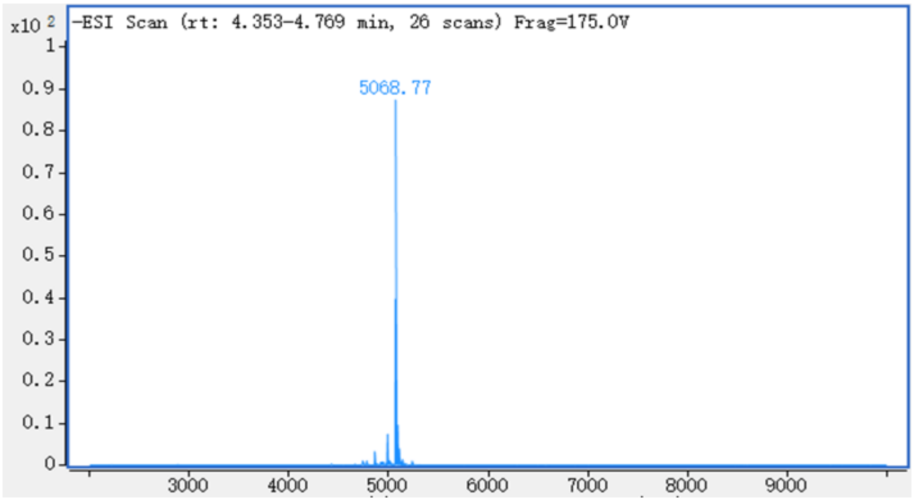

UPLC chromatogram and deconvoluted MS of **c13**

Conversion: ＞90%

Calculated Mass: 5069 Da; Observed Mass: 5069 Da

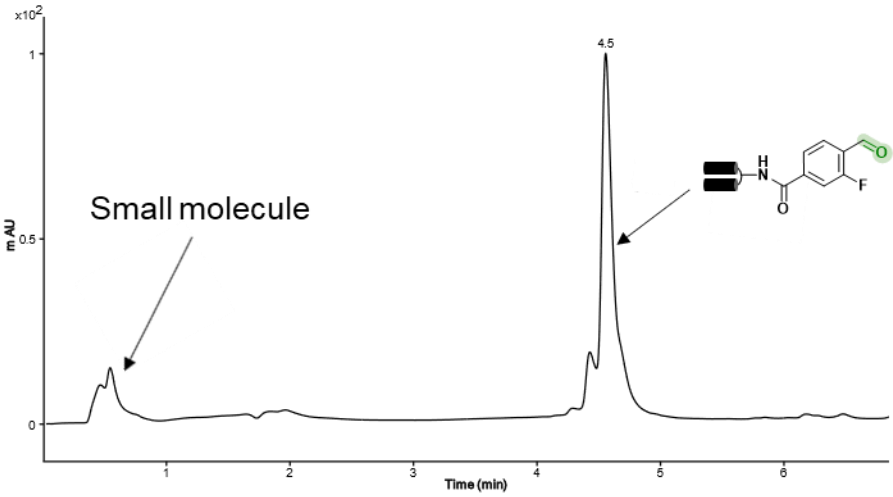

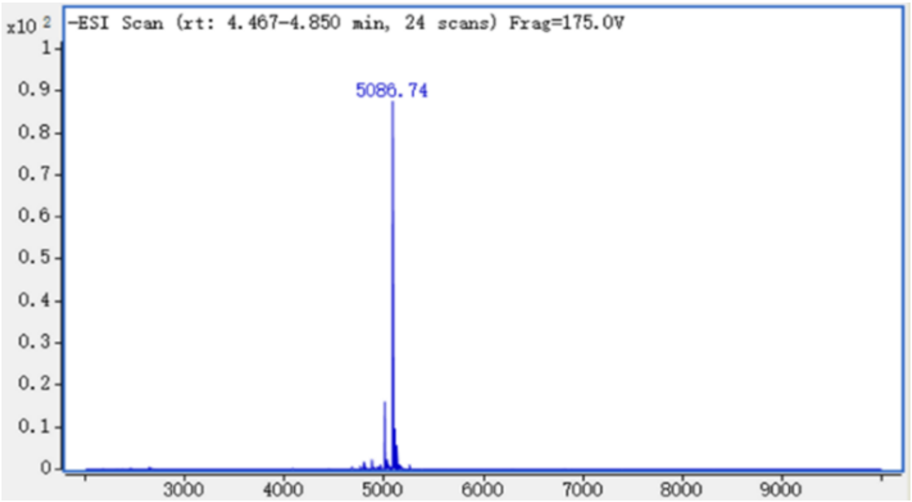

UPLC chromatogram and deconvoluted MS of **c14**

Conversion: ＞90%

Calculated Mass: 5075 Da; Observed Mass: 5075 Da

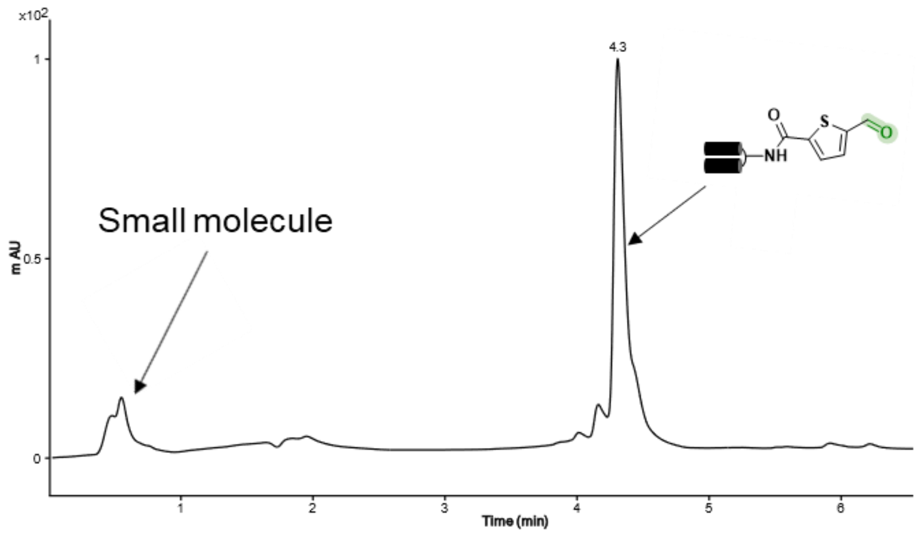

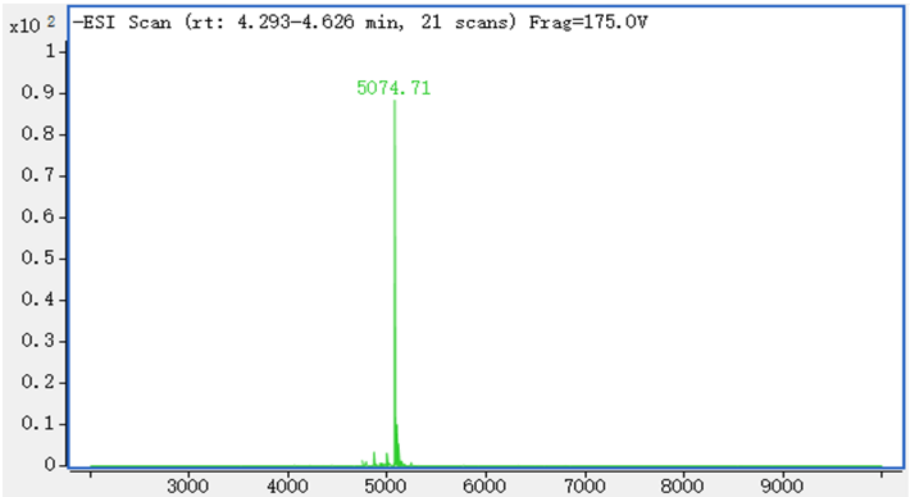

UPLC chromatogram and deconvoluted MS of **c15**

Conversion: ＞90%

Calculated Mass: 5069 Da; Observed Mass: 5069 Da

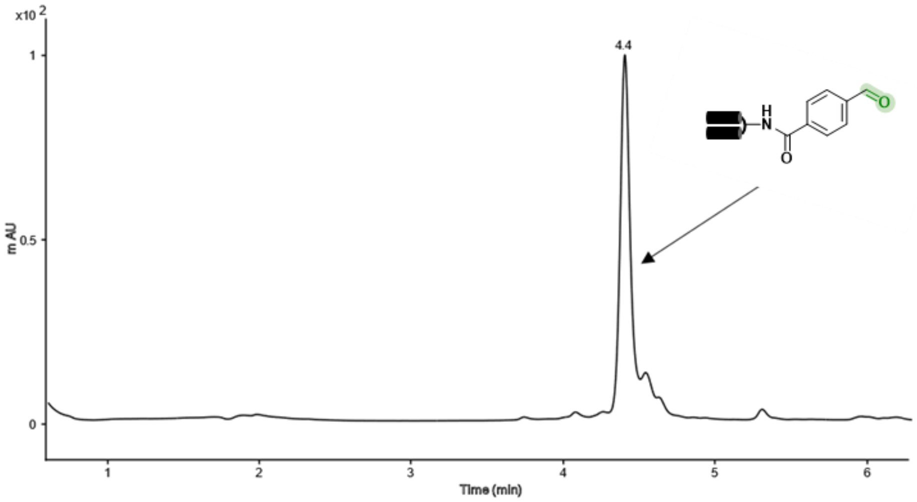

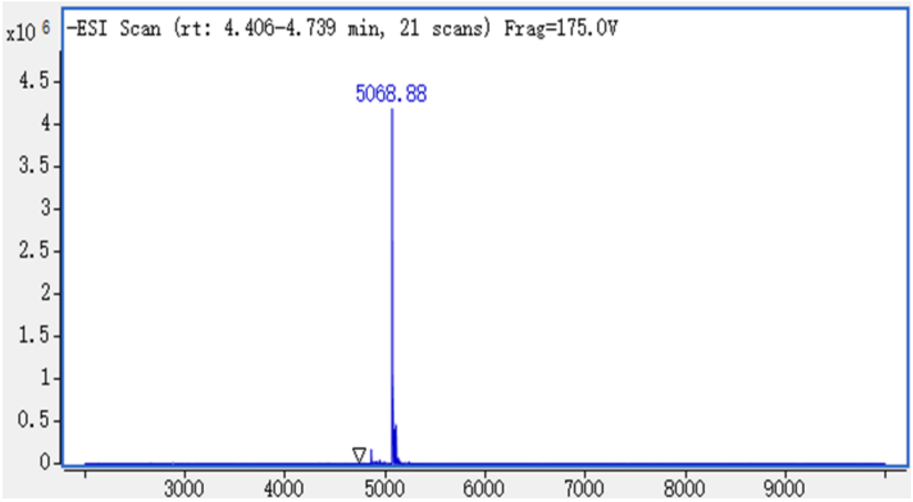

UPLC chromatogram and deconvoluted MS of **c16**

Conversion: >90%

Calculated Mass: 5069 Da; Observed Mass: 5069 Da

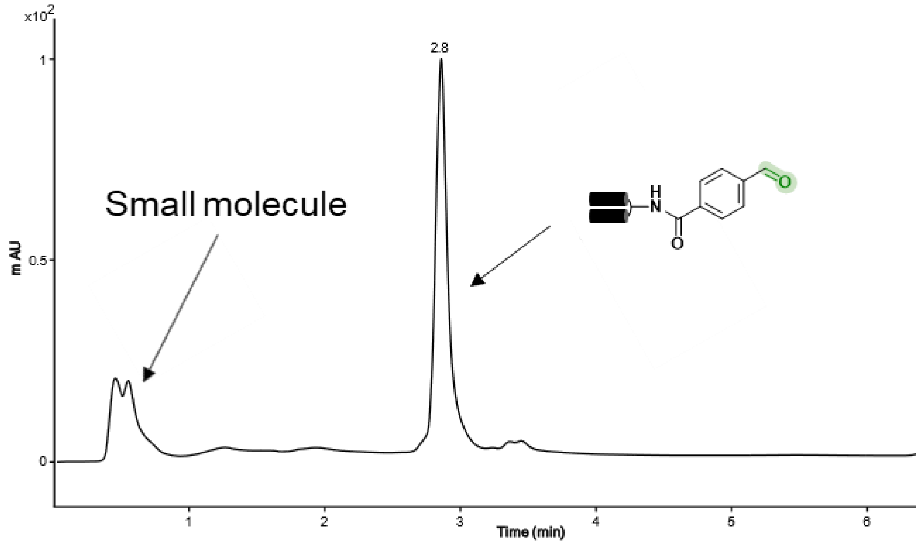

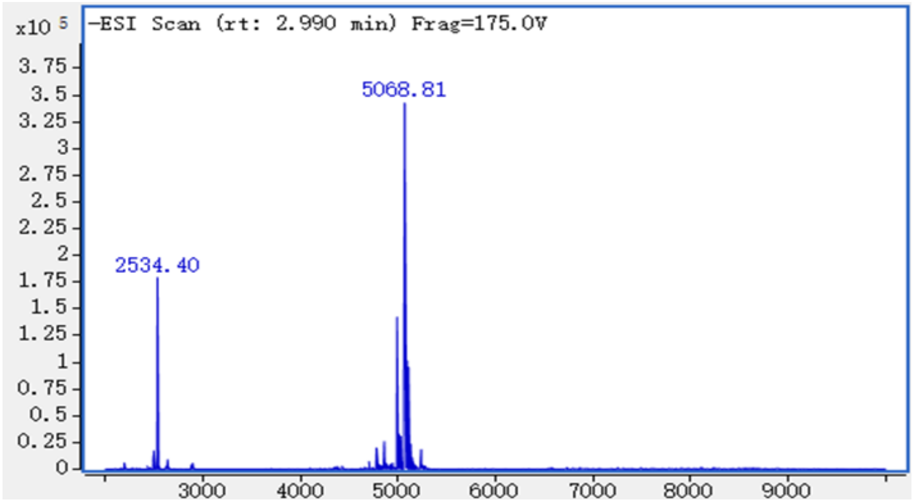

9.3 Reactivity validation of α-Carbon substituted substrates oxidized to ketones

DNA **b21** (0.2 nmol, 1 equiv.) was mixed with pH 5.5 phosphate buffer (10 µL, 200 mM in H_2O) and hydroxylamine hydrochloride (10 µL, 200 mM in DMA, 1000 nmol, 5000 equiv.). The reaction mixture was vortexed, centrifuged, and incubated at 30 °C for 8 hours. The product was purified by ethanol precipitation and analyzed using UPLC-MS, with a conversion rate exceeding 90%.

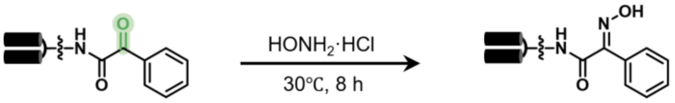

b21

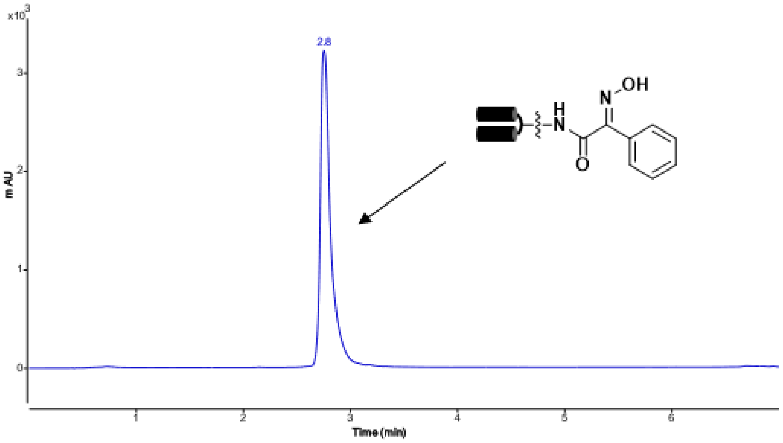

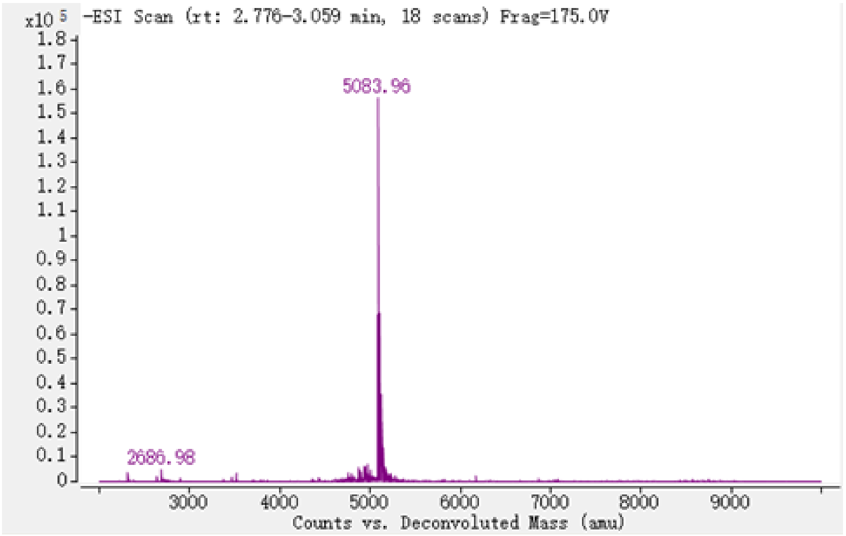

Figure S15. Verification of Ketone Reactivity.

9.4 Substrate scope of cleavage of DNA-conjugated secondary or tertiary phenylamine

UPLC chromatogram and deconvoluted MS of **e1**

Conversion: ＞90%

Calculated Mass: 5056 Da; Observed Mass: 5056 Da

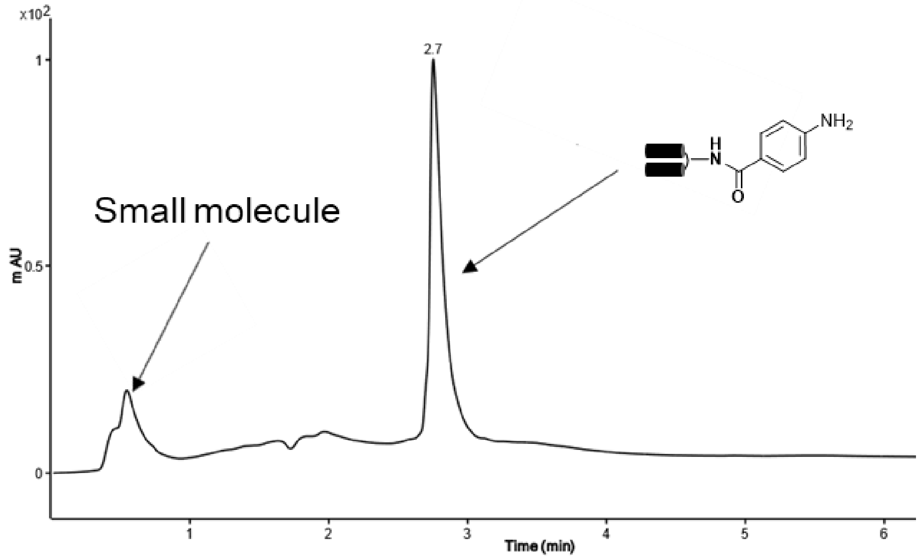

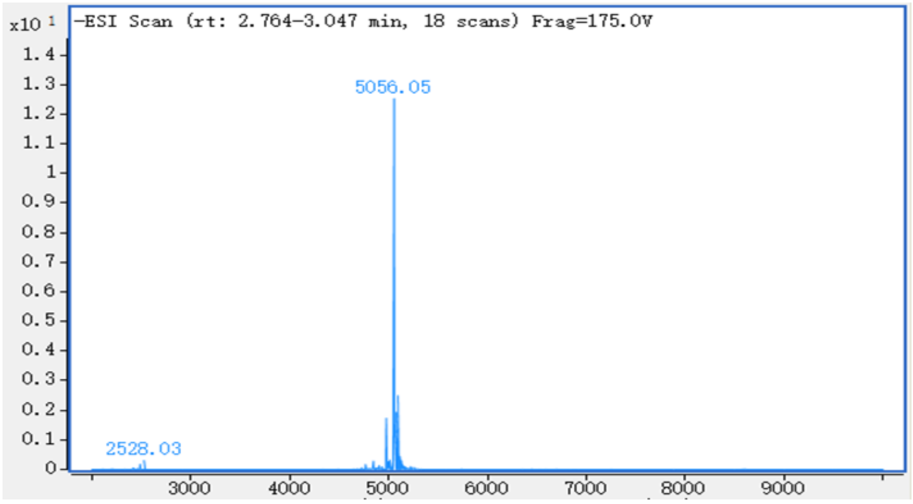

UPLC chromatogram and deconvoluted MS of **e2**

Conversion: ＞90%

Calculated Mass: 5056 Da; Observed Mass: 5056 Da

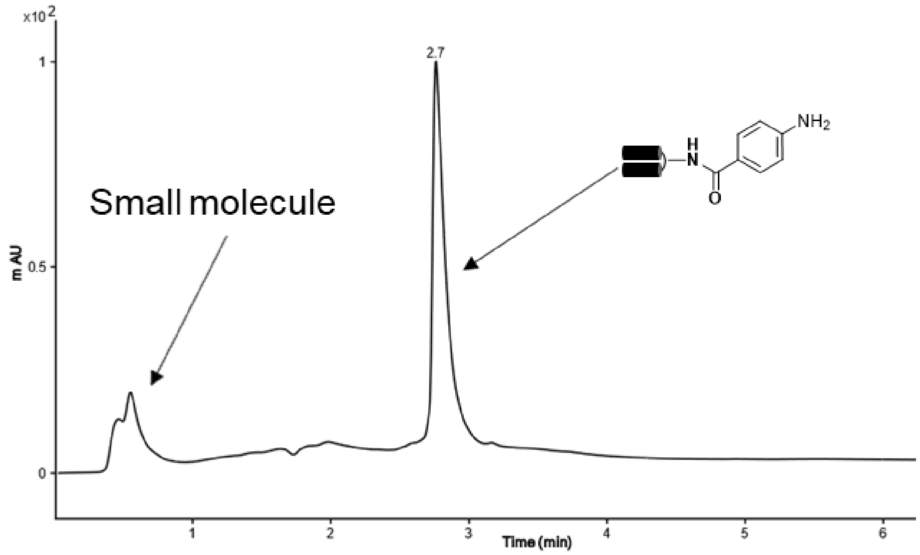

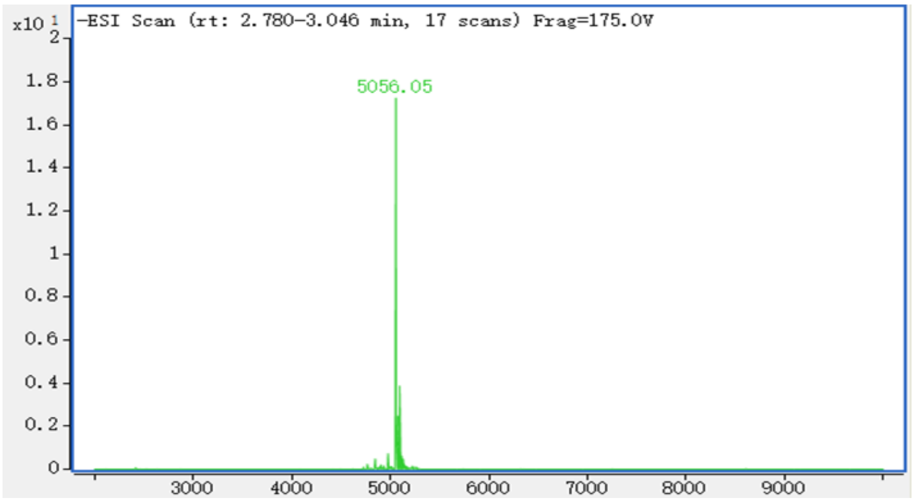

UPLC chromatogram and deconvoluted MS of **e3**

Conversion: 72%

Calculated Mass: 5056 Da; Observed Mass: 5056 Da

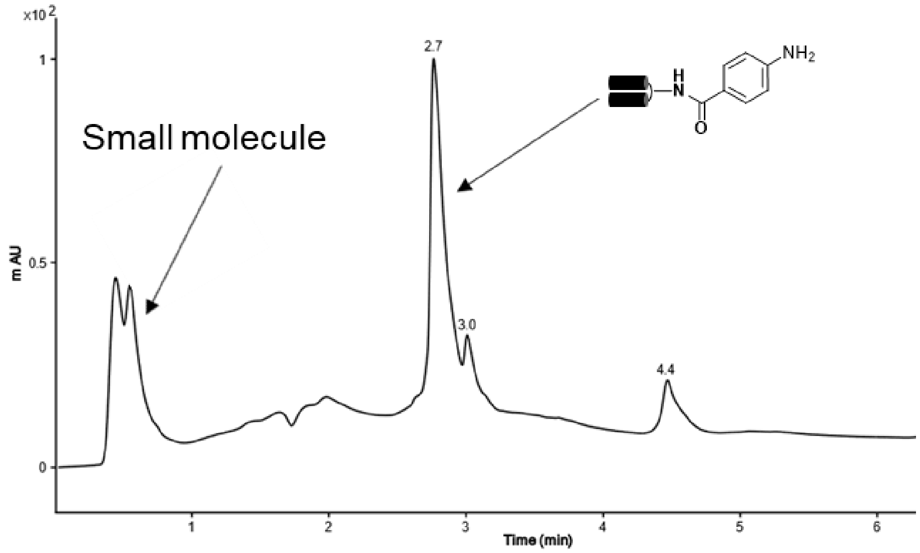

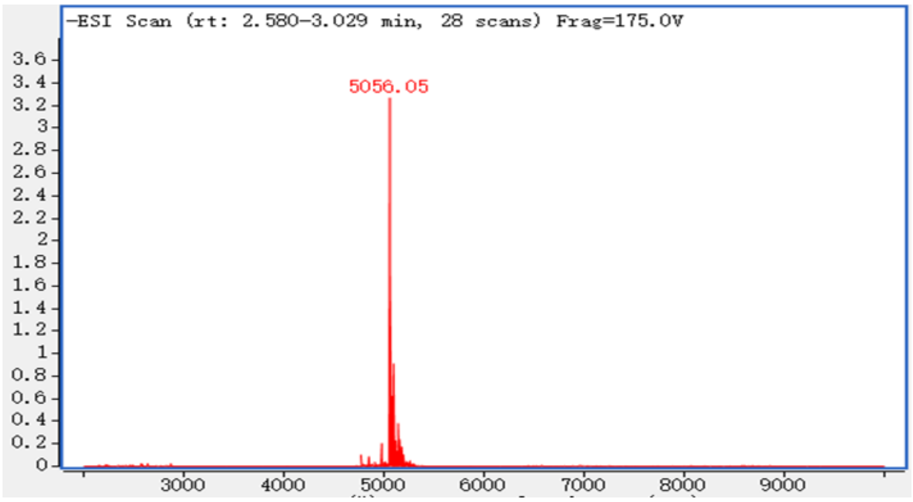

9.5 Reactivity validation of the cleavage of DNA-conjugated secondary or tertiary phenylamines into phenylamines

DNA-phenylamine **f1** (0.2 nmol, 1 equiv.) was mixed with pH 5.5 phosphate buffer (16 μL, 200 mM in H_2O), *p*-tolualdehyde (2 μL, 200 mM in DMA, 400 nmol, 2000 equiv.), and $NaBH_3CN$ (2 μL, 200 mM in H_2O, 400 nmol, 2000 equiv.). The reaction mixture was vortexed, centrifuged, and incubated at 60 °C for 8 hours. The product was purified by ethanol precipitation and analyzed using UPLC-MS, with a conversion rate exceeding 90%.

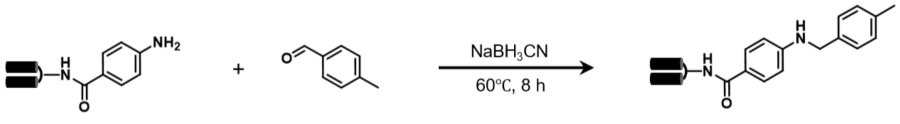

f1

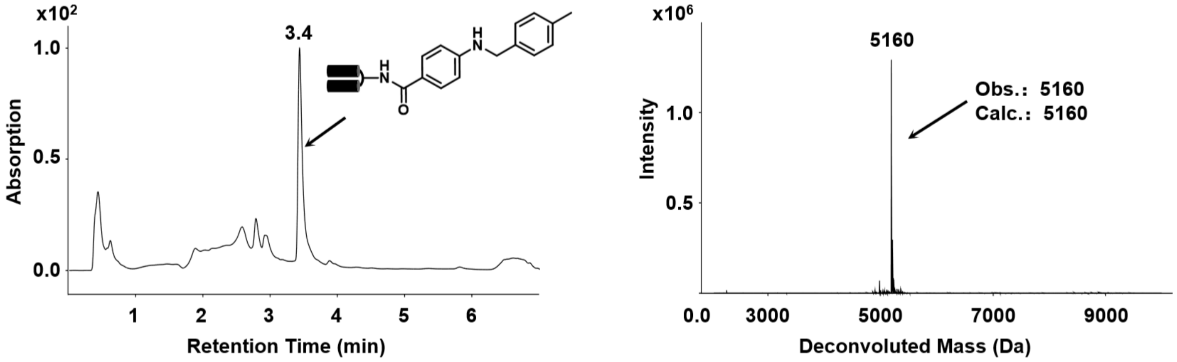

Figure S16. Verification of phenylamine reactivity.

10. DNA-templated system to validate the oxidative cleavage products

In Figure 4b, DNA **T1** (10 nmol, 1 equiv.) was mixed with sodium acetate buffer (CH_3COONa) (32 µL, 200 mM, pH 5.5), Laccase (4 µL, 0.1 U/µL in H_2O), and TEMPO (4 µL, 400 mM in 1,4-dioxane, 1600 nmol, 160 equiv.). The reaction mixture was vortexed, centrifuged, and incubated at 25 °C for 24 hours. The product was purified by ethanol precipitation and immediately analyzed using UPLC-MS, with a conversion rate exceeding 90%. Deconvoluted molecular mass: calculated 9471 Da, observed 9471 Da.

Reductive amination: 500 pmol of the substrate DNAs (**T2** and **B1**) in 300 µL buffer (100 mM MOPS, 1.0 M NaCl, pH 6.0) was heated at 95 °C for 5 min and slowly cooled to 25 °C in 1 hour. To this solution, 3 µL of a 1 M solution of $NaBH_3CN$ in H_2O was added and the resulting solution was briefly vortexed and then left at 25 °C for 14 hours; the DNA was then recovered by ethanol precipitation. The product was obtained by ethanol precipitation and analyzed by UPLC-MS immediately (Conversion: >90%). Deconvoluted molecular mass: calculated: 15313 Da; observed: 15313 Da.

DNA **T2-B1** (cross-linked) (500 nmol, 1 equiv.) was added CH_3COONa buffer (16 µL, 200 mM, pH 5.5), Laccase (2 µL, 0.1 U/µL in H_2O), and TEMPO (2 µL, 400 mM in 1, 4-dioxane, 800 nmol, 1600 equiv.). The reaction mixture was vortexed, centrifuged, and placed at 25 °C for 24 h. The product was obtained by ethanol precipitation and analyzed by UPLC-MS immediately. Deconvoluted molecular mass: calculated: 5857 Da, 9471 Da; observed: 5857 Da, 9471 Da.

11. Bioconjugation of *in situ* generated aldehyde-functionalized oligonucleotides

11.1 Reactive aldehyde handle for DNA labeling

In figure 5a, DNA-conjugated A (10 nmol, 1 equiv.) was added CH_3COONa buffer (32 μL, 200 mM, pH 5.5), Laccase (4 μL, 0.1 U/μL in H_2O), and TEMPO (4 μL, 400 mM in 1, 4-dioxane, 1600 nmol, 160 equiv.). The reaction mixture was vortexed, centrifuged, and incubated at 25 °C for 24 h. The product was obtained by ethanol precipitation and analyzed by UPLC-MS immediately (Conversion: >90%). Deconvoluted molecular mass: calculated: 4936 Da; observed: 4936 Da.

DNA-conjugated B (10 nmol, 1 equiv.) was dissolved in 10 μL H_2O, then add 2, 4-Dinitrophenylhydrazine (5 μL, 250 mM in DMSO, 1250 nmol, 125 equiv.), DMSO (5 μL) to the reaction. The reaction was vortexed and incubated at 25 °C for 30 minutes. The product was obtained by ethanol precipitation and analyzed by UPLC-MS immediately (Conversion: >70%). The separated and collected conjugates were purified by preparative HPLC and vacuum-dried overnight, redissolved in H_2O for subsequent experiments. Deconvoluted molecular mass: calculated: 5116 Da; observed: 5116 Da.

The UV-Vis absorption spectra of DNA-conjugated A B C samples were obtained using an enzyme-linked spectrophotometer.

11.2 Conjugation of DNA-conjugated aldehyde with peptide

To the solution of DNA-conjugated aldehyde (10 μL, 30 μM in 500 mM pH 5.5 PB, 0.3 nmol) was added aminooxy-modified peptide (10 μL, 100 mM in H_2O, 1000 nmol). The reaction mixture was vortexed, centrifuged, and incubated at 30 °C for 4 h. The product was obtained by ethanol precipitation and analyzed by UPLC-MS.

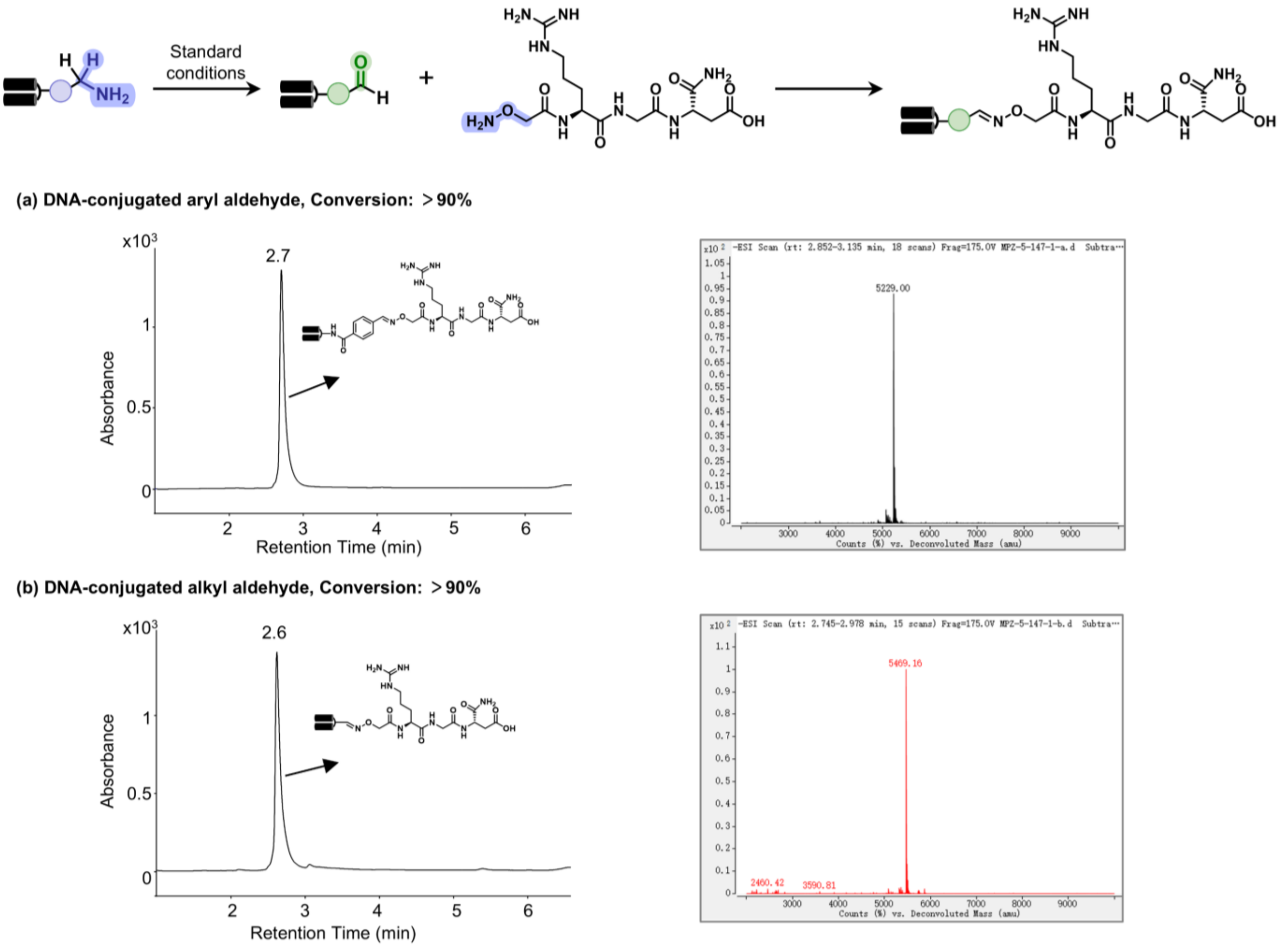

Figure S17. Oxime ligation of DNA-conjugated alkyl and aryl aldehydes with the aminooxy-modified RGD peptide.

To the solution of DNA-conjugated aldehyde (5 μL, 60 μM in H_2O, 0.3 nmol) was added triethylamine (3 μL, 200 mM in H_2O, 600 nmol), TCEP (3 μL, 10 mM in H_2O, 30 nmol), *N*-terminal cysteine-containing peptide (6 μL, 100 mM in H_2O, 600 nmol) and 15 μL MeOH. The reaction mixture was vortexed, centrifuged, and incubated at 25 °C for 13 h. The product was obtained by ethanol precipitation

and analyzed by UPLC-MS.

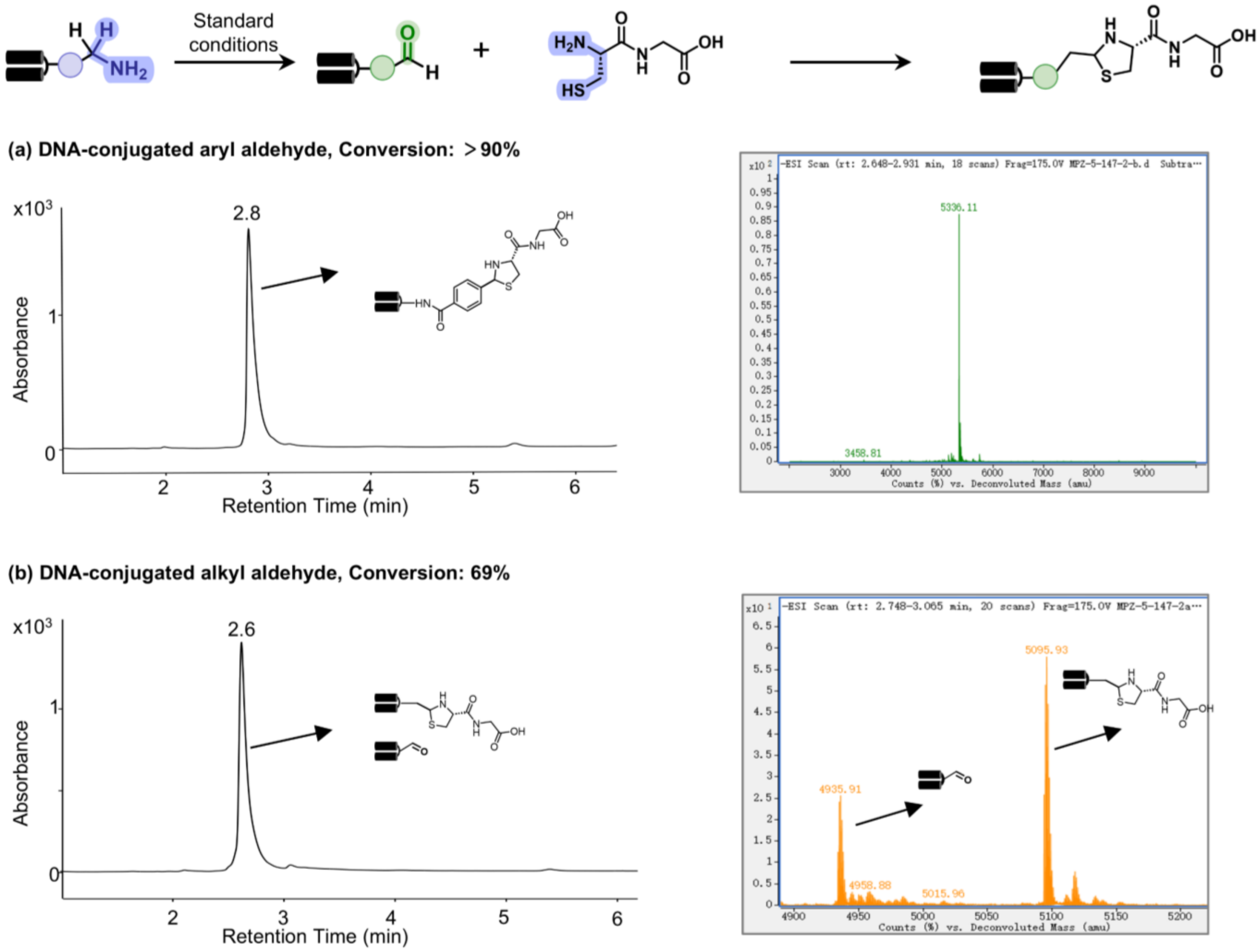

Figure S18. *N*-terminal cysteine-mediated labeling of DNA-conjugated alkyl and aryl aldehydes. In (b), the conversion rate was calculated based on the TIC (total ion current) intensity obtained from BioConfirm 10.0. The total ion trace corresponding to the desired product peak was extracted, and the conversion percentage was determined using the following equation: Conversion (%) = [Total abundance of the desired product / Total abundance of recovered DNA] × 100%, assuming 100% DNA recovery

12. Aldehyde-based chemical derivatization reactions

12.1 On-DNA benzimidazole formation

A solution of DNA-CHO (Oxidation by Laccase and TEMPO) (4 μL, 50 μM in H_2O, 0.2 nmol) was mixed with DMSO (12 μL), o-Phenylenediamine (2 μL, 200 mM in DMSO, 400 nmol), and p-Tolyl disulfide (2 μL, 200 mM in DMA, 400 nmol). The reaction mixture was vortexed, centrifuged, and irradiated with a 23 W white CFL at 25 °C for 4 hours. The product was purified by ethanol precipitation and analyzed using UPLC-MS (conversion: greater than 90%).[8]

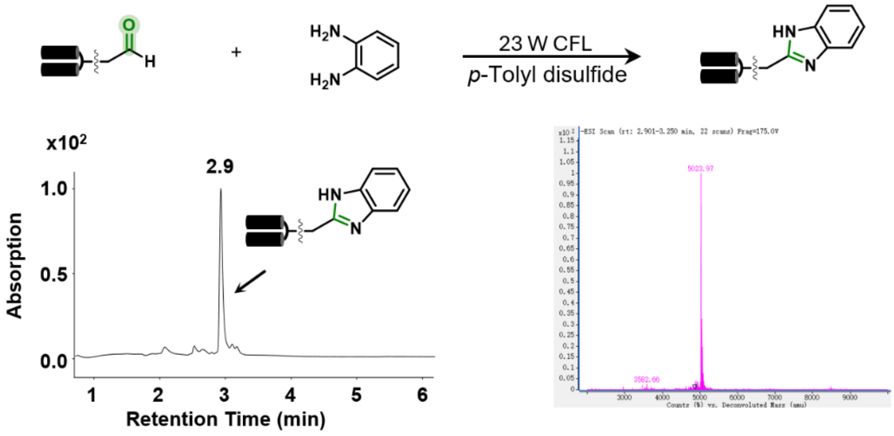

Figure S19. On-DNA benzimidazoles formation

12.2 On-DNA dihydroquinazolinone derivative formation

To the solution of DNA conjugate-CHO (2 μL, 100 μM in H_2O, 0.2 nmol) was added 10 μL H_2O, anthranilamide (5 μL, 200 mM in MeOH, 1000 nmol), $SbCl_3$ (5 μL, 40 mM in MeOH, 200 nmol). The reaction mixture was vortexed, centrifuged, and incubated at 25 °C for 3 h.. After reaction, 30 equiv of sodium diethyldithiocarbamic acid compared with $SbCl_3$ were added to the mixture to scavenge Sb, and the reaction mixture was stood at 25 °C for 30 minutes. The mixture was centrifuged at 25 °C for 10 min at 13,500 rpm, and the resultant supernatant was collected. The product was obtained by ethanol precipitation and analyzed by UPLC-MS.

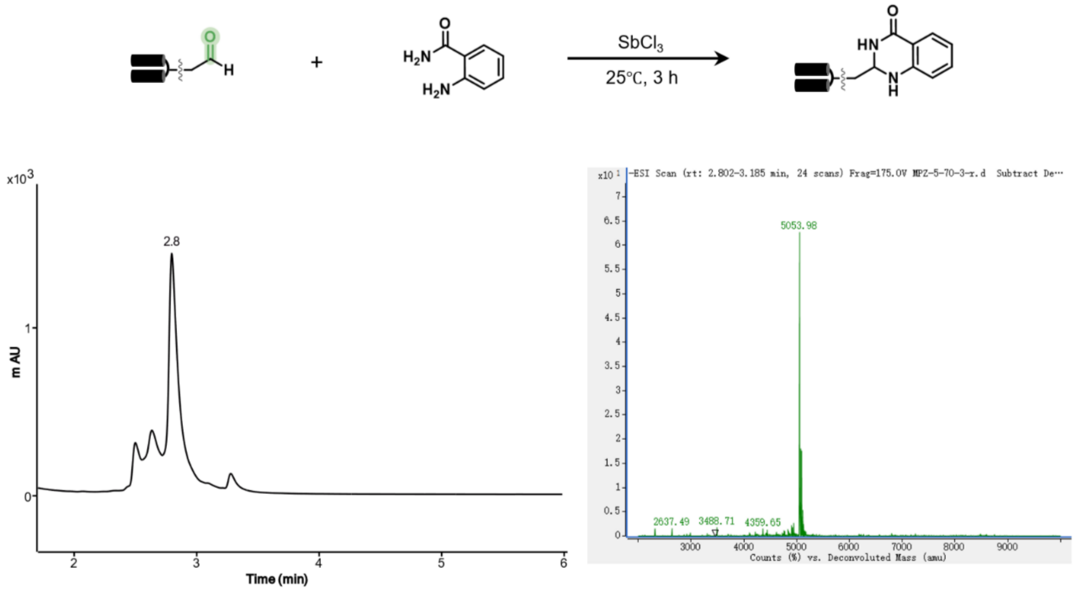

Figure S20. On-DNA dihydroquinazolinone derivative formation.

13. Reversible conjugation of DNA probes

The fixation procedure: The FAM-labeled or TAMRA-labeled DNA-conjugated amines (5 μM) were dissolved in PB (200 mM, pH 5.5). One droplet of the solution was applied to the aldehyde-modified glass slide. The glass slide was placed in Petri dishes with 4 x SSC solution and incubated at room temperature for 30 minutes. Subsequently, the glass slide was transferred to a hot plate set at 100 °C and dried for 30 minutes. This final drying step is crucial for dehydrating and condensing the aldehyde group with the amino group to form the Schiff base. The glass slide was then rinsed twice with 0.2% SDS solution for 2 minutes, followed by two rinses with deionized water for 2 minutes each. A solution of $NaBH_3CN$ (250 mM) in PB (pH 7.4, 200 mM) was added dropwise to the spotting location on the slide. To ensure complete coverage, the spotting location should be wider than the range of the initial spotting point. The slide was incubated for 16 hours in a biochemical incubator. Then the glass slide was washed as mentioned above.

The cleavage of the conjugated DNA from the glass slide: A drop of TEMPO/laccase solution in sodium acetate buffer (200 mM, pH 5.5) was added onto the glass slide. The reaction system was incubated for 24 hours at 25°C. Then the glass slide was washed as above mentioned.

14. Streamlined one-pot oxidation and diversification

Oxidation: DNA a1 b1 (5 nmol, 1 equiv.) was mixed with sodium acetate buffer (CH_3COONa) (16 µL, 200 mM, pH 5.5), Laccase (2 µL, 0.1 U/µL in H_2O), and TEMPO (2 µL, 400 mM in 1,4-dioxane, 800 nmol, 160 equiv.). The reaction mixture was vortexed, centrifuged, and incubated at 25 °C for 24 hours. The product was purified by ethanol precipitation and immediately analyzed using UPLC-MS, with a conversion rate exceeding 90%. 10 µL reaction mixture was purified by ethanol precipitation and immediately analyzed by UPLC-MS, confirming >90% conversion. The resulting DNA-conjugated aldehyde was subsequently used as the starting material for derivatization reactions. Route A: The purified DNA-aldehyde product was employed as the input material. Route B: the crude 10 µL reaction mixture was used directly without further purification.

Four derivatization reactions: Derivatization 1, A solution of DNA conjugate-CHO (Oxidation by laccase and TEMPO, 20 µM in H_2O, 0.2 nmol) was combined with DMSO (5 µL) and phenylhydrazine (5 µL, 200 mM in DMSO, 1000 nmol). The mixture was vortexed, centrifuged, and incubated at 25 °C for 4 hours. **Derivatization 2,** A solution of DNA conjugate-CHO (Oxidation by laccase and TEMPO, 12.5 µM in pH 5.5 500 mM phosphate buffer, 0.2 nmol) was combined with aniline (2 µL, 200 mM in DMA, 400 nmol) and sodium cyanoborohydride (2 µL, 200 mM in H_2O, 400 nmol). The mixture was vortexed, centrifuged, and incubated at 60 °C for 2 hours. **Derivatization 3,** A solution of DNA conjugate-CHO (Oxidation by laccase and TEMPO, 50 µM in H_2O, 0.2 nmol) was combined with DMSO (12 µL), o-Phenylenediamine (2 µL, 200 mM in DMSO, 400 nmol), and p-Tolyl disulfide (2 µL, 200 mM in DMA, 400 nmol). The reaction mixture was vortexed, centrifuged, and irradiated with a 23 W white CFL at 25 °C for 4 hours. **Derivatization 4,** DNA conjugate-CHO (Oxidation by laccase and TEMPO as described in Cycle 1; 10 µL, 20 µM in pH 5.5 500 mM phosphate buffer, 0.2 nmol) was reacted with hydroxylamine hydrochloride (10 µL, 200 mM in DMA, 2000 nmol). The mixture was vortexed, centrifuged, and incubated at 30 °C for 4 hours.

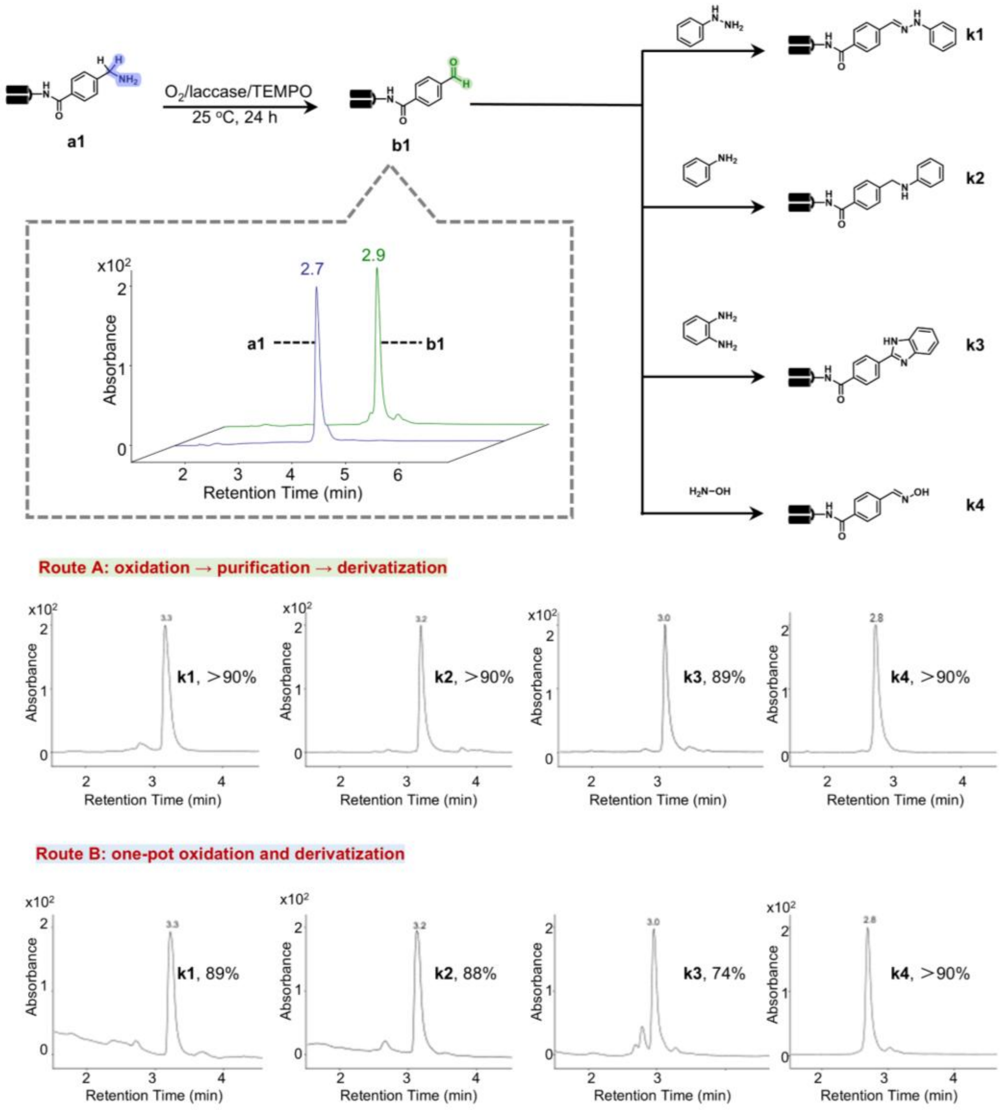

Figure S21. Comparison of stepwise and one-pot labeling strategies following O_2/laccase/TEMPO-mediated oxidation of DNA-conjugated aryl amine.

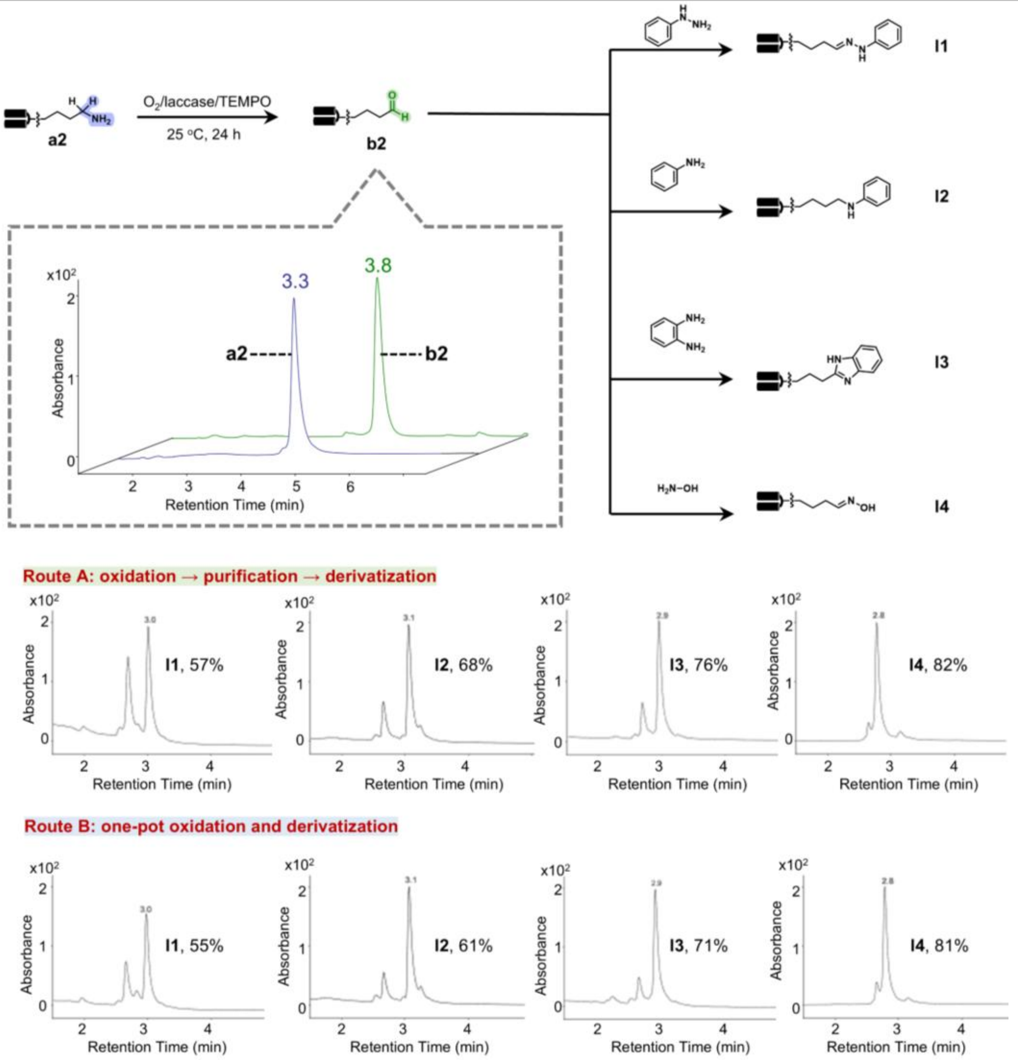

Figure S22. Comparison of stepwise and one-pot labeling strategies following O_2/laccase/TEMPO-mediated oxidation of DNA-conjugated alkyl amine.

15. Split-and-pool synthesis of a 4×3 mock library

We constructed a two-cycle mock library through a two-step DNA-encoded synthesis. In cycle 1, four DNA-conjugated primary and secondary amines were mixed as starting species and oxidized into four DNA-aldehydes via the TEMPO/laccase/O_2 system. In cycle 2, three derivatization reactions were employed in a "split-and-pool" combinatorial approach, ultimately yielding 12 product molecular species.

Cycle1: oxidation. A mixture of four DNA-conjugated amines (1.25 nmol each, 5 nmol total, 1 equiv.) was combined with CH_3COONa buffer (16 µL, 200 mM, pH 5.5), laccase (2 µL, 0.1 U/µL in H_2O) and TEMPO (2 µL, 400 mM in 1,4-dioxane, 800 nmol, 4000 equiv.). The reaction mixture was vortexed, centrifuged, and incubated at 25 °C for 24 h. The product was purified by ethanol precipitation and immediately subjected to UPLC-MS analysis. Four oxidized products were unequivocally identified by deconvoluted molecular mass analysis: calculated 5058 Da, 5069 Da, 5075 Da, 5082 Da; observed 5058 Da, 5069 Da, 5075 Da, 5082 Da.

Cycle2: Three derivatization reactions. **Derivatization 1:** A solution of DNA conjugate-CHO (Oxidation by Laccase and TEMPO as described in Cycle 1; 4 µL, 200 µM in H_2O, 0.8 nmol) was combined with DMSO (12 µL), o-Phenylenediamine (2 µL, 200 mM in DMSO, 400 nmol), and p-Tolyl disulfide (2 µL, 200 mM in DMA, 400 nmol). The reaction mixture was vortexed, centrifuged, and irradiated with a 23 W white CFL at 25 °C for 4 hours. **Derivatization 2:** DNA conjugate-CHO (Oxidation by Laccase and TEMPO as described in Cycle 1; 10 µL, 80 µM in H_2O, 0.8 nmol) was mixed with anthranilamide (5 µL, 200 mM in methanol, 1000 nmol) and antimony trichloride ($SbCl_3$; 5 µL, 40 mM in MeOH, 200 nmol). The mixture was vortexed, centrifuged, and incubated at 25 °C for 3 hours. After reaction, 30 equiv of sodium diethyldithiocarbamic acid compared with $SbCl_3$ were added to the mixture to scavenge Sb, and the reaction mixture was stood at 25 °C for 30 minutes. The mixture was centrifuged at 25 °C for 10 min at 13,500 rpm, and the resultant supernatant was collected. **Derivatization 3:** DNA conjugate-CHO (Oxidation by Laccase and TEMPO as described in Cycle 1; 10 µL, 80 µM in pH 5.5 500 mM phosphate buffer, 0.8 nmol) was reacted with hydroxylamine hydrochloride (10 µL, 200 mM in DMA, 2000 nmol). The mixture was vortexed, centrifuged, and incubated at 30 °C for 4 hours.

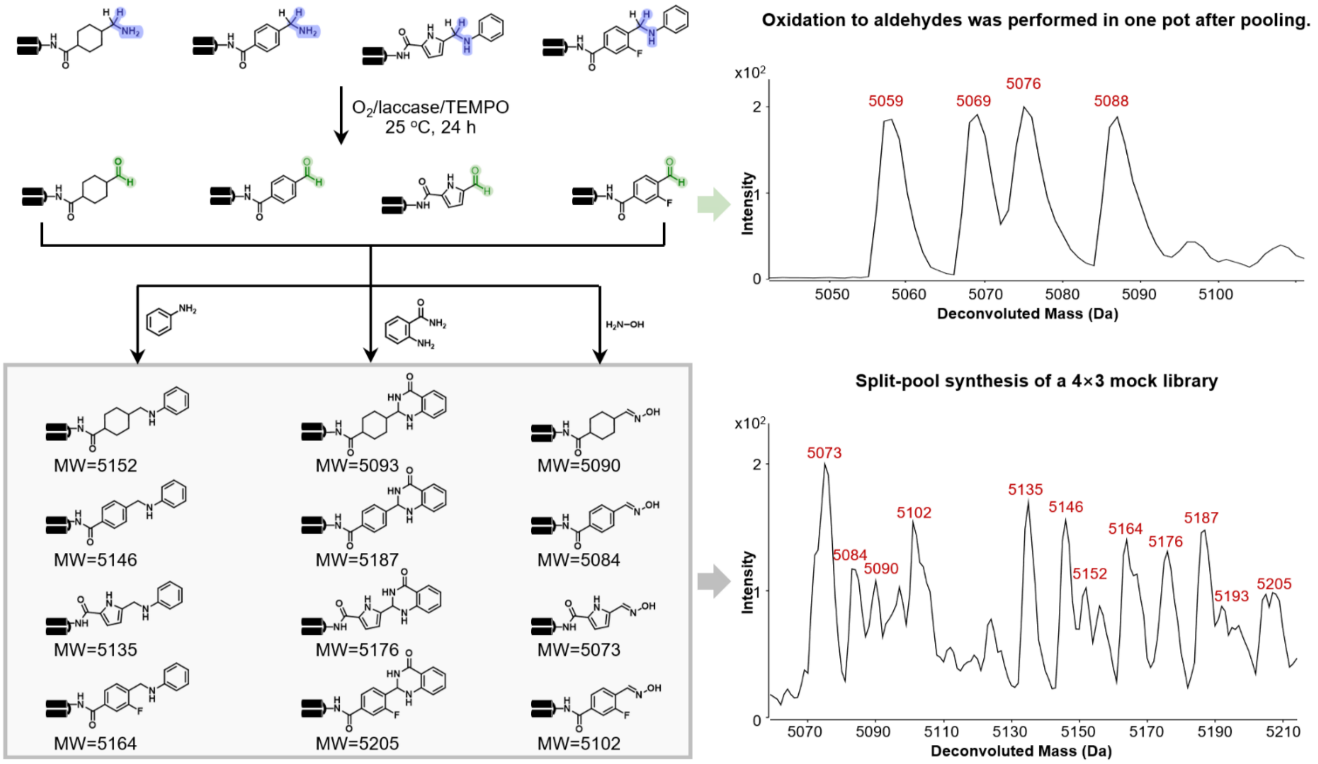

Figure S23. The split-and-pool synthesis of a 4×3 mock library based on the O_2/laccase/TEMPO-mediated oxidation and chemical diversification of aldehydes.

16. Transformation of amines to aldehydes enabled aldehyde-based multiple display of DELs

Fmoc-deprotection: To the DNA conjugate (10 nmol) was added piperidine solution (20% aq, 400 μL. Then the Tfa deprotection was allowed to proceed for 20 min at 25 ℃. The Fmoc-off conjugate was isolated by ethanol precipitation and quantified by UV absorption at 260 nm.

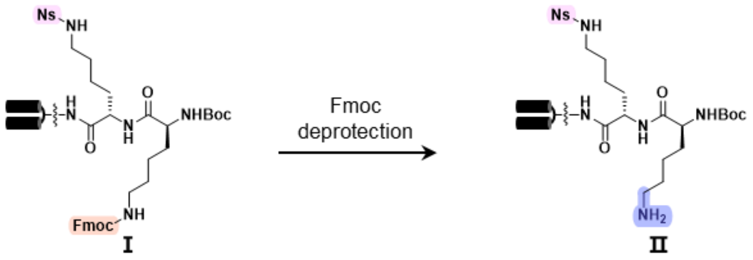

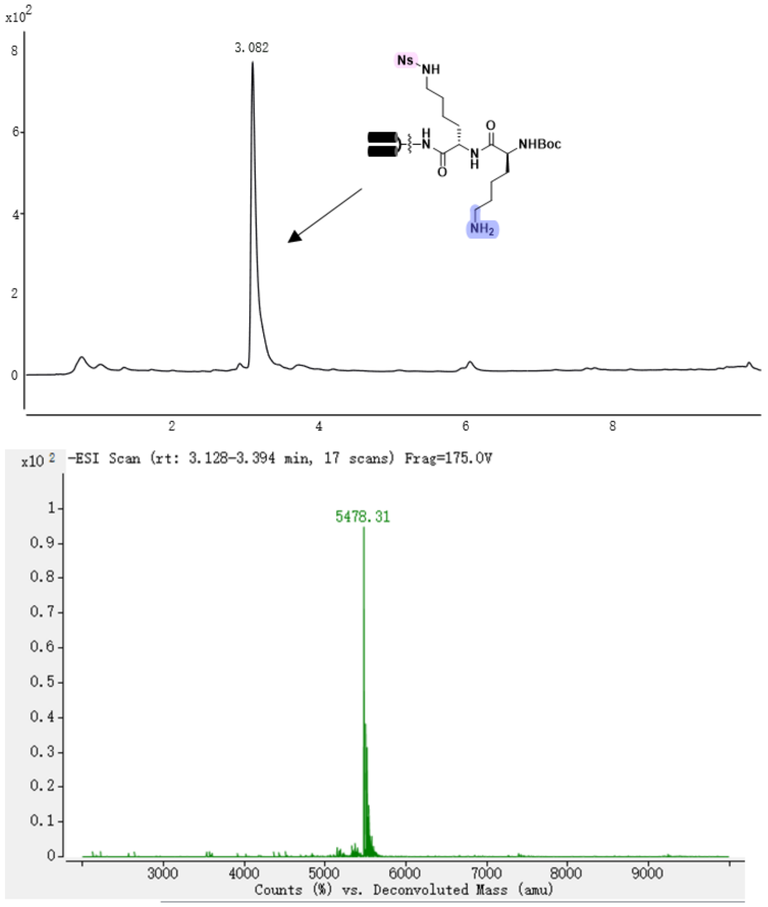

Figure S24. Characterization of Fmoc deprotection.

DNA (1 nmol) was added CH_3COONa buffer (16 μL, 200 mM, pH 5.5), Laccase (2 μL, 0.1 U/μL in H_2O), and TEMPO (2 μL, 400 mM in 1, 4-Dioxane, 800 nmol, 4000 equiv.). The reaction mixture was vortexed, centrifuged, and placed at 25 ºC for 24 h. The product was obtained by ethanol precipitation and analyzed by UPLC-MS. Deconvoluted molecular mass: calculated: 5477 Da; observed: 5477 Da. Unless otherwise noted, on-DNA amines described in the supporting information were synthesized under this standard condition.

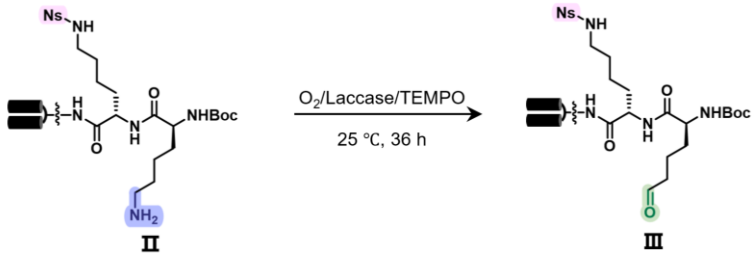

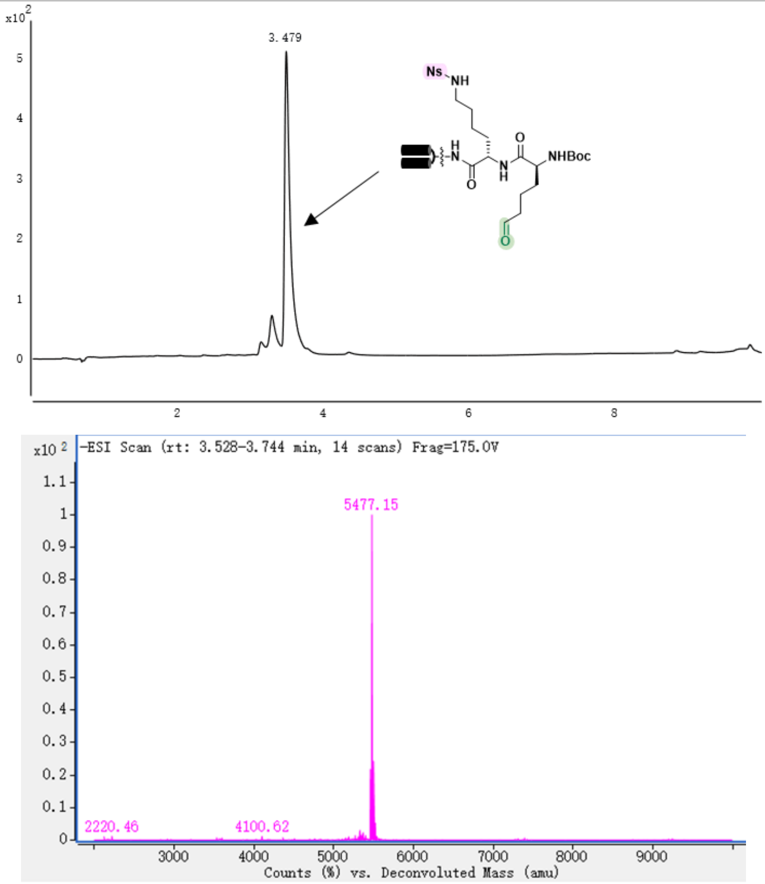

Figure S25. Characterization of oxidation to aldehyde.

To the solution of DNA conjugate-CHO (2 µL, 100 µM in H_2O, 0.2 nmol) was added 10 µL H_2O, anthranilamide (5 µL, 200 mM in MeOH, 1000 nmol), $SbCl_3$ (5 µL, 40 mM in MeOH, 200 nmol). The reaction mixture was vortexed, centrifuged, and incubated at 25 °C for 3 h. Then, 2 µL of a 200 mM solution of $NaIO_4$ in H_2O was added and the resulting solution was briefly vortexed and then left at 25 °C for 6 hours. After reaction, 30 equiv of sodium diethyldithiocarbamic acid compared with $SbCl_3$ were added to the mixture to scavenge Sb, and the reaction mixture was stood at 25 °C for 30 minutes. The mixture was centrifuged at 25 °C for 10 min at 13,500 rpm, and the resultant supernatant was collected. The product was obtained by ethanol precipitation and analyzed by UPLC-MS.

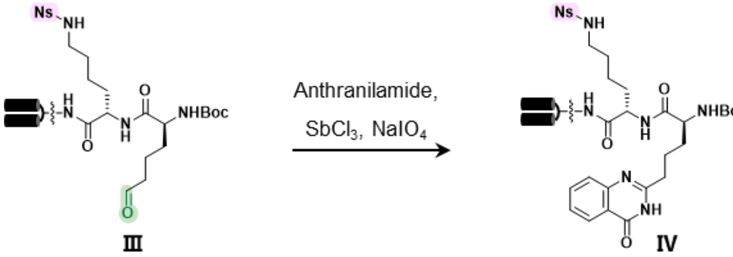

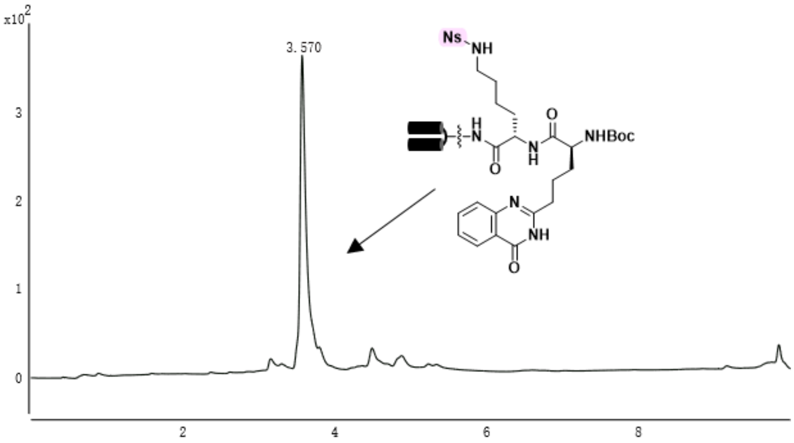

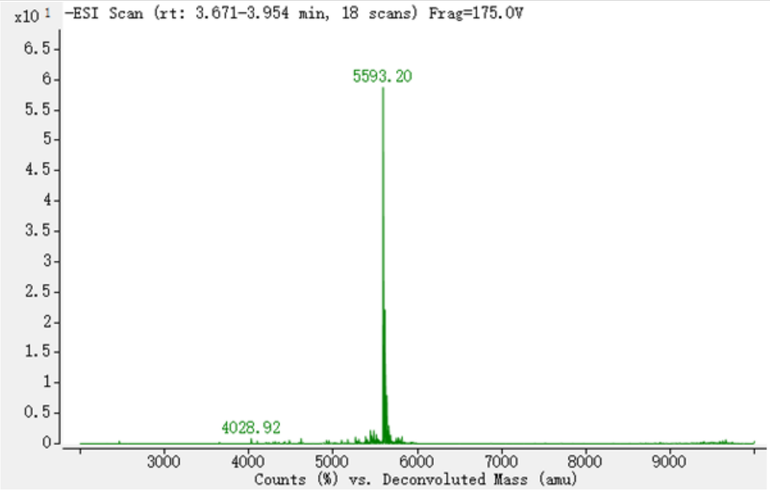

Figure S26. Characterization of derivatization.

Ns-deprotection: The DNA conjugate (10 nmol) in MOPS Buffer (100 µL, 300 mM, pH 8.2 buffer, 500 mM NaCl) was mixed with DBU (300 mM in DMSO, 100 µL) and BME (300 mM in DMSO, 100 µL). The mixture was degassed with N_2 for 30 s and agitated at RT for 10 h. The Ns-off conjugate was isolated by ethanol precipitation and quantified by UV absorption at 260 nm.

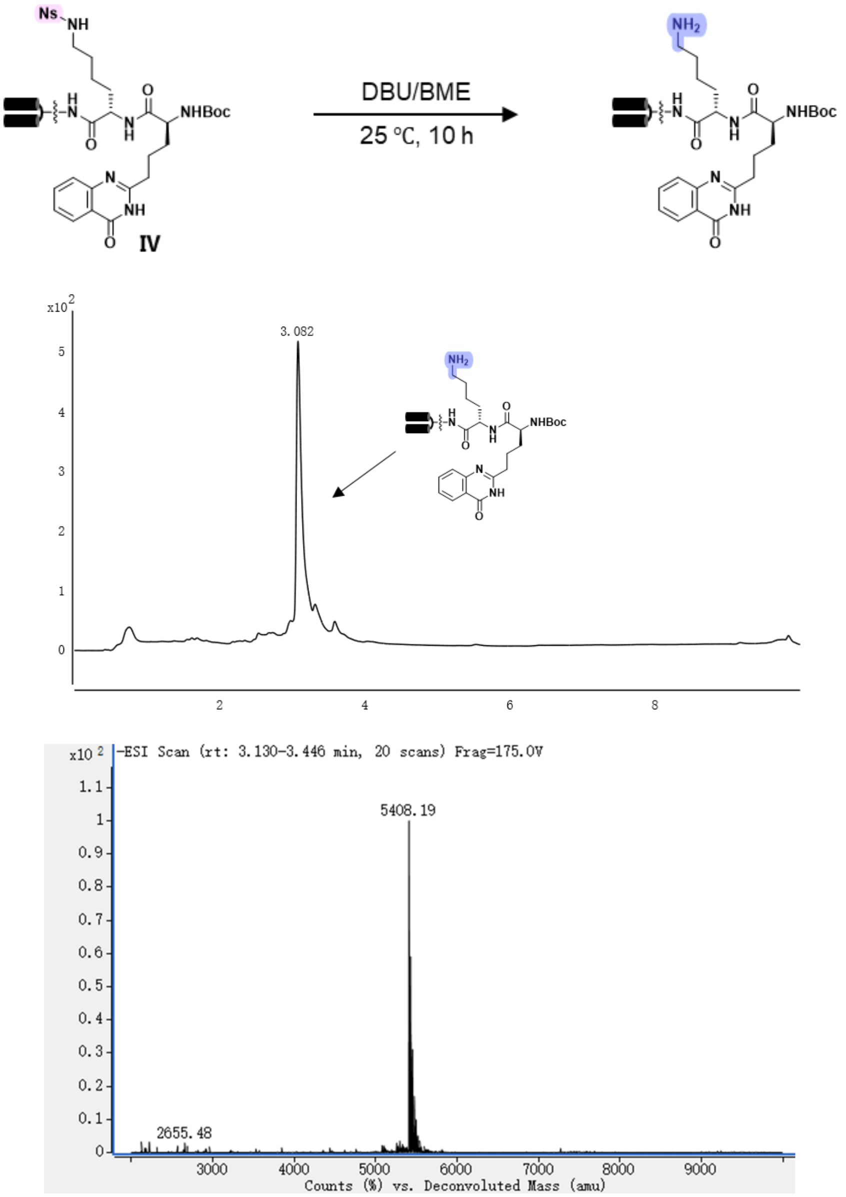

Figure S27. Characterization of Fmoc deprotection.

DNA (1 nmol) was added CH_3COONa buffer (16 µL, 200 mM, pH 5.5), Laccase (2 µL, 0.1 U/µL in H_2O), and TEMPO (2 µL, 400 mM in 1,4-Dioxane, 800 nmol, 4000 equiv.). The reaction mixture was vortexed, centrifuged, and placed at 25 °C for 24 h. The product was obtained by ethanol precipitation and analyzed by UPLC-MS. Deconvoluted molecular mass: calculated: 5407 Da; observed: 5407 Da. Unless otherwise noted, on-DNA amines described in the supporting information were synthesized under this standard condition.

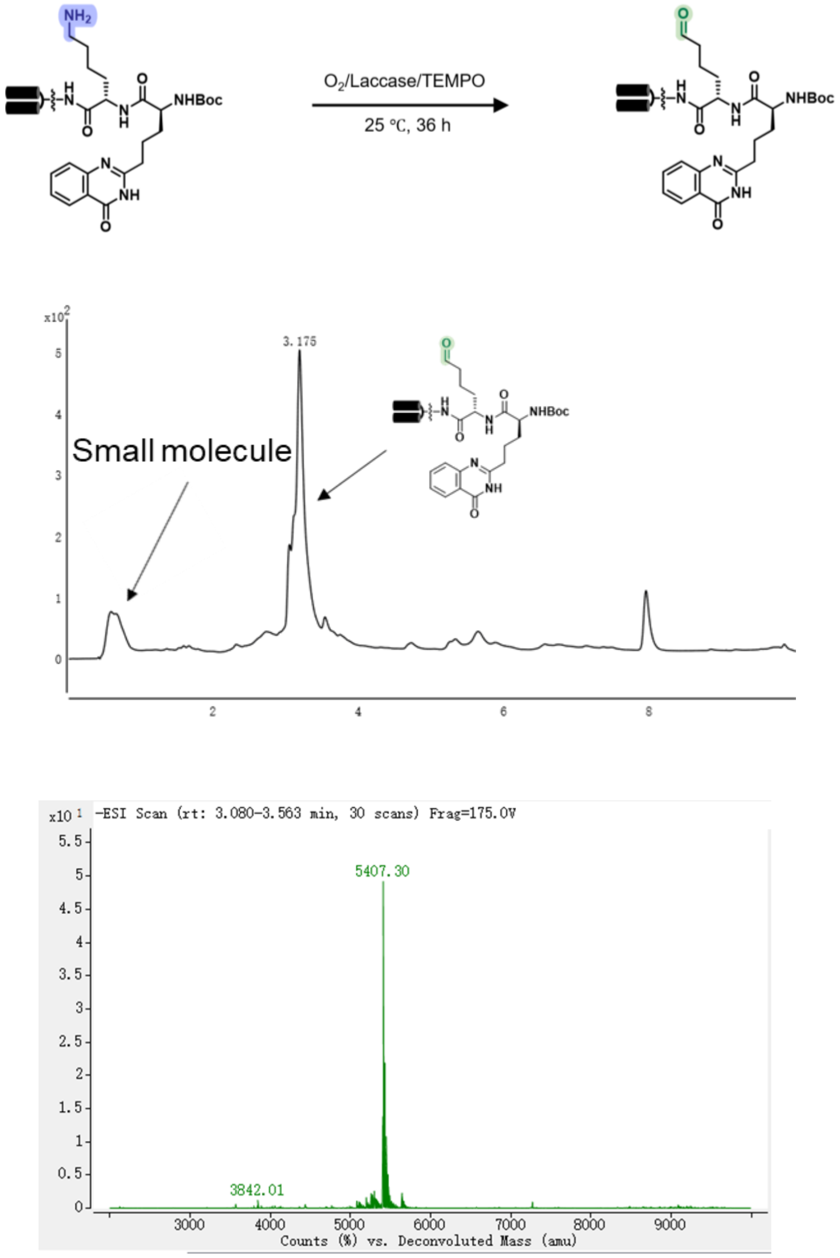

Figure S28. Characterization of oxidation to aldehyde.

To the solution of DNA conjugate-CHO (2 μL, 100 μM in H_2O, 0.2 nmol), 10 μL H_2O, anthranilamide (5 μL, 200 mM in MeOH, 1000 nmol), $SbCl_3$ (5 μL, 40 mM in MeOH, 200 nmol) was added. The reaction mixture was vortexed, centrifuged, and incubated at 25 °C for 3 h. After reaction, 30 equiv of sodium diethyldithiocarbamic acid compared with $SbCl_3$ were added to the mixture to scavenge Sb, and the reaction mixture was stood at 25 °C for 30 minutes. The mixture was centrifuged at 25 °C for 10 min at 13,500 rpm, and the resultant supernatant was collected. The product was obtained by ethanol precipitation and analyzed by UPLC-MS.

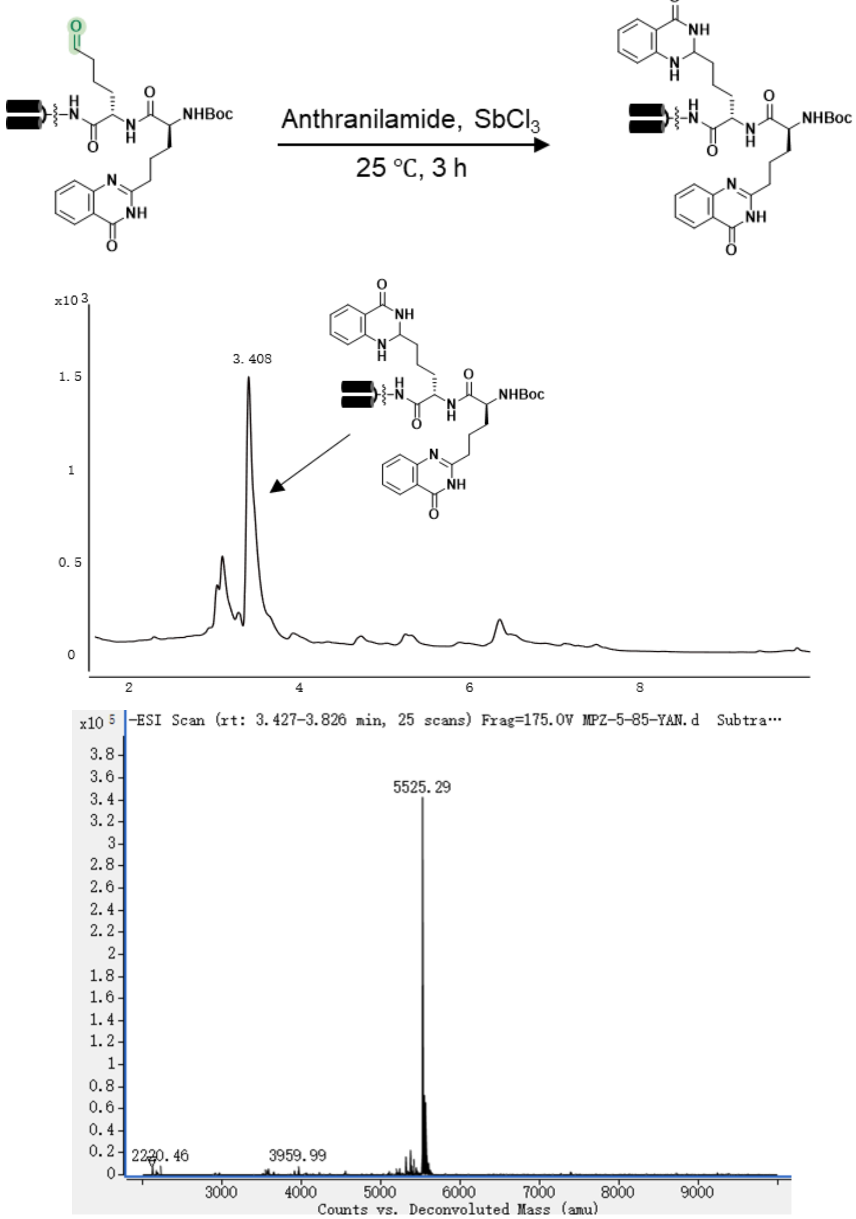

Figure S29. Characterization of derivatization.

17. Synthesis of quinazolinone alkaloid

17.1 On-DNA anthranilamide synthesis

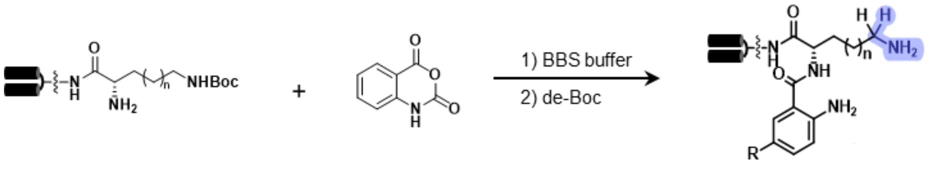

Figure S30. DNA-conjugated benzimidazoles synthesis.

DNA-conjugate **S3** was dissolved in sodium borate buffer (250 mM, pH 9.4) to make 1 mM solution. Isatoic anhydride (5 μL, 200mM in DMA, 1000 nmol, 100 equiv.) was added to DNA solution (20 μL, 20 nmol). The reaction mixture was vortexed, centrifuged, and placed at 25 °C for 2 h. After ethanol precipitation, the reaction was analyzed by UPLC-MS. The separated and collected conjugates were purified by preparative HPLC and vacuum-dried overnight, redissolved in H_2O for subsequent experiments.

17.2 Substrate expansion of quinazolinone alkaloid

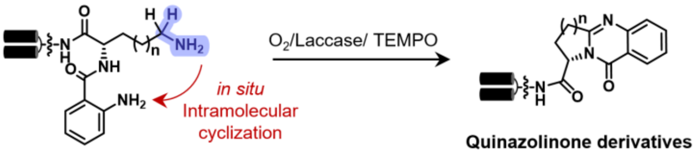

Figure S31. DNA-conjugated quinazolinone alkaloid synthesis.

DNA-conjugate **s5** (5 nmol, 1 equiv.) was mixed with CH_3COONa buffer (16 μL, 200 mM, pH 4.0), Laccase (2 μL, 0.1 U/μL in H_2O), and TEMPO (2 μL, 400 mM in 1, 4-dioxane, 800 nmol, 4000 equiv.). The reaction mixture was vortexed, centrifuged, and incubated at 25 °C for 24 h. The product was purified by ethanol precipitation and analyzed by UPLC-MS immediately (Conversion: >90%).

17.3 UPLC-MS Spectrum of DNA-conjugated quinazolinone alkaloid

UPLC chromatogram and deconvoluted MS of **g1**

Conversion: 60%

Calculated Mass: 5163 Da; Observed Mass: 5163 Da

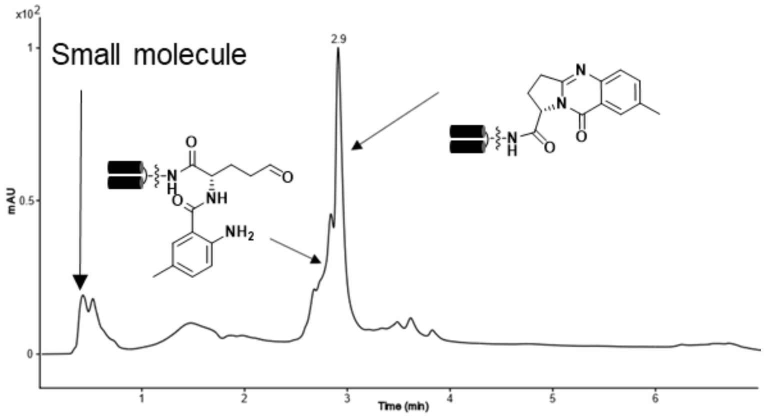

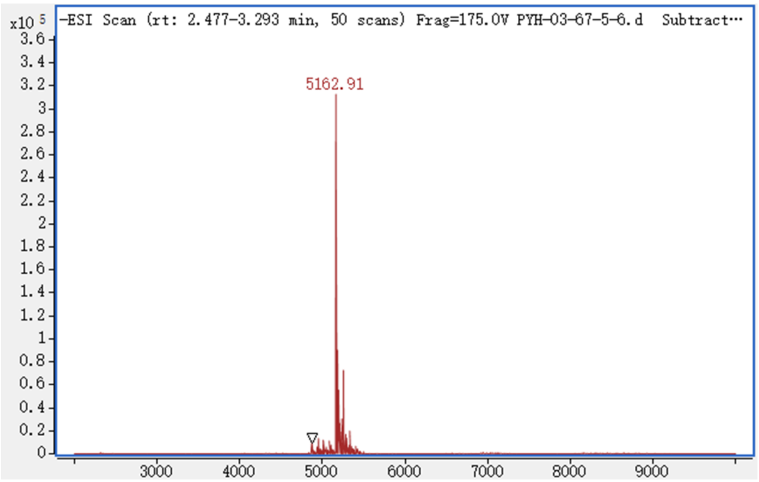

SUPPORTING INFORMATION

UPLC chromatogram and deconvoluted MS of **g2**

Conversion: 56%

Calculated Mass: 5149 Da; Observed Mass: 5149 Da

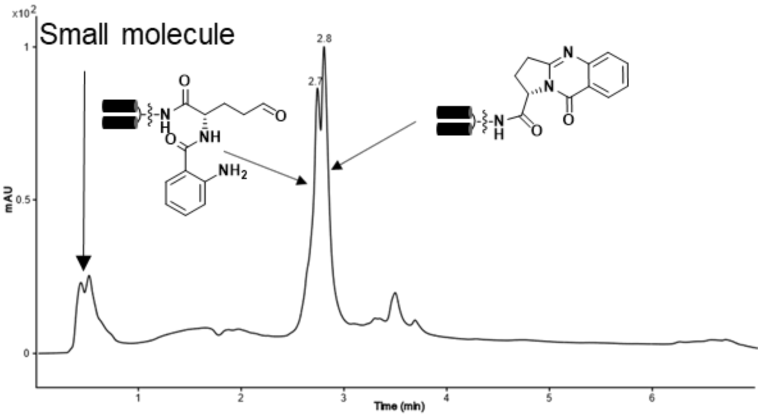

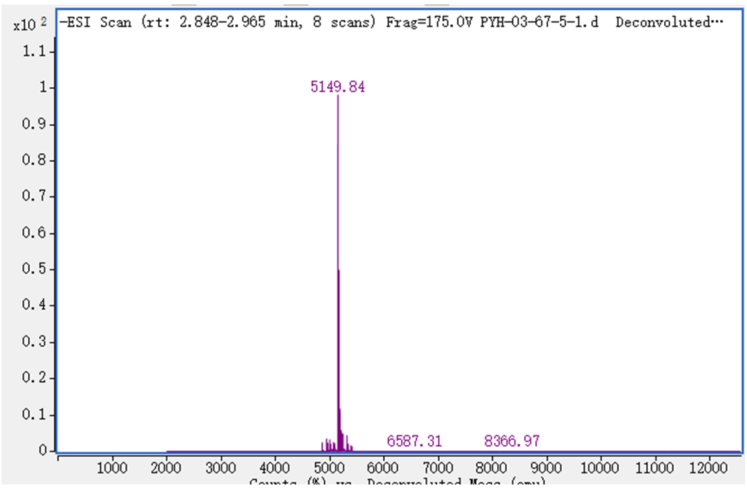

UPLC chromatogram and deconvoluted MS of **g3**

Conversion: 45%

Calculated Mass: 5167 Da; Observed Mass: 5167 Da

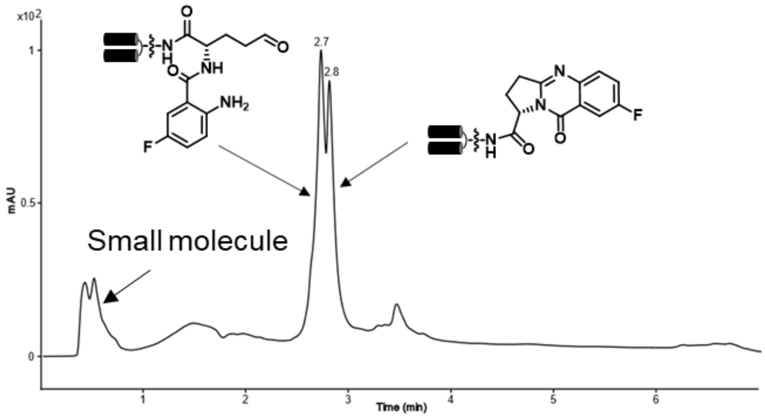

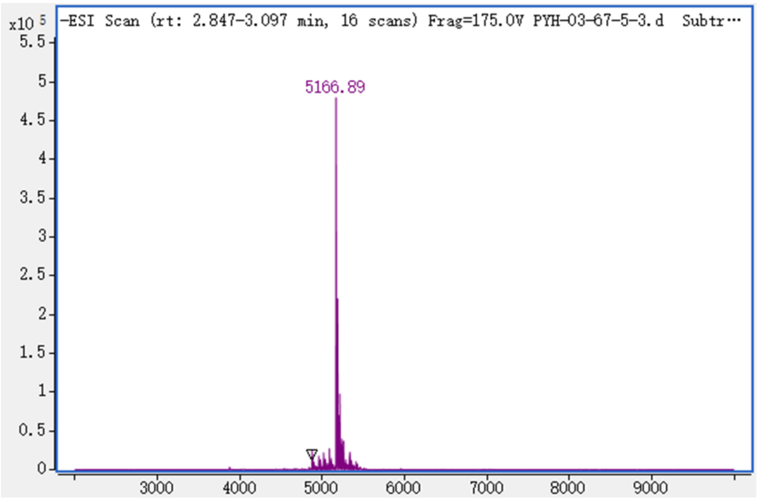

UPLC chromatogram and deconvoluted MS of **g4**

Conversion: 90%

Calculated Mass: 5191 Da; Observed Mass: 5191 Da

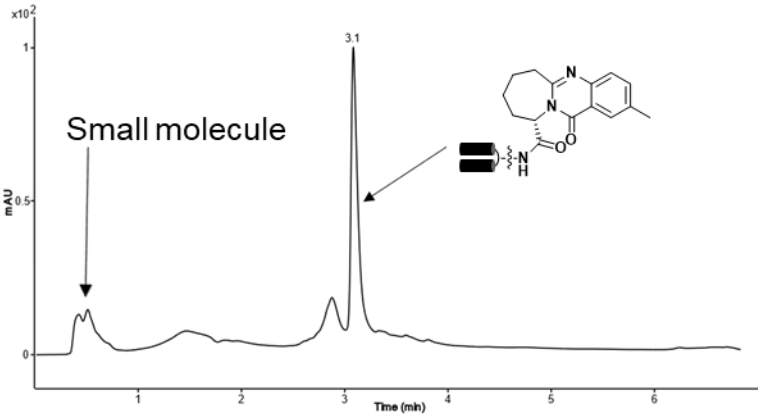

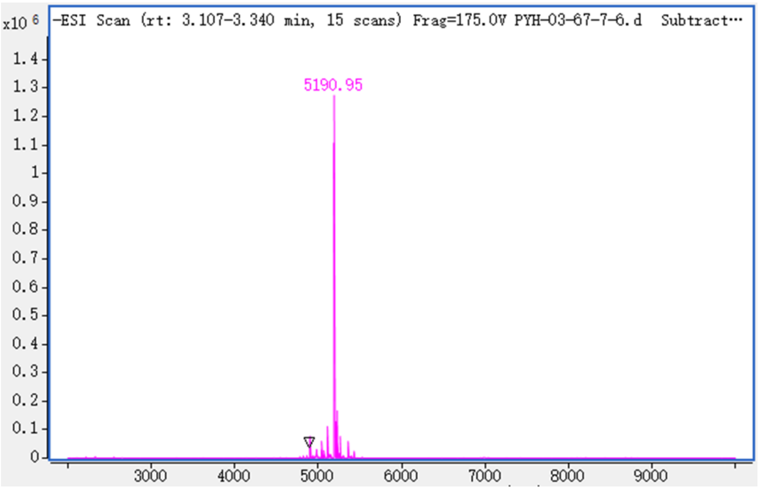

SUPPORTING INFORMATION

UPLC chromatogram and deconvoluted MS of **g5**

Conversion: 90%

Calculated Mass: 5177 Da; Observed Mass: 5177 Da

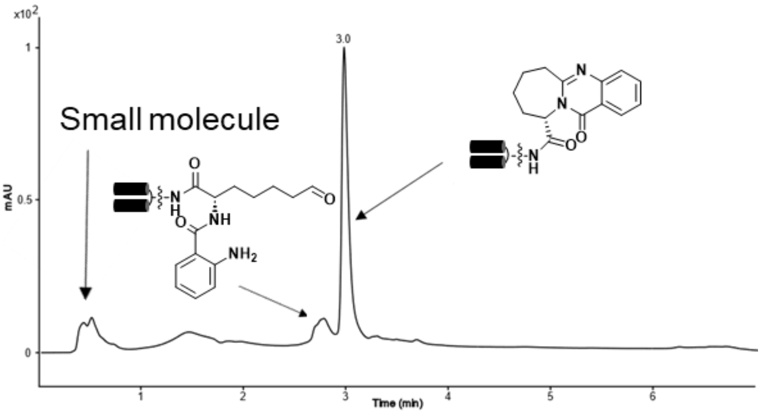

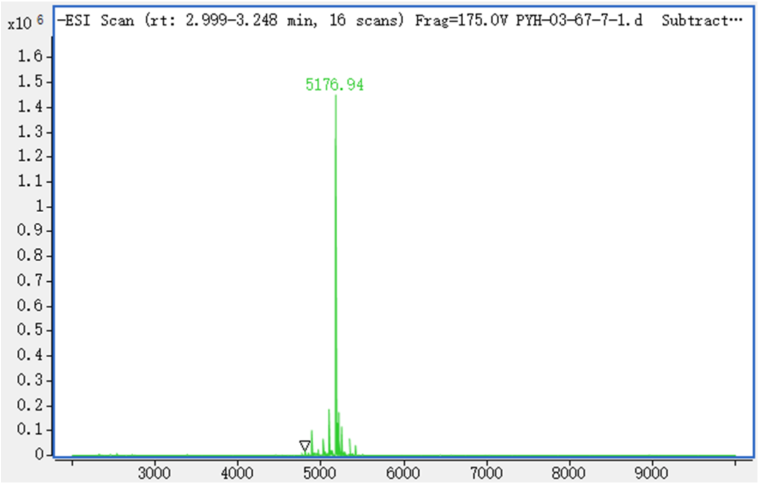

UPLC chromatogram and deconvoluted MS of **g6**

Conversion: 90%

Calculated Mass: 5195 Da; Observed Mass: 5195 Da

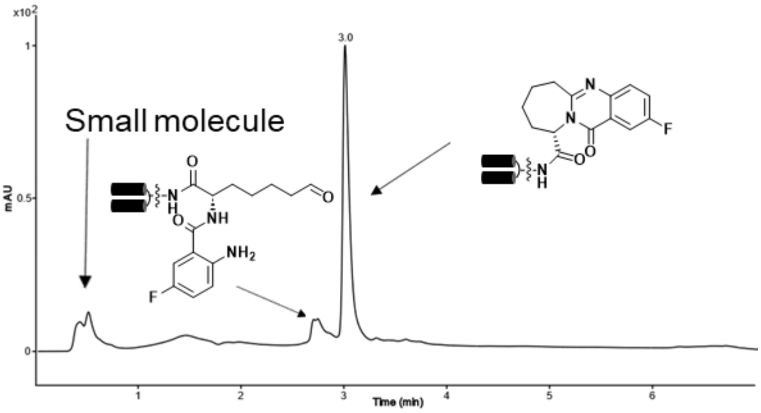

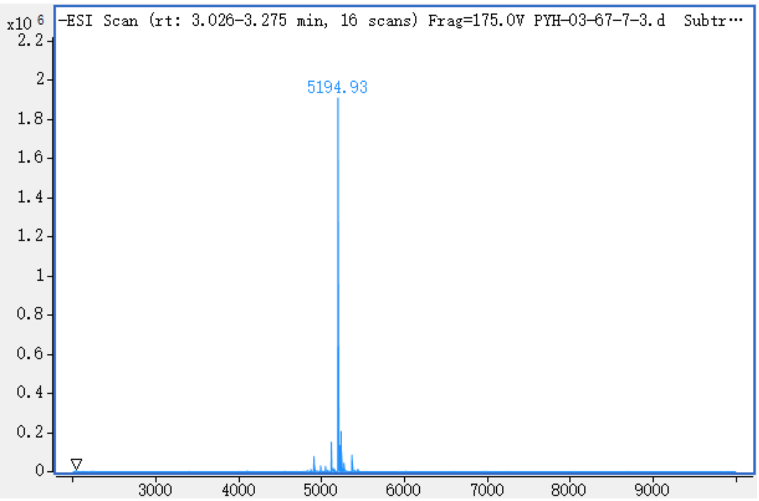

18. Compatibility of the oxidation reaction with DEL encoding

In figure 6d, DNA-CHO (200 pmol), code 13 nt (220 pmol, 1.1 equiv.), and 10× ligation buffer (2 μL) were added into a 0.6 mL tube and mixed by vortex. Then, T4 DNA ligase (1 μL, 350 units/μL) was added and mixed gently. The reaction mixture was vortexed, centrifuged, and incubated at 20 °C for 16 h. After ligation confirmation by UPLC-MS analysis, the reaction system was denatured by incubating at 95 °C in a dry bath for 10 min, and the ligation product was purified by ethanol precipitation. The resulting pellets were vacuum-dried and dissolved in nuclease-free water. Meanwhile, the raw headpiece-primer (**HP-P**) was treated in the same way as a positive control for the enzymatic ligation assay.

Code 60 nt sequences:

5'-AAC ACA GGC TTT GCT CGT ACA TAA AGC TCT TGC GTG GTC GTC TGA TGG CGC GAG GGA GGC-3'

5'-CTC CCT CGC GCC ATC AGA CGA CCA CGC AAG AGC TTT ATG TAC GAG CAA AGC CTG TGT TCA-3'

PCR primer sequences:

PCR1-F: 5'-GTT GGA AGC CAG CCC TCA GTG ACA GAG AAT ATG TGT AGA GGC TCG GGT GCT CTG-3'

PCR1-R: 5'-TCG TCG GCA GCG TCA GAT GTG TAT AAG AGA CAG GCC TCC CTC GCG CCA TCA GAC-3'

PCR2-F: 5'-AAT GAT ACG GCG ACC ACC GAG ATC TAC ACT CTT TCG TCT CGT GGG CTC GGA GAT G-3'

PCR2-R: 5'-TCG TCG GCA GCG TCA GAT GTG TAT AAG AGA CAG GCC TCC CTC GCG CCA TCA GAC-3'

19. Substrate scope of DNA-conjugated alcohols

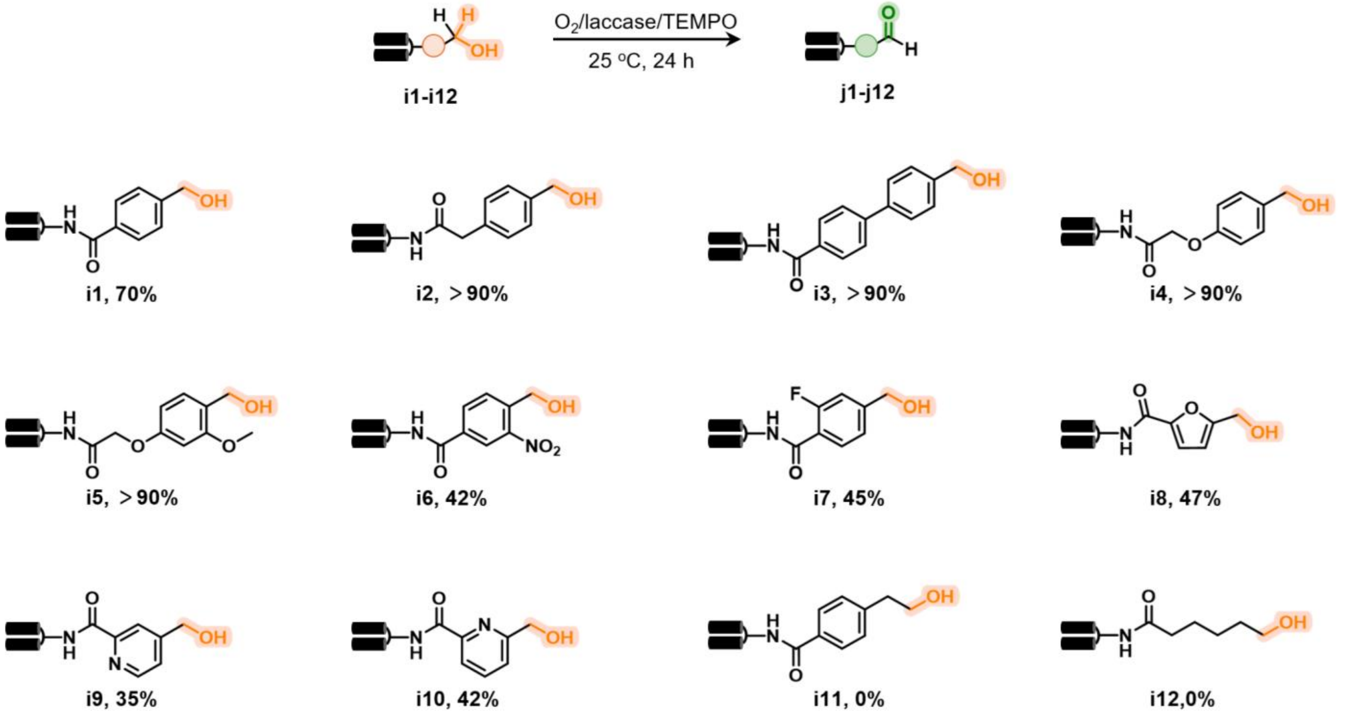

Figure S32. Substrate scope of DNA-conjugated alcohols.

DNA-conjugated alcohols (0.2 nmol, 1 equiv.) was added CH_3COONa buffer (16 µL, 200 mM, pH 4.0), Laccase (2 µL, 0.1 U/µL in H_2O), and TEMPO (2 µL, 400 mM in 1, 4-Dioxane, 800 nmol, 4000 equiv.). The reaction mixture was vortexed, centrifuged, and placed at 25 °C for 24 h. The product was obtained by ethanol precipitation and analyzed by UPLC-MS.

19.1 UPLC-MS Spectrum of DNA-conjugated alcohols

UPLC chromatogram and deconvoluted MS of **j1**

Conversion: 70%

Calculated Mass: 5069 Da; Observed Mass: 5069 Da

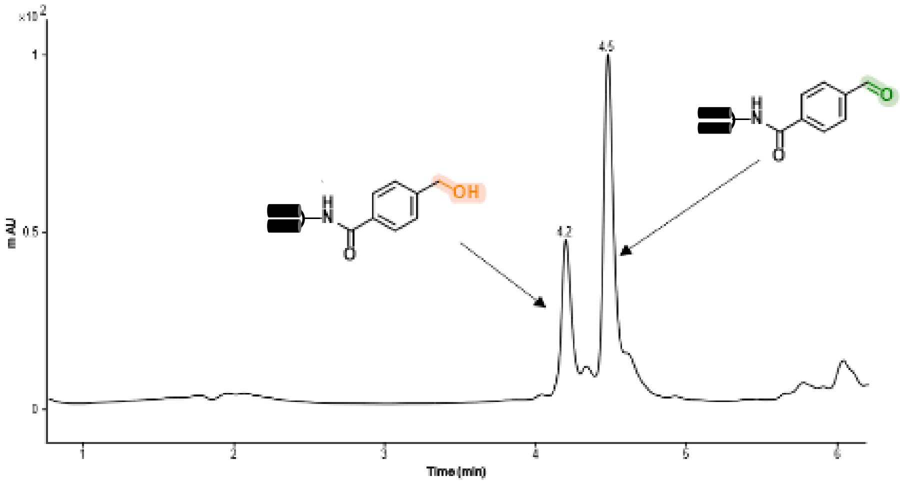

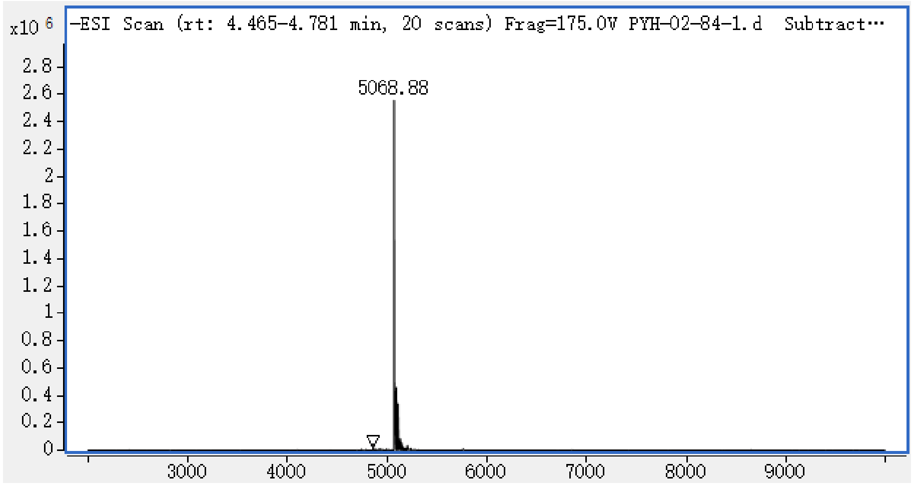

UPLC chromatogram and deconvoluted MS of **j2**

Conversion: ＞90%

Calculated Mass: 5083 Da; Observed Mass: 5083 Da

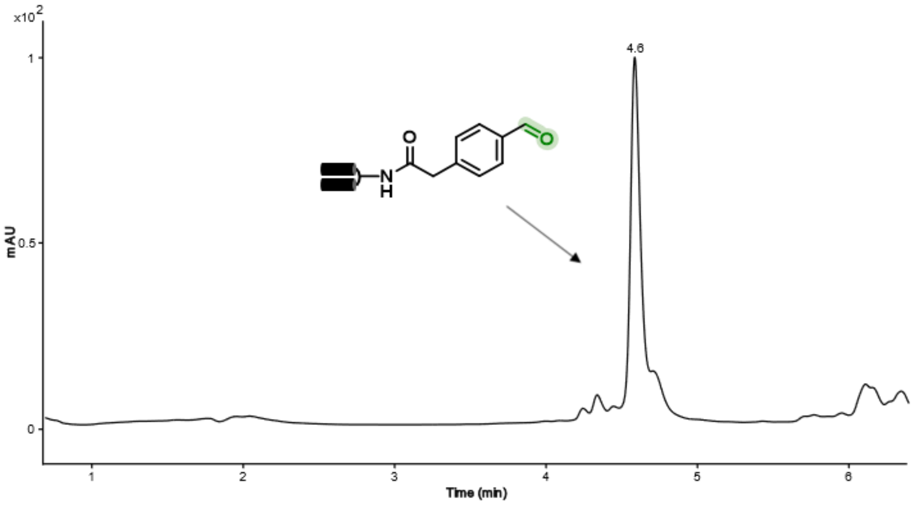

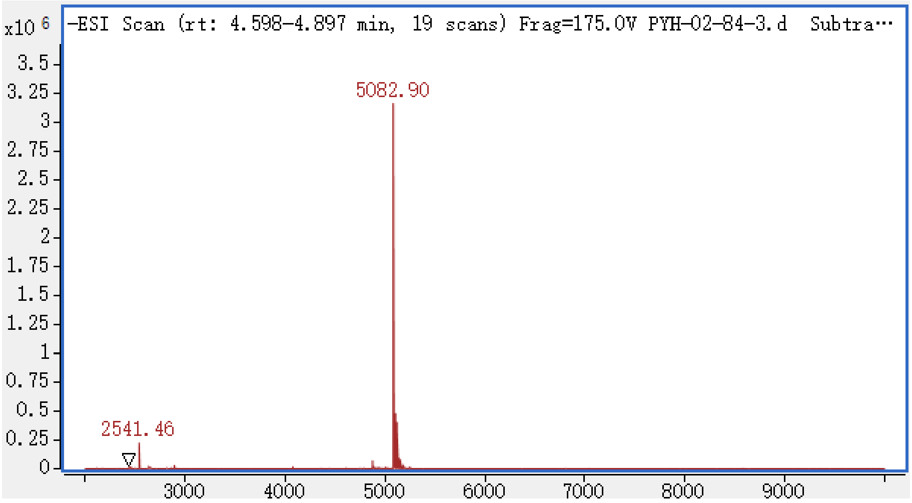

UPLC chromatogram and deconvoluted MS of **j3**

Conversion: ＞90%

Calculated Mass: 5145 Da; Observed Mass: 5145 Da

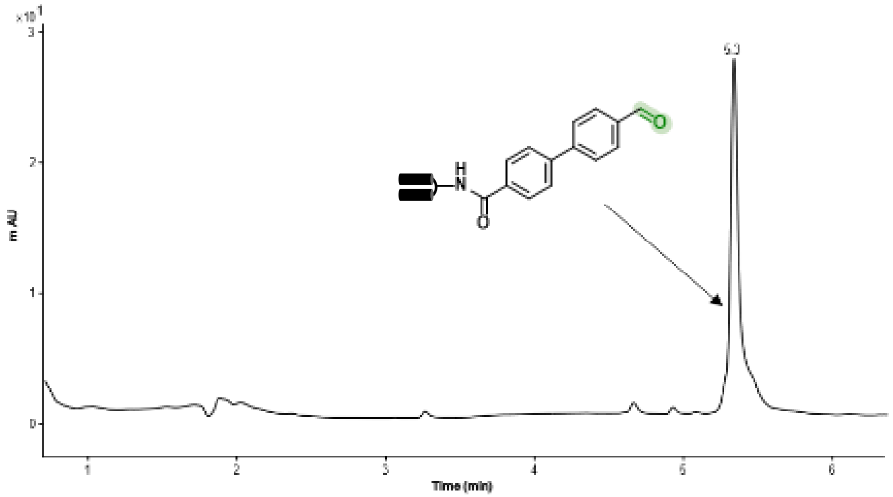

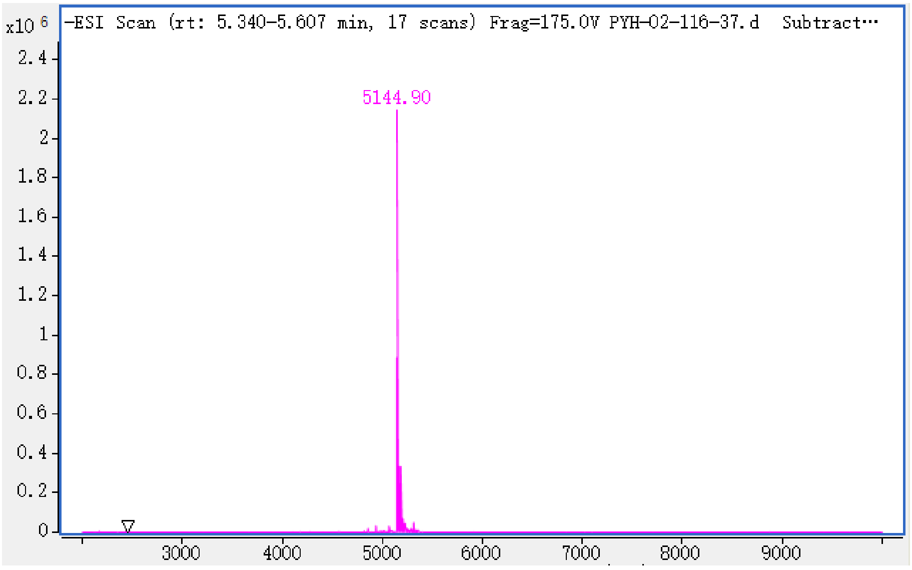

SUPPORTING INFORMATION

UPLC chromatogram and deconvoluted MS of **j4**

Conversion: ＞90%

Calculated Mass: 5099 Da; Observed Mass: 5099 Da

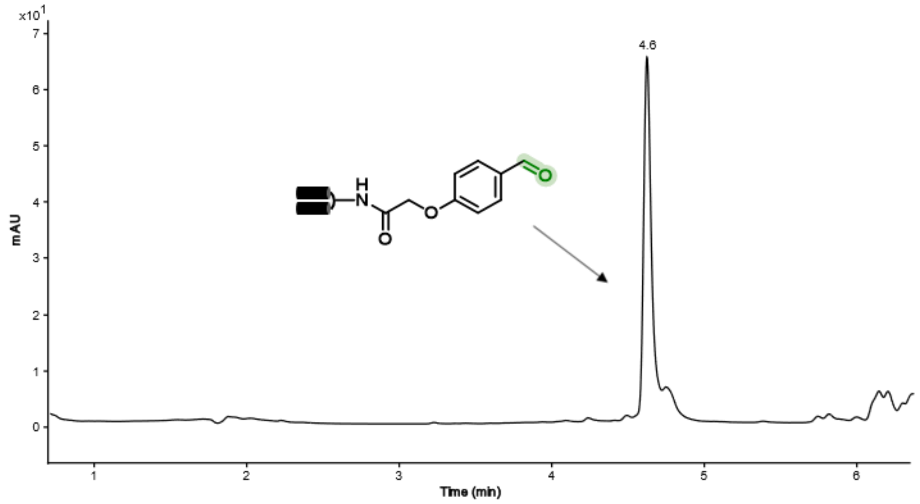

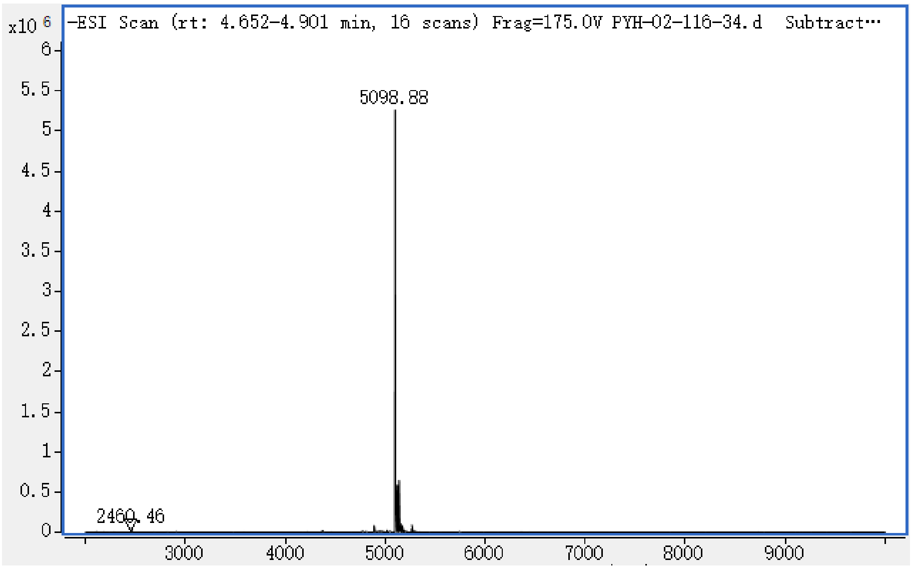

UPLC chromatogram and deconvoluted MS of **j5**

Conversion: ＞90%

Calculated Mass: 5129 Da; Observed Mass: 5129 Da

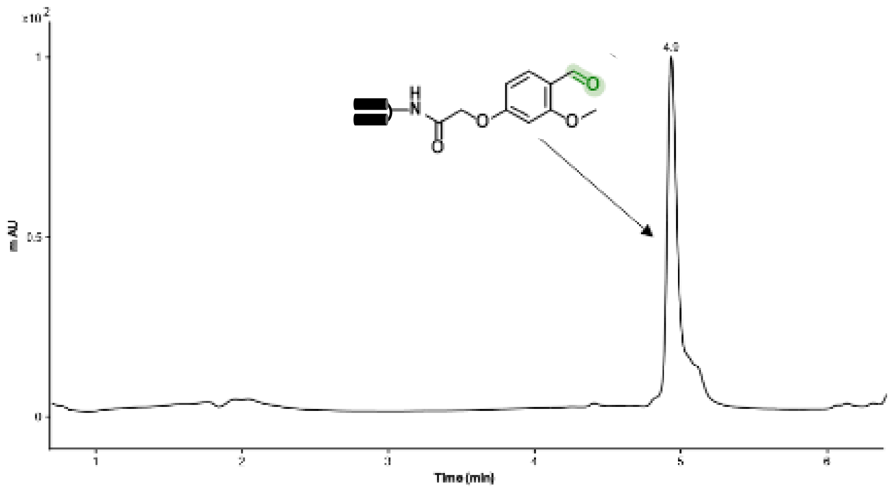

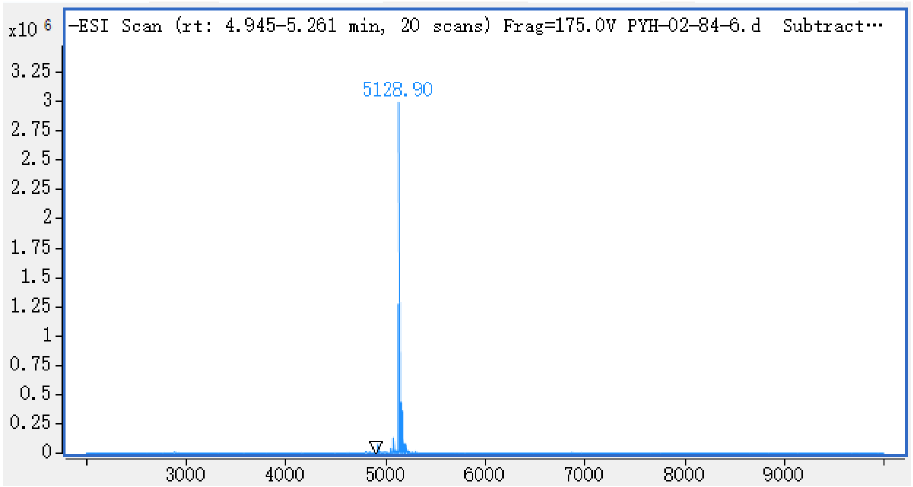

UPLC chromatogram and deconvoluted MS of **j6**

Conversion: 42%

Calculated Mass: 5114 Da; Observed Mass: 5114 Da

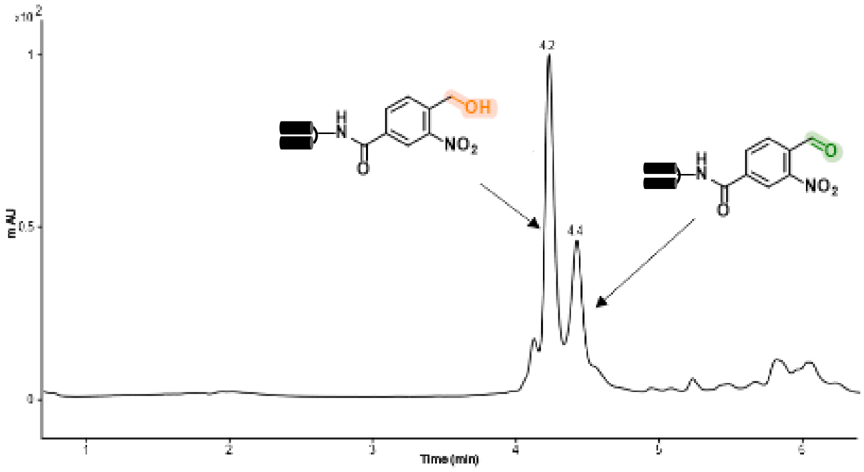

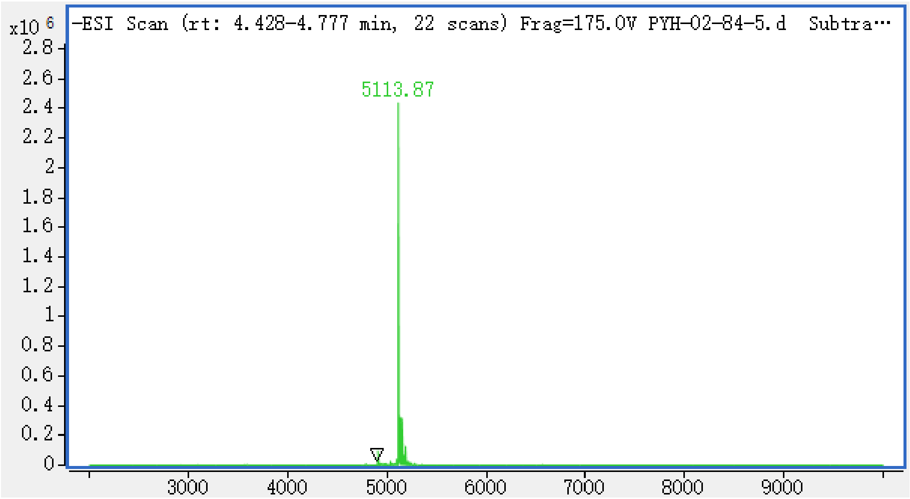

UPLC chromatogram and deconvoluted MS of **j7**

Conversion: 45%

Calculated Mass: 5086 Da; Observed Mass: 5086 Da

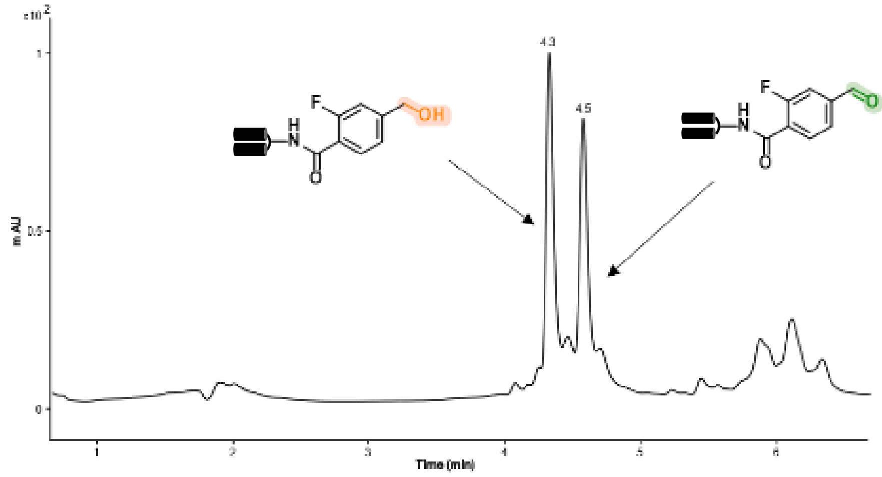

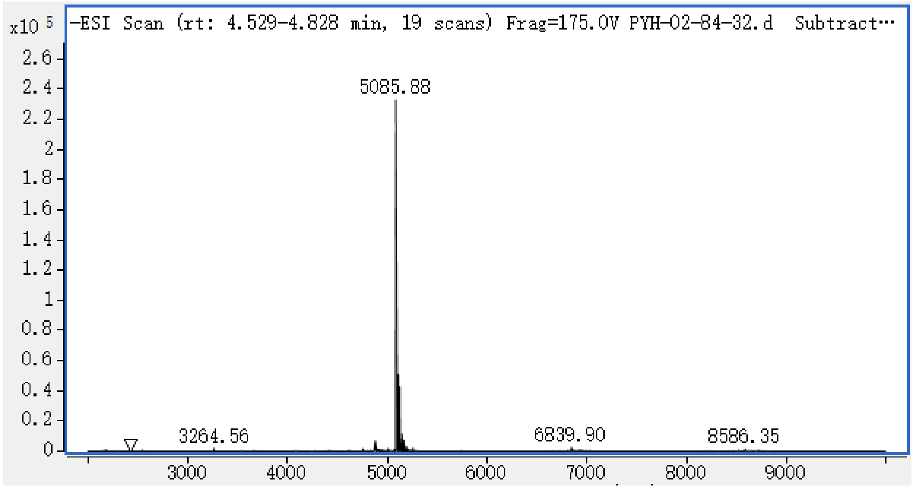

SUPPORTING INFORMATION

UPLC chromatogram and deconvoluted MS of **j8**

Conversion: 47%

Calculated Mass: 5058 Da; Observed Mass: 5058 Da

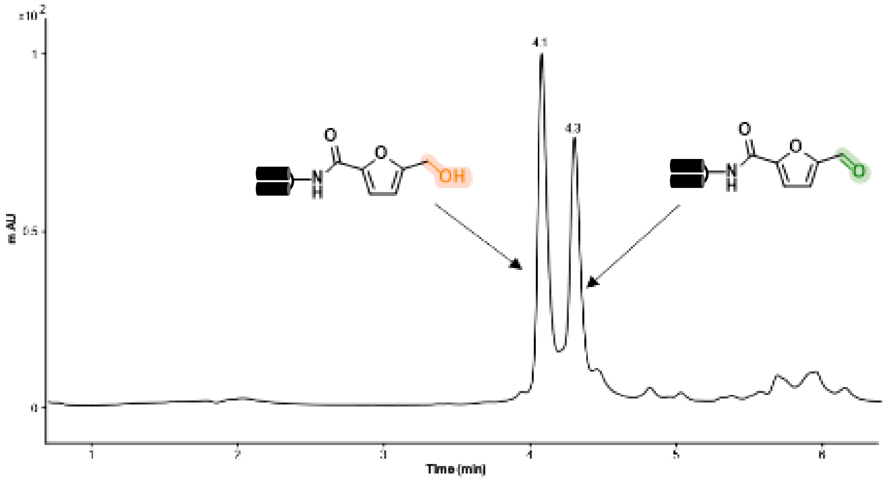

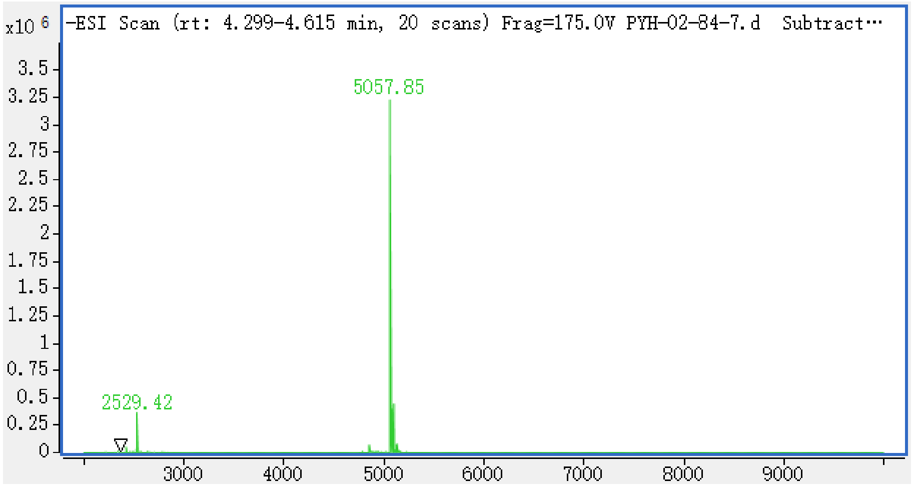

SUPPORTING INFORMATION

UPLC chromatogram and deconvoluted MS of **j9**

Conversion: 35%

Calculated Mass: 5070 Da; Observed Mass: 5070 Da

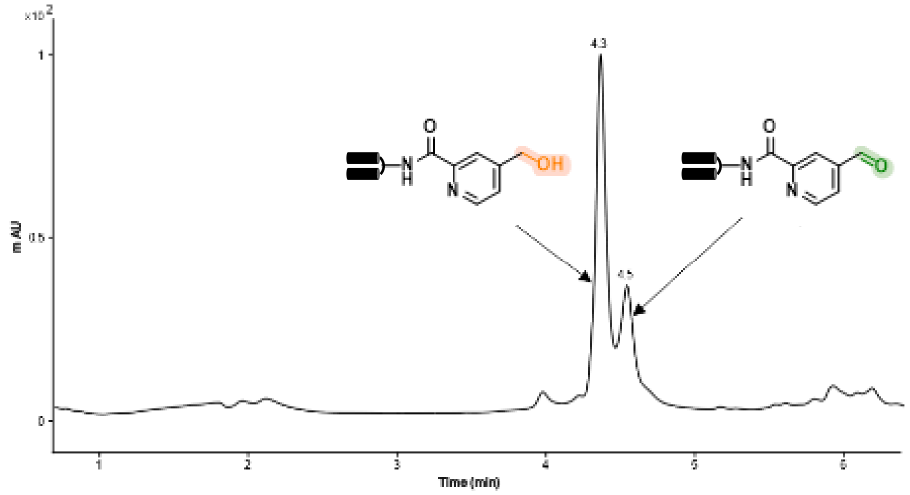

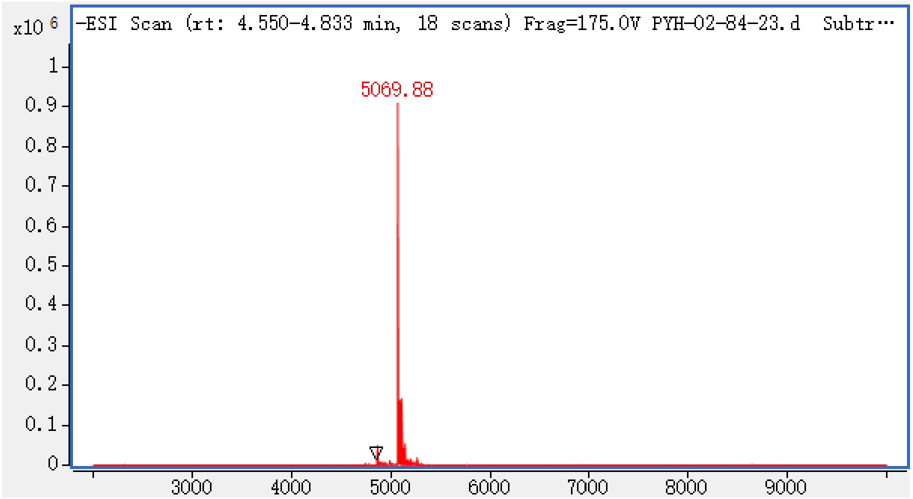

UPLC chromatogram and deconvoluted MS of **j10**

Conversion: 42%

Calculated Mass: 5070 Da; Observed Mass: 5070 Da

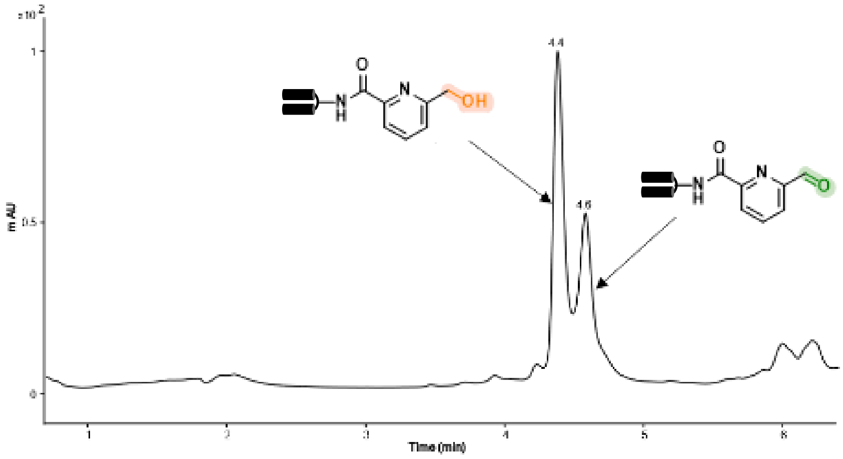

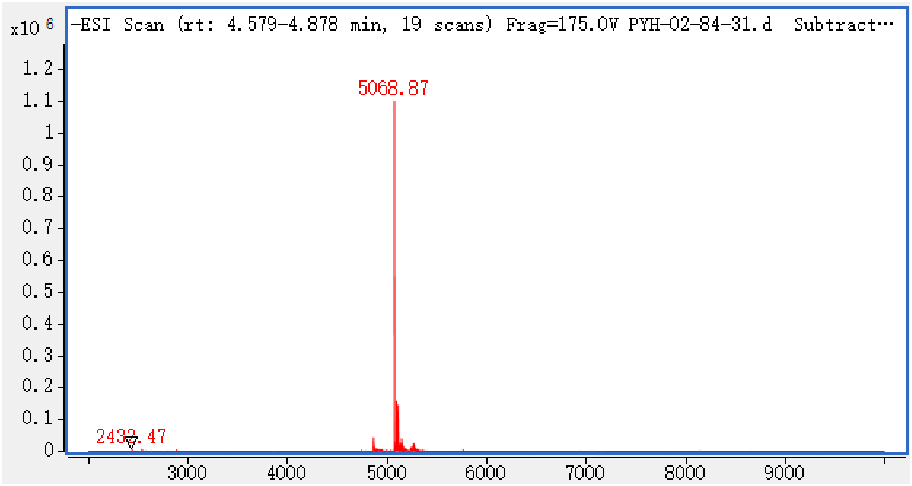

UPLC chromatogram and deconvoluted MS of **j11**

Conversion: 0%

Calculated Mass: 5083 Da; Observed Mass: 5085 Da

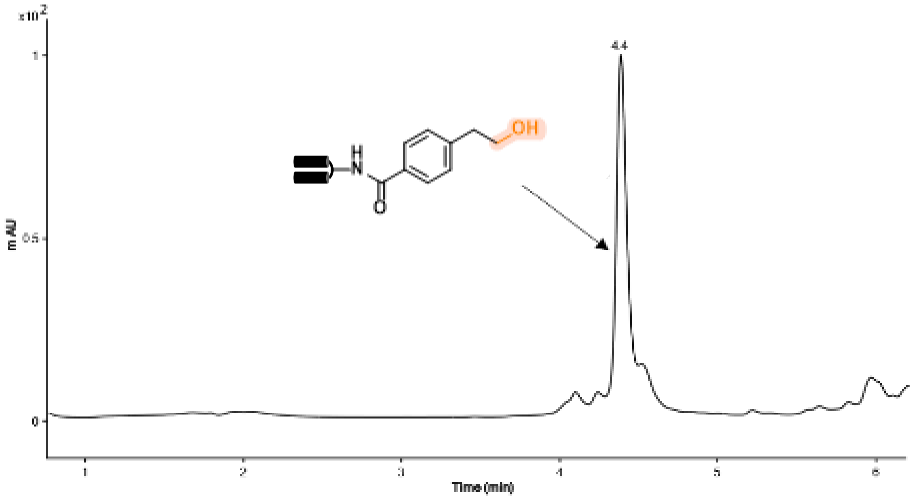

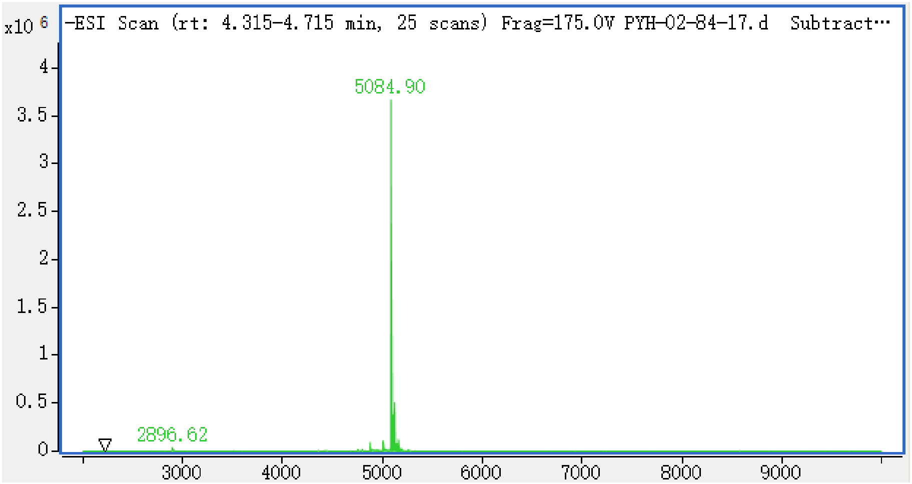

SUPPORTING INFORMATION

UPLC chromatogram and deconvoluted MS of **j12**

Conversion: 0%

Calculated Mass: 5049 Da; Observed Mass: 5051 Da

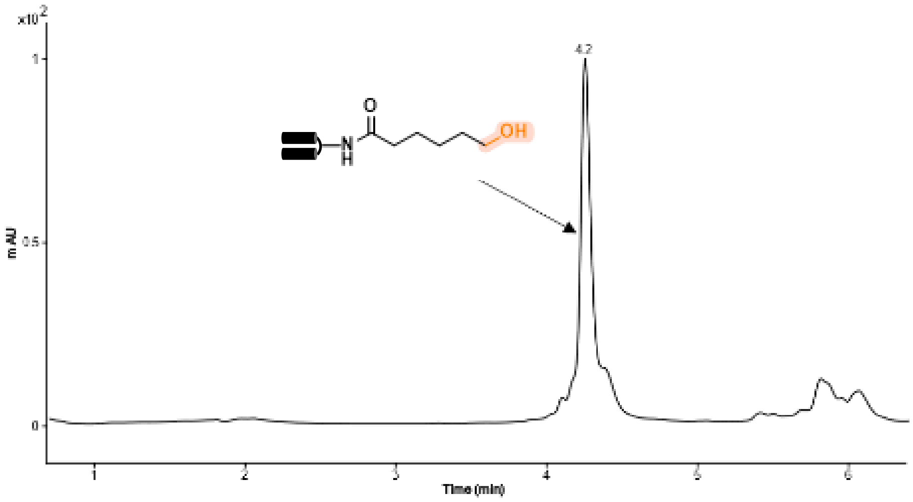

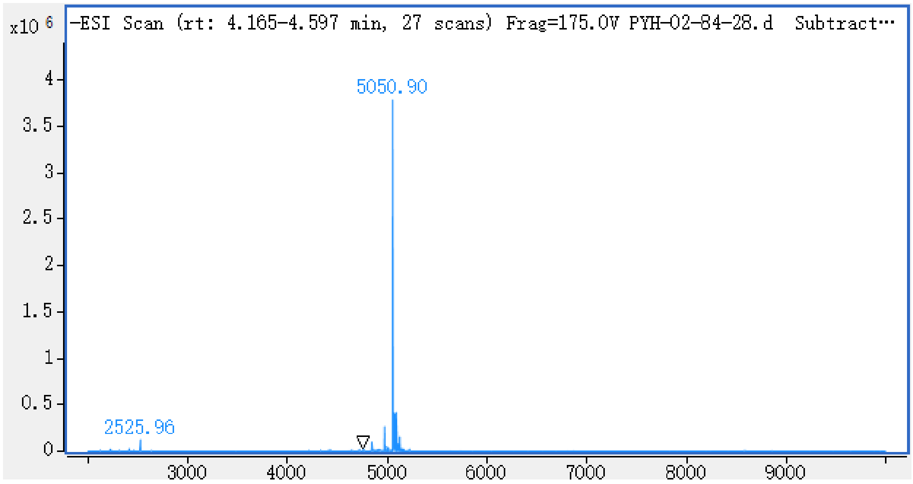

20. References

[1] Y. Li, M. Zhang, C. Zhang, X. Li, Detection of bond formations by DNA-programmed chemical reactions and PCR amplification, *Chem. Commun. (Camb.)* **2012**, *48*, 9513-9515.

[2] D. T. Flood, S. Asai, X. Zhang, J. Wang, L. Yoon, Z. C. Adams, B. C. Dillingham, B. B. Sanchez, J. C. Vantourout, M. E. Flanagan, D. W. Piotrowski, P. Richardson, S. A. Green, R. A. Shenvi, J. S. Chen, P. S. Baran, P. E. Dawson, Expanding Reactivity in DNA-Encoded Library Synthesis via Reversible Binding of DNA to an Inert Quaternary Ammonium Support, *J. Am. Chem. Soc.* **2019**, *141*, 9998-10006.

[3] Y. Qu, S. Liu, H. Wen, Y. Xu, Y. An, K. Li, M. Ni, Y. Shen, X. Shi, W. Su, W. Cui, L. Kuai, A. L. Satz, H. Yang, X. Lu, X. Peng, Palladium-mediated Suzuki-Miyaura Cross-Coupling Reaction of Potassium Boc-protected aminomethyltrifluoroborate with DNA-Conjugated aryl bromides for DNA-Encoded chemical library synthesis, *Biochem Biophys Res Commun* **2020**, *533*, 209-214.

[4] X. Ling, W. Lu, L. Miao, L. A. Marcaurelle, X. Wang, Y. Ding, X. Lu, Divergent On-DNA Transformations from DNA-Linked Piperidones, *J. Org. Chem.* **2022**, *87*, 1971-1976.

[5] H. Xu, J. Chen, J. Cheng, L. Kong, X. Chen, M. Inoue, Y. Liu, S. Kriaucionis, M. Zhao, C.-X. Song, Modular Oxidation of Cytosine Modifications and Their Application in Direct and Quantitative Sequencing of 5-Hydroxymethylcytosine, *Journal of the American Chemical Society* **2023**, *145*, 7095-7100.

[6] J. L. Merrifield, E. B. Pimentel, T. M. Peters-Clarke, D. J. Nesbitt, J. J. Coon, J. D. Martell, DNA-Compatible Copper/TEMPO Oxidation for DNA-Encoded Libraries, *Bioconjugate Chem.* **2023**, *34*, 1380-1386.

[7] P. He, G. Zhao, M. Zhu, Y. Li, G. Zhang, Y. Li, DNA-compatible functional group transformations via K2RuO4-mediated oxidation, *Organic Chemistry Frontiers* **2024**, *11*, 2851-2856.

[8] X. Fang, Y. Wang, P. He, H. Liao, G. Zhang, Y. Li, Y. Li, Visible Light-Promoted Divergent Benzoheterocyclization from Aldehydes for DNA-Encoded Chemical Libraries, *Org. Lett.* **2022**, *24*, 3291-3296.
